# The global, regional, and national burden of stomach cancer attributed to smoking in 204 countries, 1990–2019: A systematic analysis for the Global Burden of Disease Study 2019

**DOI:** 10.18332/tid/183803

**Published:** 2024-03-01

**Authors:** Fupeng Ren, Zhilong Shi, Xiu Shen, Gangfeng Xiao, Chaoying Zhang, Yiquan Cheng

**Affiliations:** 1 Department of Hematology and Oncology, Ningbo No. 2 Hospital, Ningbo, China

**Keywords:** Global Burden of Disease Study, stomach cancer, disability-adjusted life years, death, smoking

## Abstract

**INTRODUCTION:**

Understanding the current burden of stomach cancer linked to smoking and the variations in trends across different locations, is crucial for developing effective prevention strategies. In this study, we present findings on the age-standardized death rate (ASDR) and age-standardized disability-adjusted life years (DALYs) rate attributed to smoking in 204 countries and territories spanning 21 regions from 1990 to 2019.

**METHODS:**

The data for this study were obtained from the Global Burden of Disease Study (GBD) 2019, which assessed 369 diseases and injuries, as well as 87 risk factors in 204 countries and 21 regions. To assess the trend in ASDR and age-standardized DALYs rate, the estimated annual percentage change (EAPC) was utilized.

**RESULTS:**

Between 1990 and 2019, smoking was found to be associated with a decrease in ASDR (EAPC = -2.20) and age-standardized DALYs (EAPC = -2.42) rates for gastric cancer. As the sociodemographic index (SDI) increased, the decline in rates also increased gradually. However, the decline was smallest in regions with low SDI (EAPC_ASDR_ = -1.34; EAPC_age-standardized DALYs rate_ = -1.38). In 21 regions, both ASDR and DALYs rates experienced a decline. The smallest decline in ASDR was observed in Western Sub-Saharan Africa, with an EAPC of -0.80, while the smallest decline in DALYs rate was found in Oceania, with an EAPC of -0.81. Among the 204 countries analyzed, the Dominican Republic showed the highest increase in ASDR and age-standardized DALYs rate (EAPC_ASDR_ = 1.19; EAPC_age-standardized DALYs rate_ = 1.21), followed by Afghanistan (EAPC_ASDR_ = 1.09; EAPC_age-standardized DALYs rate_ = 1.09) and Sao Tome and Principe (EAPC_ASDR_ = 1.05; EAPC_age-standardized DALYs rate_ = 1.03). In the year 2019, the highest ASDR and age-standardized DALYs rate was observed in East Asia, with the highest rates occurring in Mongolia.

**CONCLUSIONS:**

The burden of stomach cancer worldwide, adjusted for age, and related to smoking, has shown a decline from 1990 to 2019. However, regional disparities have been identified, with some areas experiencing an increase in this burden. These regions with a higher burden emphasize the necessity for the implementation of strong tobacco control measures.

## INTRODUCTION

Stomach cancer, a well-known malignant tumor, carries a significant disease burden. It ranks among the top five largest malignant tumors worldwide^1^. In 2020, there were over 1.1 million new cases of stomach cancer and 76900 deaths attributed to this disease globally^2^. Consequently, stomach cancer has emerged as a significant contributor to the global cancer burden, posing a major challenge to public health efforts^3^.

Recently, stomach cancer has been classified as a tobacco-related cancer, and smoking greatly contributes to the development of stomach cancer^4^. There are multiple risk factors involved in the occurrence and progression of stomach cancer, including genetic factors, environmental factors, diet, smoking, and infection with *Helicobacter pylori* ^5-8^. While some of these risk factors are unchangeable, smoking is a modifiable behavioral risk factor, and it may be the primary factor responsible for the high rates of morbidity and mortality associated with early-stage stomach cancer^2^. However, few studies have examined and compared the burden of smoking-related stomach cancer on a global and national level.

We utilized the latest data from the Global Burden of Disease Study (GBD) in 2019 to systematically assess the trends in the age-standardized death rate (ASDR) and age-standardized Disability-Adjusted Life Years (DALYs) rate of stomach cancer attributed to smoking in 21 regions and 204 countries, from 1990 to 2019. The findings of this study will contribute to the development of prevention strategies and advancements in public health.

## METHODS

### Data sources

The GBD is conducted by the Institute for Health Metrics and Evaluation. GBD 2019 is considered the most comprehensive and detailed iteration to date^9,10^. A comprehensive description of the data collection and processing for GBD 2019, along with an overview of the methodology used to generate the results, has been published (https://vizhub.hcalthdata.org/gbd/results)^10,11^. The data used in this study stem from the GBD study conducted in 2019, which assessed exposure to 369 diseases and injuries, and 87 risk factors, across 204 countries and 21 regions from 1990 to 2019.

As part of a secondary analysis utilizing the GBD dataset, we conducted an extraction and analysis of data specifically pertaining to the burden of stomach cancer caused by smoking, comprising deaths and DALYs numbers, the ASDR^12^, and age-standardized DALYs rate^13^.

The Socio-Demographic Index (SDI) was widely regarded as a reliable indicator for assessing health-related socio-economic progress. It is a composite indicator of lag-distributed income per capita (lagdependent income per capita, and comprises the gross domestic product per capita that has been smoothed over the preceding 10 years), mean education for those aged ≥15 years (average years of schooling for the population older than 15 years of age), and total fertility rate under 25 years ( number of livebirths expected by age 25 years for a hypothetical woman who survived the age group and was exposed to current age-specific fertility rates), ranging from 0 to 1. To classify countries or territories, the SDI divides them into five quintiles: low (<0.46), low-middle (0.46–0.60), middle (0.61–0.69), high-middle (0.70–0.81), and high (>0.81) SDI regions^14^. The present study adheres to the Guidelines for Accurate and Transparent Health Estimates Reporting^9^.

### Case definition of stomach cancer

The identification of stomach cancer in the GBD study is performed using specific codes from the International Classification of Diseases (ICD). For ICD-9, the codes used are 151-151.9, 211.1, and 230.2. Meanwhile, for ICD-10, the codes used are C16-16.9, D00.2, D13.1, and D37.1.^10^

### Estimation framework

The GBD study utilized the conceptual framework of comparative risk assessment (CRA) to quantify the burden of stomach cancer caused by smoking. CRAs are valuable tools because they consolidate evidence from various sources focused on a specific factor that affects health, evaluate the relationship between this factor and the desired health outcome, and employ attribution strategies to determine the extent to which one cause contributes to an outcome influenced by multiple causes^10^.

The Cause of Death Ensemble model (CODEm) was used to estimate death rates and DALYs for stomach cancer, combining the sum of years lived with disability (YLDs) and years of life lost (YLLs) due to premature mortality^15^. This analysis focuses on data collected in 1990 and 2019, aiming to investigate the trends in the burden of stomach cancer over this time period. The selection of these years allows for a comprehensive comparison, thereby highlighting changes in death rates and the overall impact of stomach cancer on global health within the nearly three-decade interval.

### Statistical analysis

The aim of this study was to compare the impact of smoking on stomach cancer burden across various regions during a specific time period using the ASDR and age-standardized DALYs rate. Additionally, we assessed the trend in ASDR and age-standardized DALYs rate by calculating the estimated annual percentage change (EAPC). To achieve this, we employed a linear regression model, assuming a linear relationship:

*y = α + βx + ε*

between the natural logarithm of the age-standardized rate *y* and the calendar year *x*, where *ε* accounts for the random deviation, and *β* denotes the positive or negative trend in the age-standardized rate.

The EAPC was calculated using the formula:

EAPC = 100 × (exp(*β*) – 1).

The 95% confidence interval (CI) was obtained from the linear model. An increasing trend in the age-standardized rate was indicated when both the lower bound of the EAPC and its CI were above zero. Conversely, a decreasing trend was observed when the upper bounds of the EAPC and CI fell below zero. The estimation methods for disease burden in GBD study data were previously described^10^. All calculations were performed using R statistical software (version 3.6.1). A p<0.05 was considered statistically significant.

## RESULTS

### Global burden

**Figure 1:**
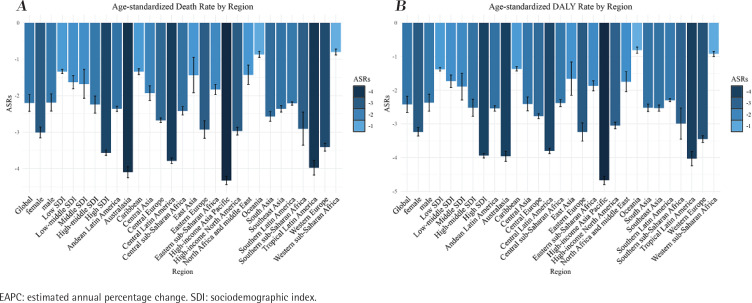
The EAPC of gastric cancer attributable to smoking, age-standardized rates (1990–2019)

**Figure 2:**
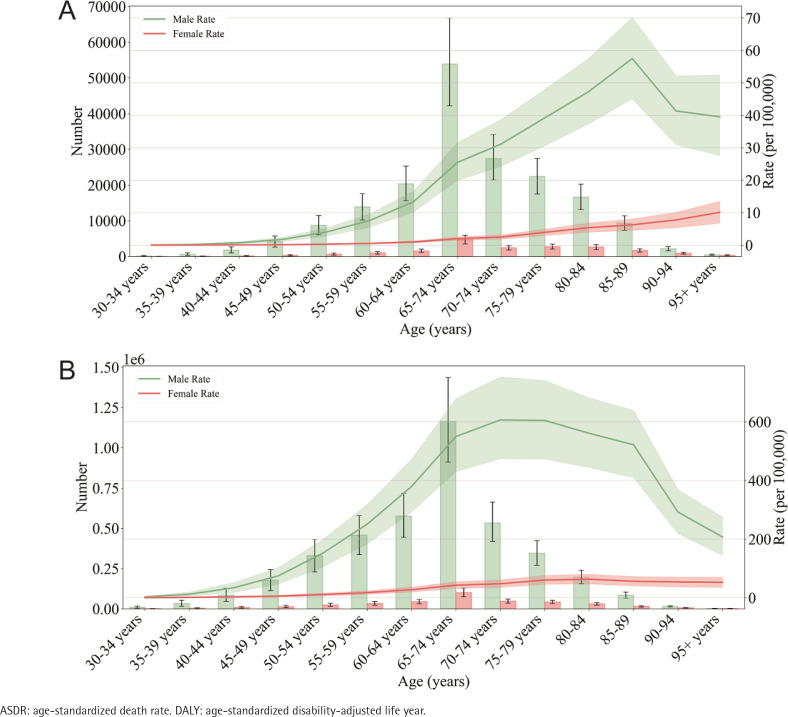
The ASDR and DALY rates of gastric cancer attributable to smoking in different age groups: A) Death cases, B) DALY cases

Globally, the number of deaths and DALYs due to stomach cancer increased by 13.04% and 2.88%, respectively, from 1990 to 2019. The ASDR for stomach cancer caused by smoking showed a decline during this period, with an EAPC of -2.20% (95% CI: -2.43 – -1.97) (Figure 1A, and Supplementary file Table 1). Similarly, the age-standardized DALYs rate also decreased, with an EAPC of -2.42 (95% CI: -2.66 – -2.18) (Figure 1B, and Supplementary file Table 2). These declining rates were observed in both sexes, with EAPCs in ASDR of -2.19 (95% CI: -2.42 – -1.95) in men and -3.01 (95% CI: -3.16 – -2.86) in women, and EAPCs in age-standardized DALYs rate of -2.37 (95% CI: -2.62 – -2.12) in men and -3.24 (95% CI: -3.36 – -3.11) in women. Furthermore, men had higher rates than women, as indicated by Figures 2A and 2B (Supplementary file Tables 1 and 2).

In 2019, the mortality rates associated with stomach cancer due to smoking were highest among individuals aged 65–69 years, regardless of gender (Figure 2A, and Supplementary file Table 1). In men, the ASDR started to rise in those aged 30–34 years and reached its peak in the those aged 74–79 years. Among women, the ASDR started to increase in those aged 35–39 years and peaked in those aged 80–84 years (Figure 2A). Furthermore, the ASDR exhibited an initial increase followed by a decrease with advancing age (Figure 2A). Notably, both the death numbers and the ASDR were lower for women compared to men (Figure 2A).

In 2019, the number of DALYs attributed to stomach cancer caused by smoking was highest in both men and women aged 70–74 years (Figure 2B, and Supplementary file Table 2). Among men, the DALY rate began to rise in the those aged 35–39 years and reached its peak in those aged 85–89 years. Among women, the DALY rate started to increase in those aged 55–59 years and reached its peak in those aged 80–84 years. The standardized DALYs rate initially increased and then decreased with increasing age (Figure 2B). Across all age groups, the standardized DALY rate was lower for women than for men (Figure 2B).

### Burden by SDI

The analysis shown in Supplementary file Table 1 and Supplementary file Figure 1 indicates that the decrease in the ASDR and age-standardized disability-adjusted life years (DALY) rate was least pronounced in regions with low SDI values. Specifically, the East Asia Pacific region displayed an EAPC for ASDR of -1.34 (95% CI: -1.39 – -1.28) and an EAPC for age-standardized DALY rate of -1.38 (95% CI: -1.43 – -1.33) (Supplementary file Tables 1 and 2, Figure 1). Furthermore, the ASDR and age-standardized DALY rate for stomach cancer attributable to smoking exhibited a consistent pattern across the five SDI regions. As the SDI increased, the decline in rates gradually intensified, with the magnitude of decrease varying as follows: high-SDI regions > medium-high-SDI regions > medium-low-SDI regions > low-SDI regions (Supplementary file Tables 1 and 2, Figure 1).

Supplementary file Figure 1 depicts the temporal trend of DALYs rates for ASDR in 21 regions classified based on the SDI from 1990 to 2019. In regions characterized as high-income, such as the High-Income Asia Pacific region, the ASDR exhibited a declining pattern; nevertheless, it consistently remained higher than the predicted level throughout the entire time period (Supplementary file Figure 1). Furthermore, the trend in age-standardized DALY rates for ASDR showed similar patterns across various SDI regions from 1990 to 2019 (Supplementary file Figure 1).

Supplementary file Figure 2A demonstrates that several countries, including Mongolia, China, Azerbaijan, and the Democratic People’s Republic of Korea, displayed significantly higher age-standardized DALY rates compared to what was predicted. Similarly, the relationship between SDIs and age-standardized DALY rates across different regions followed a similar trend, as shown in Supplementary file Figure 2B.

### Regional burden

In 2019, the regions with the highest ASDR for stomach cancer due to smoking were Eastern Europe, Central Asia, and East Asia, with ASDRs of 4.65, 2.60, and 2.58, respectively (Supplementary file Tables 1, 3 and 5). Between 1990 and 2019, the greatest decrease in ASDR was observed in the High-Income Asia Pacific region (EAPC = -4.33; 95% CI: -4.44 – -4.2), followed by Australasia (EAPC = -4.10; 95% CI: -4.25 – -3.95), and Tropical Latin America (EAPC = -3.98; 95% CI: -4.18 – -3.78) (Supplementary file Figure 1, Supplementary file Tables 1, 3 and 5).

In 2019, the highest age-standardized DALYs rate of stomach cancer caused by smoking per 100000 people was observed in East Asia (99.67), followed by Eastern Europe (66.51) and Central Asia (62.08) (Supplementary file Tables 2, 3 and 6). Over the period from 1990 to 2019, the age-standardized DALYs rate exhibited the largest decrease in the High-Income Asia Pacific region (EAPC = -4.67; 95% CI: -4.80 – -4.55), followed by Tropical Latin America (EAPC = -4.03; 95% CI: -4.24 – -3.82) and Australasia (EAPC = -3.96; 95% CI: -4.11 – -3.81) (Supplementary file Figure 1, Supplementary file Tables 2 and 4).

### National burden

**Figure 3:**
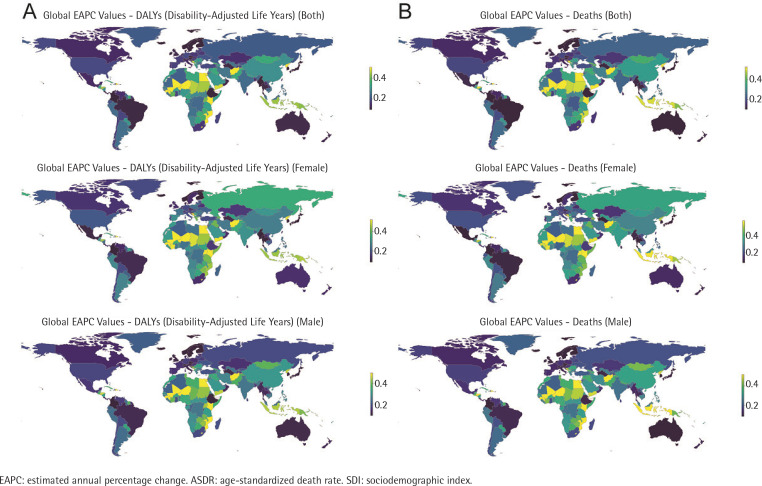
The EAPC of gastric cancer attributable to smoking, age-standardized rates, by region (1990–2019): A) The EAPC of ASDR, B) The EAPC of age-standardized DALY rate

In 2019, the countries with the highest ASDR for stomach cancer attributable to smoking were Mongolia, China, and Azerbaijan, with ASDRs of 6.84, 4.72, and 4.29, respectively (Supplementary file Tables 1, 4 and 5). From 1990 to 2019, the ASDR showed the greatest increase in the Dominican Republic (total EAPC = 1.19; 95% CI: 0.95–1.42; EAPC in men = 1.44; 95% CI: 1.19–1.68; EAPC in women = 0.73; 95% CI: 0.43–1.03), followed by Afghanistan (EAPC = 1.09; 95% CI: 0.98–1.21) and Sao Tome and Principe (EAPC = 1.05; 95% CI: 0.97–1.14). Singapore had the greatest decrease in the ASDR (total EAPC = -6.35; EAPC in men = -6.58; 95% CI: -6.57 – -6.13; EAPC in women = -5.32; 95% CI: -5.50 – -5.15), followed by the Republic of Korea (EAPC = -5.82; 95% CI: -6.07 – -5.57) and Colombia (EAPC = -5.00; 95% CI: -5.17 – -4.82) (Figure 3, and Supplementary file Tables 4 and 5).

In 2019, the three countries with the highest age-standardized DALYs rate of stomach cancer caused by smoking per 100000 people were Mongolia (154.53), the Democratic People’s Republic of Korea (102.69), and China (100.89). Conversely, the three countries with the lowest age-standardized DALYs rates were Nigeria (2.59), Ethiopia (3.69), and Ghana (5.04) (Supplementary file Tables 2, 4 and 6). Examining the period from 1990 to 2019, Singapore experienced the most significant decline in age-standardized DALYs rate (total EAPC = -6.72; 95% CI: 6.96 – -6.47; EAPC in men = -7.00; 95% CI: -7.21 – -6.78; EAPC in women = -5.61; 95% CI: -5.83 – -5.40). On the other hand, Afghanistan demonstrated the largest increase in age-standardized DALYs rate (total EAPC = 1.21; 95% CI: 1.08–1.33; EAPC in men = 1.51; 95% CI: 1.35–1.68; EAPC in women = 1.97; 95% CI: 1.87– 2.07) (Figure 3, and Supplementary file Tables 2, 4 and 6).

## DISCUSSION

This study is based on the GBD 2019, which provides the most up-to-date data on smoking-related stomach cancer deaths and DALYs. The findings of this study demonstrate significant variation in the spatial distribution of this disease burden across countries and regions. Between 1990 and 2019, both the global ASDR and age-standardized DALYs rate for smoking-related stomach cancer exhibited a decline, indicating improvements in prevention, control, and treatment of this condition due to smoking. Nevertheless, challenges persist in this regard.

From 1990 to 2019, the increase in the total number of deaths and DALYs was predominantly attributed to the growth and aging of the global population, particularly in East Asia, where there was a continuous rise in the absolute number of stomach cancer cases and related mortality^12^. However, both the ASDR and the age-standardized DALYs rate, exhibited a declining trend over this period. Two potential factors may account for this decline. Firstly, it could be attributed to an increased awareness of the detrimental effects of smoking and efforts to enforce smoking bans^16,17^. These efforts include raising awareness among healthcare professionals regarding the hazards of tobacco, as well as the implementation of measures by countries that have participated in the World Health Organization (WHO) Framework Convention on Tobacco Control to prohibit or regulate smoking^18^. Secondly, it may be due to changes in population demographics and advancements in the treatment of medical conditions, leading to improved outcomes.

The age-standardized rate of stomach cancer attributable to smoking is higher in men than in women, as indicated by the ASDR and age-standardized DALYs rate. Research has shown that since 1990, the prevalence of daily smoking has been approximately five times higher in men compared to women (25% vs 5.4%)^19^. It is also widely observed that smoking is more common among males than females^20^. The impact of tobacco carcinogens directly affects the occurrence of stomach cancer, while the existence of *H. pylori* may have an indirect impact^19,^ ^20^. Sociocultural factors play a significant role in the difference between men and women regarding smoking and drinking behavior. Men tend to be more influenced by cultural factors, leading to higher rates of smoking and alcohol consumption. Furthermore, men are more likely to be exposed to environmental or occupational factors that increase the risk of *H. pylori* infection compared to those without such exposures^19,21^. The association between *H. pylori* infection and stomach cancer is stronger than initially expected^22^. The pathophysiological mechanisms of estrogen may have a protective effect against stomach cancer, which could potentially explain the sex-related differences in the incidence of the disease^23-25^.

The highest global burden of stomach cancer caused by smoking occurred in those aged 74–89 years^26^. Another study discovered that the trend in the global stomach cancer burden follows a pattern of increasing and then decreasing with age, reaching its peak in elderly individuals. Smoking-related cancer develops through chronic accumulation, as the quantity and duration of smoking contribute to the build-up of carcinogens in the body. Elderly individuals often experience declines in physiological and cognitive functions and face a higher risk of comorbidity^27^, making early detection of stomach cancer challenging in this age group. Consequently, stomach cancer is more likely to result in mortality among elderly individuals than younger individuals. Therefore, early detection and treatment of stomach cancer among the elderly to reduce mortality represent a significant challenge, and early screening plays a crucial role in clinical practice. Additionally, this study found that the ASDR started increasing in men aged 30–34 years and women aged 35–39 years. Thus, it is imperative to strengthen tobacco control measures targeting young people to decrease the number of youths exposed to tobacco.

The relationship between the socio-economic status (as measured by the SDI value) and the burden of stomach cancer caused by smoking was investigated. It was observed that regions with a lower SDI value had an increased burden of stomach cancer, whereas regions with a higher SDI value had a decreased burden. The increase in burden in regions with a lower socio-economic status may be attributed to factors such as poor environmental conditions, a high smoking rate, and limited access to treatment options. Additionally, population growth and aging may contribute to the overall disease burden, thereby offsetting the potential decrease resulting from smoking reduction efforts. On the other hand, areas with a higher socio-economic status experienced a reduction in the burden, which can be attributed to advancements in medical technology, scientific education, and effective prevention and control measures promoting a healthy lifestyle^18,28^.

There were significant disparities in the ASDR and age-standardized DALYs rates among countries and regions, which could be attributed to variations in the levels of exposure to risk factors. In 2019, the region of East Asia reported the highest ASDR and rate of age-standardized DALYs. Being the most populous country in East Asia, China has witnessed economic growth accompanied by an increase in affluence and leisure time, thereby fostering a positive emotional association with smoking and subsequently driving up smoking rates^23^. Mongolia demonstrated the highest prevalence of smoking among all East Asian countries, with rates of 50% for men and 5% for women. As the population structure undergoes changes, the disease burden associated with smoking escalates with time, prevailing smoking rates, and intensity of smoking habits.

The region and country with the lowest ASDR were Western Sub-Saharan Africa and Nigeria, respectively. This may be attributed to the absence of a cancer registry in these African countries^22^, as well as a lower smoking rate compared to other regions^19^. Another study found that the low cancer burden in African countries was related to their accession to the WHO Tobacco Control Treaty^28^. The regions and countries that experienced the largest reduction in ASDR and rate of age-standardized DALYs (disability-adjusted life years) were the High-Income Asia Pacific region and Singapore, respectively.

This can be attributed to the implementation of smoking restrictions aimed at improving public health and easing the burden of disease. The period from 2005 to 2015 marked the first decade of implementing the WHO Tobacco Control Treaty. The decline in smoking rates during this period was closely correlated with the tobacco control measures recommended by the Framework Convention on Tobacco Control, although there were significant variations in the efforts made by countries and regions to control tobacco^29^. The high burden of disease in two low- and middle-income countries, the Dominican Republic and Afghanistan, may be attributed to limited medical and public health systems stemming from economic constraints, a lack of intervention measures, and poor lifestyle choices that increase tobacco exposure. Therefore, in order to further mitigate the harmful effects of tobacco use, it is necessary to adopt more comprehensive and effectively targeted policies than the existing ones.

Data were extracted from the GBD database and subjected to standardized processing in order to estimate the time-change trend (expressed as EAPC) in the burden of stomach cancer associated with smoking over the past 30 years. Additionally, the regional and national distribution trends of this burden were assessed and compared from a global perspective. Our findings significantly enhance our understanding of the burden of stomach cancer attributed to smoking and the global variations observed in this burden. Furthermore, these findings will facilitate the identification of priority actions for global prevention efforts. Specifically, they will provide valuable insights for the development of targeted strategies in future stomach cancer prevention and tobacco control initiatives within different regions.

### Limitations

This study has several limitations. Firstly, the lack of cancer registration centers in certain low-income areas and nations has led to limited data on stomach cancer caused by smoking. Consequently, the estimation of disease burden trends may have been affected. However, efforts were made to standardize the research data to ensure data quality. Secondly, the study did not take into account the impact of secondhand smoke and passive smoking on the risk of stomach cancer. As a result, the burden of stomach cancer caused by tobacco may be significantly higher than what was evaluated, and the trends may differ. Thirdly, our study only focused on the burden of stomach cancer caused by smoking, while neglecting other confounding factors such as *H. pylori* infection, genetics, high-sodium diet, and environmental factors^30^. However, the combined effect of smoking and other risk factors may increase the burden of stomach cancer or complicate the data on this burden. Furthermore, since our study was conducted at the population level, there is a potential risk of ecological fallacy. Therefore, the relationship between mortality, DALYs, and sociodemographic indicators, although indicative, should not be interpreted as causal. Moreover, as a secondary analysis of the GBD data, our ability to adjust for biases related to demographic variables such as race, education, and occupation is limited. Lastly, the assumption of a linear association between ln (rate) and time, as well as the use of only two data points to estimate trends over a long time period, are undeniable limitations.

## CONCLUSIONS

There are notable geographical disparities in the distribution and trajectory of the disease burden of stomach cancer resulting from smoking. While the global burden of stomach cancer attributable to smoking, as standardized for age, has declined between 1990 and 2019, certain regions have witnessed a surge in the age-standardized burden of this disease. Particularly, certain areas in East Asia and Eastern Europe, among other specific locations, have experienced an increase in the age-standardized burden of stomach cancer linked to smoking. These regions are characterized by a high prevalence of the ailment and necessitate urgent implementation of robust tobacco-control measures in order to mitigate the impact of smoking-related stomach cancer.

## Supplementary Material



## Data Availability

The data supporting this research are available from the following link: http://ghdx.healthdata.org/gbd-results-tool.
