# Supplementary material for: The global, regional, and national burden of stomach cancer attributed to smoking in 204 countries, 1990–2019: A systematic analysis for the Global Burden of Disease Study 2019
Source: Tob Induc Dis. 2024 Mar 1;22:10.18332/tid/183803. doi: 10.18332/tid/183803 (PMC10907929; doi:10.18332/tid/183803)
Supplement: Supplementary file 1 [file TID-22-48-s1.pdf]

Tables

Supplementary table 1. The age-standardized death rate (ASDR) of gastric cancer attributable to smoking in 1990 and 2019 and its temporal trends

Characteristics	1990		2019		EAPC (1990-2019)
	death cases	ASDR per 10000	death cases	ASDR per 10000	ASDR
	No.(95%UI)	No.(95%UI)	No.(95%UI)	No.(95%UI)	No.(95%CI)
**Global**	152082.38(123653.51,181845.82)	3.94(3.21,4.69)	171920.37(137763.81,206661.62)	2.12(1.70,2.55)	-2.20(-2.43, -1.97)
**Sex**	-	-	-	-	-
female	18390.86(14306.53,22534.40)	0.89(0.69,1.09)	16911.39(12818.88,21035.26)	0.39(0.29,0.48)	-3.01(-3.16, -2.86)
male	152082.38(123653.51,181845.82)	7.78(6.36,9.20)	155008.98(124000.55,186810.72)	4.18(3.37,5.03)	-2.19(-2.42, -1.95)
**Sociodemographic index**	-	-	-	-	-
Low SDI	2233.39(1533.90,2942.52)	1.05(0.74,1.36)	3324.28(2351.82,4390.85)	0.71(0.52,0.92)	-1.34(-1.39, -1.28)
Low-middle SDI	12673.04(9716.44,15529.90)	2.30(1.79,2.78)	18685.15(14478.62,22959.82)	1.45(1.13,1.76)	-1.63(-1.81, -1.45)
Middle SDI	43511.73(33587.65,54564.91)	4.46(3.47,5.51)	65027.80(50373.62,80126.07)	2.72(2.12,3.32)	-1.68(-2.07, -1.28)
High-middle SDI	55918.86(45340.20,67275.98)	5.23(4.26,6.27)	58746.06(46215.99,71492.30)	2.85(2.24,3.46)	-2.24(-2.48, -2.01)
High SDI	37703.73(31371.74,43729.99)	3.57(2.98,4.15)	26089.68(20932.27,31317.93)	1.31(1.05,1.57)	-3.57(-3.64, -3.49)
**Region**	-	-	-	-	-
Andean Latin America	490.87(337.02,664.32)	2.57(1.80,3.45)	692.22(448.10,985.36)	1.28(0.84,1.81)	-2.36(-2.43, -2.29)

Australasia	381.05(306.98,455.98)	1.60(1.29,1.92)	251.12(193.21,309.24)	0.51(0.39,0.62)	-4.10(-4.25, -3.95)
Caribbean	404.69(319.21,492.38)	1.60(1.26,1.94)	521.70(395.89,657.38)	1.01(0.77,1.27)	-1.34(-1.42, -1.26)
Central Asia	2241.01(1815.79,2662.33)	4.70(3.83,5.58)	1865.90(1481.76,2261.14)	2.58(2.07,3.07)	-1.93(-2.13, -1.73)
Central Europe	6374.02(5173.81,7463.19)	4.26(3.47,4.98)	4361.21(3405.71,5346.50)	2.01(1.57,2.46)	-2.68(-2.74, -2.61)
Central Latin America	2154.24(1624.29,2705.47)	2.81(2.15,3.52)	2256.33(1526.68,3061.05)	0.98(0.67,1.34)	-3.79(-3.86, -3.71)
Central sub-Saharan Africa	202.52(119.67,295.75)	0.95(0.58,1.36)	249.31(150.19,374.15)	0.50(0.31,0.72)	-2.42(-2.53, -2.30)
East Asia	60050.36(45495.37,75925.92)	7.13(5.46,8.93)	95818.54(72687.84,120868.06)	4.65(3.53,5.83)	-1.44(-1.92, -0.95)
Eastern Europe	15178.61(12141.77,17991.27)	5.28(4.26,6.27)	9019.70(7053.35,11021.62)	2.60(2.04,3.19)	-2.93(-3.17, -2.69)
Eastern sub-Saharan Africa	550.85(316.42,788.43)	0.81(0.49,1.15)	724.30(426.81,1034.16)	0.50(0.30,0.70)	-1.83(-1.97, -1.69)
High-income Asia Pacific	17655.21(14690.22,20461.41)	8.82(7.33,10.23)	12409.16(9832.20,14996.99)	2.58(2.05,3.09)	-4.33(-4.44, -4.22)
High-income North America	5411.15(4416.29,6453.37)	1.51(1.23,1.79)	4215.82(3358.08,5167.31)	0.66(0.53,0.80)	-2.97(-3.08, -2.87)
North Africa and middle East	4026.49(3192.75,4881.76)	2.47(1.95,2.97)	6151.28(4892.50,7488.83)	1.55(1.23,1.89)	-1.43(-1.69, -1.16)
Oceania	57.33(36.66,79.37)	2.06(1.39,2.81)	102.04(60.38,153.44)	1.53(0.96,2.20)	-0.87(-0.95, -0.78)
South Asia	6948.55(4963.86,8890.15)	1.42(1.05,1.78)	9353.09(6632.21,12325.54)	0.72(0.51,0.93)	-2.57(-2.70, -2.44)
Southeast Asia	4620.18(3458.97,5821.40)	1.96(1.49,2.44)	5964.28(4458.70,7501.82)	1.06(0.80,1.31)	-2.36(-2.45, -2.27)
Southern Latin America	1741.42(1380.63,2084.82)	3.75(2.98,4.49)	1625.56(1268.72,1973.22)	1.94(1.51,2.35)	-2.21(-2.26, -2.17)
Southern sub-Saharan Africa	414.57(310.75,518.69)	1.59(1.19,1.96)	406.01(299.99,515.05)	0.76(0.57,0.96)	-2.91(-3.36, -2.45)

Tropical Latin America	3974.41(3180.09,4768.95)	4.68(3.77,5.58)	3546.91(2747.66,4336.20)	1.49(1.16,1.83)	-3.98(-4.18, -3.78)
Western Europe	18682.35(15507.98,21858.43)	3.14(2.60,3.67)	11587.64(9254.29,13929.90)	1.23(0.98,1.47)	-3.41(-3.52, -3.31)
Western sub-Saharan Africa	522.49(337.77,725.98)	0.64(0.43,0.89)	798.25(523.98,1112.75)	0.48(0.32,0.66)	-0.80(-0.88, -0.72)

Note: ASDR, age-standardized death rate; DALY, age-standardized disability-adjusted life year; EAPC, estimated annual percentage change; UI is the uncertainty interval,which reflects the certainty of an estimate based on data availability, study size, and consistency across data sources.

Supplementary table 2. The age-standardized DALY rate of gastric cancer attributable to smoking in 1990 and 2019 and its temporal trends

Characteristics	1990		2019		EAPC (1990-2019)
	DALYs	Age-standardized DALY rate per 10000	DALYs	Age-standardized DALY rate per 10000	Age-standardized DALY rate
	No.(95%UI)	No.(95%UI)	No.(95%UI)	No.(95%UI)	No.(95%CI)
**Global**	3705928.80(2953522.25,4482819.84)	91.16(73.29,109.79)	3812627.31(2992994.55,4633352.49)	45.82(36.10,55.58)	-2.42(-2.66, -2.18)
**Sex**	-	-	-	-	-
female	393959.54(298894.17,492584.04)	18.46(14.02,23.09)	328228.31(244488.86,418171.94)	7.50(5.58,9.57)	-3.24(-3.36, -3.11)
male	3311969.26(2642038.14,4001757.18)	174.85(141.58,209.62)	3484399.00(2733387.87,4236592.00)	88.75(70.06,107.64)	-2.37(-2.62, -2.12)
**Sociodemographic index**	-	-	-	-	-
Low SDI	56589.35(37381.18,77121.15)	23.41(15.90,31.04)	82338.53(55840.07,112043.65)	15.64(10.89,20.87)	-1.38(-1.43, -1.33)
Low-middle SDI	326003.41(242268.66,410680.27)	52.49(40.01,65.06)	443043.17(330993.96,553665.24)	31.83(24.22,39.58)	-1.73(-1.92, -1.55)
Middle SDI	1103474.98(827593.60,1395792.89)	103.05(78.57,129.56)	1490284.44(1136537.38,1853713.91)	58.24(44.69,71.84)	-1.89(-2.29, -1.50)
High-middle SDI	1387754.77(1100634.17,1681926.27)	125.55(100.29,151.65)	1308851.69(1025981.77,1600278.55)	63.33(49.57,77.61)	-2.52(-2.77, -2.27)
High SDI	831164.74(692977.77,964314.85)	81.19(67.39,94.09)	487049.45(392800.93,579035.13)	26.85(21.68,31.95)	-3.94(-4.01, -3.87)
**Region**	-	-	-	-	-
Andean Latin America	11108.61(7346.23,15510.23)	54.96(36.80,75.57)	14341.25(8858.94,20800.68)	25.82(16.09,37.32)	-2.54(-2.62, -2.46)

Australasia	8264.07(6633.45,9850.24)	35.23(28.23,42.10)	5247.11(4012.66,6457.70)	11.41(8.72,14.24)	-3.96(-4.11, -3.81)
Caribbean	8908.39(6893.23,11011.87)	34.23(26.50,42.21)	11236.54(8314.70,14426.37)	21.64(16.00,27.73)	-1.37(-1.43, -1.30)
Central Asia	62892.18(50419.81,75322.26)	126.48(101.99,151.91)	49714.00(39040.42,60945.27)	62.08(49.40,75.84)	-2.41(-2.61, -2.20)
Central Europe	153667.62(124164.29,180432.86)	101.71(82.49,119.14)	96338.95(75145.82,118648.73)	46.91(36.38,58.11)	-2.77(-2.84, -2.70)
Central Latin America	49343.97(35404.43,63459.76)	59.31(43.48,75.45)	48812.51(31227.10,68825.54)	20.64(13.36,28.75)	-3.80(-3.88, -3.72)
Central sub-Saharan Africa	5426.71(3090.80,8130.74)	22.34(13.02,32.73)	6771.57(3854.95,10488.87)	11.88(7.16,17.85)	-2.38(-2.49, -2.28)
East Asia	1510267.93(1110452.81,1944172.19)	164.34(122.73,209.45)	2156982.57(1608531.89,2751862.08)	99.67(74.49,126.59)	-1.66(-2.15, -1.16)
Eastern Europe	412624.39(329270.77,493551.24)	143.64(114.63,172.48)	224116.16(174682.88,277744.25)	66.51(52.01,81.89)	-3.24(-3.51, -2.97)
Eastern sub-Saharan Africa	13858.36(7617.78,20184.42)	18.14(10.28,26.09)	18088.85(10012.88,26724.48)	10.98(6.41,15.75)	-1.87(-2.02, -1.72)
High-income Asia Pacific	414364.62(341529.26,481325.66)	200.35(165.85,233.06)	220486.03(175425.77,263432.97)	52.95(42.13,63.50)	-4.67(-4.80, -4.55)
High-income North America	114127.89(95415.10,133223.50)	33.40(27.92,38.98)	85522.07(69495.01,102692.98)	14.21(11.55,16.97)	-3.05(-3.14, -2.95)
North Africa and middle East	104808.22(83391.66,127099.82)	57.97(45.87,70.19)	147893.38(116950.48,181273.18)	33.43(26.61,40.67)	-1.75(-2.04, -1.45)
Oceania	1595.84(977.26,2278.08)	49.19(31.36,68.01)	2889.20(1558.28,4566.67)	36.95(21.85,55.51)	-0.81(-0.90, -0.72)
South Asia	177675.93(124165.91,232327.02)	30.82(21.97,39.47)	222290.90(149831.15,299787.85)	15.56(10.70,20.81)	-2.52(-2.63, -2.41)
Southeast Asia	116535.40(84261.97,150346.92)	44.30(32.80,55.95)	142675.24(103268.08,184184.21)	22.74(16.81,29.01)	-2.53(-2.62, -2.44)
Southern Latin America	41139.37(32422.67,49847.41)	87.40(68.98,105.98)	35985.71(27611.94,44133.92)	43.84(33.66,53.91)	-2.30(-2.34, -2.25)
Southern sub-Saharan Africa	10569.04(7691.22,13481.56)	37.01(27.30,46.83)	10013.30(7128.50,13005.70)	17.22(12.48,22.04)	-2.99(-3.45, -2.53)

Tropical Latin America	95118.73(75784.72,114507.70)	102.21(81.94,123.04)	78313.08(59968.20,96770.17)	31.93(24.55,39.37)	-4.03(-4.24, -3.82)
Western Europe	380849.31(313679.95,446315.62)	67.35(55.59,79.14)	215497.53(173103.23,256412.09)	25.76(20.58,30.72)	-3.45(-3.55, -3.35)
Western sub-Saharan Africa	12782.22(7993.00,18216.10)	14.31(9.15,20.16)	19411.38(12174.21,27853.57)	10.32(6.70,14.50)	-0.92(-1.00, -0.85)

Note: DALY, age-standardized disability-adjusted life year; EAPC, estimated annual percentage change; UI is the uncertainty interval,which reflects the certainty of an estimate based on data availability, study size, and consistency across data sources.

Supplementary table 3. The top three and the bottom three regions of gastric cancer attributable to smoking ASDR, or DALY.

Measure	sex	Top three regions			Bottom three regions		
2019 ASR (per 100,000 people) ASDR							
	both	Central Asia(2.58)	Eastern Europe(2.60)	East Asia(4.65)	Western sub-Saharan Africa(0.48)	Central sub-Saharan Africa(0.50)	Eastern Sub-Saharan Africa(0.50)
	female	Tropical Latin America(0.66)	Central Europe(0.76)	Southern Latin America(0.93)	Central sub-Saharan Africa(0.07)	Western sub-Saharan Africa(0.08)	Eastern Sub-Saharan Africa(0.14)
	male	Central Asia(5.99)	Eastern Europe(6.22)	East Asia(9.68)	Australasia(0.72)	Eastern Sub-Saharan Africa(0.91)	Western sub-Saharan Africa(0.92)
Age-standardized DALY rate							
	both	Central Asia(62.08)	Eastern Europe(66.51)	East Asia(99.67)	Western sub-Saharan Africa(10.32)	Eastern Sub-Saharan Africa(10.98)	Australasia(11.41)
	female	Oceania(14.39)	Central Europe(17.95)	Southern Latin America(20.61)	Central sub-Saharan Africa(1.56)	Western sub-Saharan Africa(1.61)	Eastern Sub-Saharan Africa(2.72)
	male	Central Asia(137.29)	Eastern Europe(147.07)	East Asia(200.36)	Australasia(16.45)	Western sub-Saharan Africa(19.82)	Eastern Sub-Saharan Africa(20.04)

1990-2019 increase times							
Death (cases)							
	both	Western sub-Saharan Africa(1.53)	East Asia(1.60)	Oceania(1.78)	Eastern Europe(0.59)	Western Europe(0.62)	Australasia(0.66)
	female	Eastern Sub-Saharan Africa(1.78)	Oceania(1.87)	South Asia(1.87)	High-income Asia Pacific(0.61)	Western Europe(0.64)	Eastern Europe(0.70)
	male	North Africa and Middle East(1.53)	East Asia(1.60)	Oceania(1.76)	Eastern Europe(0.58)	Western Europe(0.61)	Australasia(0.63)
DALY (cases)							
	both	East Asia(1.43)	Western sub-Saharan Africa(1.52)	Oceania(1.81)	High-income Asia Pacific(0.53)	Eastern Europe(0.54)	Western Europe(0.57)
	female	South Asia(1.60)	Eastern Sub-Saharan Africa(1.68)	Oceania(1.84)	High-income Asia Pacific(0.46)	Western Europe(0.60)	Central Europe(0.66)
	male	East Asia(1.43)	Western sub-Saharan Africa(1.51)	Oceania(1.80)	Eastern Europe(0.53)	High-income Asia Pacific(0.54)	Western Europe(0.56)
EAPC							
Death							
	both	Caribbean(-1.34)	Oceania(-0.87)	Western sub-Saharan Africa(-0.80)	High-income Asia Pacific(-4.33)	Australasia(-4.10)	Tropical Latin America(-3.98)

	female	Eastern Sub-Saharan Africa(-0.69)	Western sub-Saharan Africa(-0.66)	Oceania(-0.46)	High-income Asia Pacific(-4.92)	Central Latin America(-4.20)	Tropical Latin America(-4.06)
	male	Caribbean(-1.40)	Oceania(-1.00)	Western sub-Saharan Africa(-0.76)	Australasia(-4.44)	High-income Asia Pacific(-4.43)	Tropical Latin America(-3.84)
DALY	both	Caribbean(-1.37)	Western sub-Saharan Africa(-0.92)	Oceania(-0.81)	High-income Asia Pacific(-4.67)	Tropical Latin America(-4.03)	Australasia(-3.96)
	female	Eastern Sub-Saharan Africa(-0.86)	Western sub-Saharan Africa(-0.81)	Oceania(-0.52)	High-income Asia Pacific(-4.97)	Central Latin America(-4.21)	Tropical Latin America(-3.93)
	male	Caribbean(-1.39)	Oceania(-0.91)	Western sub-Saharan Africa(-0.74)	High-income Asia Pacific(-4.80)	Australasia(-4.21)	Tropical Latin America(-3.97)

Note: ASR, age-standardized rate; ASDR, age-standardized death rate; DALY, age-standardized disability-adjusted life year; EAPC, estimated annual percentage change.

Supplementary table 4. The top three and the bottom three countries of gastric cancer attributable to smoking ASDR, or DALY.

Measure	sex	Top three countries			Bottom three countries		
2019 ASR (per 100,000 people)							
ASDR							
	both	Azerbaijan(4.29)	China(4.72)	Mongolia(6.84)	Nigeria(0.13)	Ethiopia(0.18)	Ghana(0.26)
	female	Greenland(1.40)	Kiribati(1.41)	North Macedonia(1.42)	Eritrea(0.02)	Ethiopia(0.02)	Libya(0.02)
	male	China(9.83)	Azerbaijan(9.89)	Mongolia(15.57)	Nigeria(0.24)	Ethiopia(0.32)	Ghana(0.49)
Age-standardized DALY rate							
	both	China(100.89)	Democratic People's Republic of Korea(102.69)	Mongolia(154.53)	Nigeria(2.59)	Ethiopia(3.69)	Ghana(5.04)
	female	Greenland(30.64)	North Macedonia(32.64)	Nauru(32.74)	Eritrea(0.45)	Ethiopia(0.49)	Libya(0.53)
	male	Azerbaijan(214.74)	Democratic People's Republic of Korea(226.80)	Mongolia(335.42)	Nigeria(4.84)	Ethiopia(6.72)	Ghana(9.36)
1990-2019 increase times							
Death (cases)							
	both	Qatar(3.45)	Djibouti(4.00)	United Arab Emirates(5.23)	Norway(0.36)	Sweden(0.45)	Latvia(0.46)
	female	United Arab Emirates(3.15)	Niger(3.38)	Djibouti(3.89)	Norway(0.39)	Iceland(0.43)	Belarus(0.44)

	male	Qatar(3.48)	Djibouti(4.01)	United Arab Emirates(5.40)	Norway(0.34)	Sweden(0.41)	Latvia(0.46)
DALY (cases)							
	both	Qatar(3.38)	Djibouti(3.83)	United Arab Emirates(5.83)	Norway(0.36)	Latvia(0.40)	Sweden(0.42)
	female	Afghanistan(3.40)	Djibouti(3.58)	United Arab Emirates(3.78)	Norway(0.38)	Iceland(0.39)	Croatia(0.40)
	male	Qatar(3.40)	Djibouti(3.85)	United Arab Emirates(5.99)	Norway(0.35)	Sweden(0.38)	Latvia(0.40)
EAPC Death							
	both	Sao Tome and Principe(1.05)	Afghanistan(1.09)	Dominican Republic(1.19)	Singapore(-6.35)	Republic of Korea(-5.82)	Colombia(-5.00)
	female	Sao Tome and Principe(0.94)	Lesotho(1.12)	Afghanistan(1.92)	Republic of Korea(-6.01)	Colombia(-5.61)	Iceland(-5.40)
	male	Sao Tome and Principe(1.02)	Afghanistan(1.43)	Dominican Republic(1.44)	Singapore(-6.58)	Republic of Korea(-5.98)	Norway(-5.06)
DALY							
	both	Sao Tome and Principe(1.03)	Dominican Republic(1.09)	Afghanistan(1.21)	Singapore(-6.72)	Republic of Korea(-6.43)	Ethiopia(-5.12)
	female	Sao Tome and Principe(0.90)	Lesotho(1.18)	Afghanistan(1.97)	Republic of Korea(-6.82)	Iceland(-5.66)	Maldives(-5.63)
	male	Sao Tome and Principe(1.04)	Dominican Republic(1.35)	Afghanistan(1.51)	Singapore(-7.00)	Republic of Korea(-6.59)	Ethiopia(-5.08)

Note: ASR, age-standardized rate; ASDR, age-standardized death rate; DALY, age-standardized disability-adjusted life year; EAPC, estimated annual

percentage change.

Supplementary table 5. The death cases and age-standardized death rate of gastric cancer attributable to smoking in 1990 and 2019, and its temporal trends from 1990 to 2019.

Nation	Sex	Death Cases No. (95% UI)		Change in absolute number (%)	ASDR per 100,000 No.(95% UI)		1990-2019 EAPC No. (95%CI)
		1990	2019		1990	2019	
Afghanistan	both	113.93(72.11,160.92)	251.38(162.30,351.52)	1.206350611	1.71(1.11,2.39)	2.27(1.51,3.08)	1.09(0.98,1.21)
Albania	both	67.93(54.99,81.74)	110.14(76.77,151.63)	0.621451036	1664.13(1345.23,2015.29)	2.51(1.76,3.47)	-1.11(-1.37,-0.85)
Algeria	both	167.10(120.16,219.29)	242.42(177.29,324.13)	0.45074596	3703.26(2657.68,4903.54)	0.86(0.64,1.15)	-2.53(-2.71,-2.36)
American Samoa	both	0.67(0.47,0.88)	0.98(0.67,1.31)	0.466834636	17.97(12.00,24.49)	2.12(1.48,2.83)	-1.40(-1.55,-1.24)
Andorra	both	1.45(1.02,2.02)	2.10(1.51,2.81)	0.448270599	32.48(22.39,45.76)	1.47(1.06,1.99)	-2.26(-2.37,-2.15)
Angola	both	50.54(31.37,72.39)	78.13(51.33,109.49)	0.545853329	1394.34(838.44,2034.82)	0.77(0.53,1.06)	-2.40(-2.55,-2.24)
Antigua and Barbuda	both	0.81(0.61,1.04)	1.00(0.72,1.33)	0.227495745	16.60(12.40,21.31)	1.04(0.75,1.39)	-1.27(-1.51,-1.03)
Argentina	both	1053.72(839.54,1264.05)	930.29(729.68,1127.43)	-0.117141713	24277.13(19162.73,29194.42)	1.71(1.34,2.07)	-2.13(-2.22,-2.04)
Armenia	both	145.09(120.18,168.6	114.21(88.81,139.92)	-0.21279	4082.50(3357.19,4780.05	2.74(2.13,3.3	-2.39(-2.58,-2

		1)		5349	)	7)	.19)
Australia	both	298.11(240.69,358.09)	194.82(150.04,241.77)	-0.346498842	6521.72(5236.03,7809.55)	0.47(0.36,0.58)	-4.14(-4.30,-3.97)
Austria	both	389.15(313.58,465.50)	190.50(151.12,231.47)	-0.510472736	8143.05(6593.14,9725.59)	1.04(0.83,1.27)	-4.14(-4.35,-3.92)
Azerbaijan	both	289.40(227.27,350.89)	373.20(273.46,494.69)	0.289590189	8217.46(6348.02,10049.47)	4.29(3.23,5.82)	-0.89(-1.14,-0.64)
Bahrain	both	3.49(2.64,4.44)	5.59(3.96,7.61)	0.598265214	84.24(62.44,107.78)	0.89(0.66,1.18)	-4.05(-4.39,-3.71)
Bangladesh	both	959.90(686.15,1250.80)	1087.03(740.79,1531.46)	0.132442036	23663.90(16190.97,31446.08)	0.89(0.61,1.24)	-3.19(-3.48,-2.90)
Barbados	both	4.55(3.45,5.77)	3.74(2.71,4.95)	-0.178112985	89.63(66.83,113.71)	0.75(0.55,0.99)	-2.82(-3.04,-2.61)
Belarus	both	861.48(694.28,1020.49)	447.51(327.62,587.33)	-0.480529142	23008.98(18142.61,27795.37)	2.80(2.06,3.71)	-3.66(-3.94,-3.39)
Belgium	both	423.21(339.60,506.41)	238.13(189.44,289.23)	-0.437335767	8028.42(6555.12,9531.76)	0.99(0.80,1.20)	-3.32(-3.49,-3.15)
Belize	both	1.37(1.01,1.76)	2.79(2.00,3.66)	1.032473092	29.60(21.50,38.26)	1.09(0.79,1.42)	-1.64(-2.20,-1.08)
Benin	both	26.33(17.05,36.66)	31.18(19.78,46.34)	0.184417049	575.37(365.77,822.25)	0.73(0.47,1.07)	-1.81(-2.02,-1.61)
Bermuda	both	0.92(0.66,1.22)	0.82(0.59,1.09)	-0.115027173	20.30(13.98,26.90)	0.61(0.43,0.81)	-2.99(-3.19,-2.79)
Bhutan	both	1.73(1.06,2.50)	2.98(1.91,4.33)	0.725154465	45.52(26.74,67.26)	0.58(0.38,0.84)	-0.89(-0.97,-0.82)
Bolivia (Plurinational	both	159.37(105.88,222.8	202.18(129.42,291.51	0.268614	3705.14(2413.60,5327.52	2.44(1.59,3.4	-2.94(-3.29,-2

State of)		4)	)	481	)	8)	.60)
Bosnia and Herzegovina	both	116.77(93.07,140.60)	158.04(116.55,209.45)	0.353501155	3031.97(2399.47,3650.16)	2.62(1.94,3.45)	-0.52(-0.75,-0.28)
Botswana	both	7.86(4.77,11.58)	12.56(8.22,17.74)	0.599393205	197.68(114.75,298.77)	1.04(0.71,1.43)	-2.08(-2.63,-1.54)
Brazil	both	3923.14(3135.64,4708.62)	3464.75(2688.45,4240.03)	-0.116841524	94013.89(74870.15,113021.59)	1.49(1.16,1.82)	-4.01(-4.21,-3.82)
Brunei Darussalam	both	6.14(4.81,7.67)	4.91(3.72,6.33)	-0.201306832	156.79(122.68,194.66)	2.00(1.49,2.57)	-4.47(-4.67,-4.28)
Bulgaria	both	608.49(496.27,717.42)	354.14(261.25,462.05)	-0.418011864	15388.25(12427.87,18133.42)	2.49(1.83,3.24)	-1.26(-1.68,-0.85)
Burkina Faso	both	36.51(19.22,56.87)	47.46(25.87,73.78)	0.299869173	926.15(473.18,1456.10)	0.56(0.32,0.85)	-1.30(-1.55,-1.05)
Burundi	both	30.19(17.54,44.93)	23.98(12.60,37.49)	-0.205723541	767.15(426.62,1173.05)	0.57(0.32,0.86)	-3.67(-3.93,-3.42)
Cambodia	both	132.93(96.28,180.02)	187.90(138.08,238.31)	0.413597551	3317.32(2355.48,4526.91)	1.81(1.35,2.27)	-1.98(-2.06,-1.91)
Cameroon	both	48.31(31.09,69.08)	84.50(50.26,128.63)	0.749153616	1239.38(774.15,1809.74)	0.78(0.48,1.16)	-1.17(-1.33,-1.02)
Canada	both	668.40(548.40,793.35)	551.93(429.89,676.33)	-0.174252733	14080.49(11536.90,16438.73)	0.77(0.61,0.94)	-3.41(-3.51,-3.30)
Cabo Verde	both	4.87(3.17,6.56)	5.83(4.02,7.92)	0.196233112	104.67(67.06,143.23)	1.44(0.99,1.97)	-2.59(-3.13,-2.06)
Central African Republic	both	14.10(8.50,20.99)	15.39(8.97,23.53)	0.090870899	402.90(233.21,611.54)	0.71(0.44,1.03)	-2.08(-2.24,-1.93)
Chad	both	30.66(18.30,44.86)	49.15(29.36,73.61)	0.603242	693.07(396.28,1036.82)	1.01(0.62,1.4	-0.15(-0.32,0.

				189		8)	02)
Chile	both	551.56(416.99,690.50)	594.45(439.39,748.17)	0.077767788	13802.08(10131.48,17413.51)	2.46(1.82,3.10)	-2.76(-2.87,-2.66)
China	both	58782.53(44303.37,74440.84)	93853.88(71027.63,118336.58)	0.596628803	1476331.93(1085512.29,1905742.56)	4.72(3.58,5.92)	-1.44(-1.93,-0.95)
Colombia	both	701.16(520.73,890.12)	536.40(341.50,772.24)	-0.234980951	17093.83(12311.51,22125.62)	1.02(0.65,1.46)	-5.00(-5.16,-4.83)
Comoros	both	1.97(1.00,3.01)	2.51(1.51,3.70)	0.269637468	45.28(20.40,71.66)	0.56(0.35,0.82)	-2.01(-2.14,-1.88)
Congo	both	10.27(6.37,14.64)	13.19(8.43,19.28)	0.283354714	272.76(161.93,401.51)	0.57(0.39,0.79)	-2.46(-2.78,-2.15)
Costa Rica	both	108.89(84.81,132.44)	107.56(73.17,147.69)	-0.012232799	2395.15(1839.04,2958.00)	2.12(1.45,2.90)	-4.21(-4.51,-3.92)
C 么 te d'Ivoire	both	51.24(33.26,71.67)	104.36(68.98,146.24)	1.036582942	1409.21(890.83,2008.25)	1.10(0.75,1.50)	-0.67(-0.78,-0.57)
Croatia	both	387.41(315.82,461.80)	188.79(139.02,248.02)	-0.5126881	9180.47(7526.78,10850.99)	2.10(1.55,2.78)	-3.72(-3.92,-3.52)
Cuba	both	174.23(140.79,207.69)	218.55(160.50,283.63)	0.254323709	3721.45(2995.73,4476.72)	1.14(0.84,1.48)	-1.07(-1.17,-0.97)
Cyprus	both	13.79(10.71,17.14)	26.36(20.43,32.97)	0.91153965	299.97(231.27,374.51)	1.33(1.04,1.66)	-0.93(-1.14,-0.71)
Czechia	both	554.25(443.50,668.06)	266.88(195.29,344.30)	-0.51847353	12093.97(9688.71,14501.33)	1.23(0.90,1.59)	-4.08(-4.17,-3.99)
Democratic Republic of the Congo	both	121.25(62.16,191.15)	135.81(70.59,222.68)	0.120069742	3188.94(1570.75,5080.45)	0.40(0.22,0.64)	-2.63(-2.77,-2.49)
Denmark	both	237.12(195.94,278.7	117.63(93.72,142.11)	-0.50390	4639.70(3828.93,5455.33	0.98(0.78,1.1	-3.42(-3.60,-3

		2)		5407	)	7)	.23)
Djibouti	both	1.32(0.71,2.03)	5.27(2.96,8.09)	3.000785254	36.33(18.78,58.30)	1.02(0.63,1.50)	-0.62(-0.77,-0.47)
Dominica	both	2.04(1.49,2.62)	1.50(1.03,2.06)	-0.265437411	42.50(30.54,55.44)	1.64(1.12,2.26)	-1.81(-2.06,-1.57)
Dominican Republic	both	37.96(28.47,48.91)	117.14(81.39,163.48)	2.08559303	832.26(614.07,1076.61)	1.33(0.93,1.84)	1.19(0.95,1.42)
Ecuador	both	182.90(130.81,240.27)	261.96(174.09,375.30)	0.432276079	3953.95(2726.90,5326.93)	1.84(1.24,2.61)	-2.49(-2.68,-2.29)
Egypt	both	224.40(173.13,276.15)	508.13(354.29,721.03)	1.264376328	5528.64(4244.07,6881.60)	0.95(0.66,1.32)	0.28(0.14,0.41)
El Salvador	both	37.71(27.36,49.68)	61.41(40.21,88.74)	0.628434904	924.85(647.49,1247.14)	1.04(0.68,1.50)	-1.25(-1.52,-0.98)
Equatorial Guinea	both	2.01(1.05,3.26)	1.68(0.91,2.75)	-0.162147941	55.19(27.17,90.57)	0.39(0.22,0.62)	-3.80(-4.32,-3.27)
Eritrea	both	7.62(4.04,12.28)	12.27(7.04,19.10)	0.611428658	241.46(122.16,392.36)	0.43(0.26,0.64)	-1.93(-2.17,-1.70)
Estonia	both	109.19(86.32,132.55)	57.28(40.86,75.77)	-0.475392405	2792.44(2203.28,3380.85)	2.23(1.58,2.98)	-3.14(-3.36,-2.91)
Ethiopia	both	107.90(55.20,166.22)	64.42(35.13,100.63)	-0.402982845	2910.55(1398.25,4515.61)	0.18(0.10,0.27)	-4.59(-4.85,-4.32)
Micronesia (Federated States of)	both	1.59(0.99,2.33)	1.82(1.05,2.67)	0.144139916	43.75(26.44,65.87)	2.67(1.68,3.78)	-0.98(-1.13,-0.84)
Fiji	both	4.68(3.12,6.49)	5.60(3.35,8.24)	0.197309323	126.85(80.65,182.61)	0.81(0.52,1.15)	-1.92(-2.10,-1.74)
Finland	both	175.36(139.91,212.7	88.56(67.41,110.42)	-0.49500	3866.91(3077.61,4643.80	0.71(0.55,0.8	-4.24(-4.49,-3

		2)		8629	)	8)	.98)
France	both	1750.52(1400.54,2077.94)	1187.27(930.29,1463.17)	-0.32176129	34434.22(27602.27,40867.40)	0.84(0.66,1.03)	-3.24(-3.37,-3.10)
Gabon	both	4.33(2.58,6.42)	5.11(3.07,7.61)	0.178240114	112.59(64.09,171.72)	0.51(0.32,0.74)	-1.44(-1.50,-1.38)
Georgia	both	211.68(166.52,259.82)	149.05(112.59,188.33)	-0.295861526	5932.18(4610.76,7287.79)	2.54(1.91,3.20)	-0.02(-0.50,0.46)
Germany	both	3777.14(3073.71,4479.45)	2541.12(2016.96,3077.76)	-0.327236593	77613.53(63357.41,92084.22)	1.29(1.03,1.56)	-2.87(-3.05,-2.68)
Ghana	both	30.59(18.45,46.48)	34.32(20.44,50.38)	0.122102425	722.47(406.63,1127.89)	0.26(0.16,0.37)	-3.20(-3.57,-2.82)
Greece	both	527.60(430.71,616.25)	501.53(408.48,598.32)	-0.049401998	11210.14(9211.20,13091.23)	2.06(1.69,2.43)	-2.18(-2.35,-2.02)
Greenland	both	1.72(1.31,2.15)	1.80(1.31,2.36)	0.044197008	47.76(35.44,60.87)	2.68(1.97,3.46)	-2.57(-2.72,-2.41)
Grenada	both	1.05(0.74,1.38)	0.93(0.65,1.23)	-0.117140628	21.87(15.61,29.22)	0.84(0.60,1.12)	-1.62(-1.82,-1.42)
Guam	both	0.72(0.49,0.98)	1.22(0.82,1.71)	0.690044342	19.19(12.55,26.91)	0.64(0.44,0.90)	-1.66(-2.01,-1.30)
Guatemala	both	77.85(48.67,109.41)	170.10(103.33,248.93)	1.185038136	1843.79(1090.22,2667.10)	1.63(1.02,2.36)	-1.83(-2.38,-1.27)
Guinea	both	37.34(22.98,53.08)	73.93(46.39,107.76)	0.979889262	887.37(527.58,1295.25)	1.43(0.91,2.07)	0.90(0.71,1.10)
Guinea-Bissau	both	5.17(3.09,7.64)	4.39(2.69,6.50)	-0.150473046	135.12(78.94,203.01)	0.66(0.42,0.97)	-2.06(-2.54,-1.58)
Guyana	both	5.30(3.79,7.03)	4.44(2.94,6.37)	-0.16319	134.47(93.99,181.49)	0.73(0.49,1.0	-2.43(-2.57,-2

				5239		3)	.30)
Haiti	both	51.39(28.00,77.72)	53.42(28.03,85.43)	0.039571816	1374.19(731.67,2120.77)	0.83(0.45,1.30)	-2.29(-2.34,-2.24)
Honduras	both	32.87(23.39,43.66)	87.28(60.64,119.42)	1.655030397	792.47(546.02,1088.20)	1.56(1.10,2.11)	0.05(-0.22,0.31)
Hungary	both	687.14(548.17,807.64)	357.30(269.68,455.49)	-0.480016438	15661.76(12600.61,18395.16)	1.83(1.38,2.33)	-3.20(-3.29,-3.12)
Iceland	both	9.38(7.58,11.27)	4.77(3.69,6.01)	-0.490970524	189.80(153.73,226.76)	0.84(0.65,1.04)	-4.72(-4.82,-4.62)
India	both	5120.35(3599.51,6646.22)	7152.67(4961.48,9579.02)	0.396908958	134726.21(92009.62,178907.19)	0.67(0.47,0.88)	-2.62(-2.78,-2.46)
Indonesia	both	1057.99(736.14,1431.03)	2021.64(1397.04,2676.39)	0.910836158	27243.38(18213.56,37689.57)	1.08(0.77,1.40)	-0.12(-0.26,0.03)
Iran (Islamic Republic of)	both	903.70(684.69,1119.19)	1457.84(1156.33,1764.39)	0.61318596	23116.11(17479.69,28550.99)	2.15(1.71,2.62)	-1.71(-2.01,-1.41)
Iraq	both	106.46(77.47,138.34)	214.33(156.14,276.08)	1.013224588	2604.71(1854.35,3476.90)	1.05(0.78,1.33)	-1.37(-1.48,-1.26)
Ireland	both	145.16(117.66,172.05)	85.04(66.67,104.85)	-0.414178141	2836.45(2313.38,3369.08)	1.10(0.87,1.36)	-4.02(-4.10,-3.95)
Israel	both	117.90(95.48,141.67)	117.90(92.41,143.60)	4.38124E-06	2408.88(1972.93,2900.56)	1.00(0.78,1.21)	-3.58(-3.76,-3.40)
Italy	both	3740.93(3032.80,4430.13)	2244.78(1771.95,2729.62)	-0.399941764	76734.72(62066.51,91238.83)	1.49(1.19,1.80)	-3.76(-3.89,-3.63)
Jamaica	both	35.87(27.18,44.45)	36.09(25.43,48.62)	0.006155469	755.41(578.67,930.07)	1.22(0.86,1.65)	-1.22(-1.63,-0.80)
Japan	both	13930.05(11525.12,	9982.81(7835.62,121	-0.28336	309343.36(255085.58,35	2.60(2.08,3.1	-3.94(-4.02,-3

		16212.83)	52.59)	1644	9482.07)	2)	.86)
Jordan	both	22.10(16.91,27.80)	59.34(43.98,77.01)	1.685448699	569.33(433.65,720.67)	1.06(0.79,1.37)	-2.28(-2.43,-2.14)
Kazakhstan	both	751.00(589.25,923.98)	422.43(326.33,535.74)	-0.437505861	21144.07(16380.89,26240.81)	2.41(1.84,3.04)	-2.82(-2.99,-2.66)
Kenya	both	51.71(31.38,75.74)	126.35(77.48,181.88)	1.443234129	1233.99(710.87,1872.67)	0.64(0.41,0.92)	-0.01(-0.54,0.52)
Kiribati	both	1.49(1.01,2.01)	2.34(1.55,3.27)	0.565083741	42.62(27.97,58.89)	3.75(2.62,4.98)	-0.36(-0.41,-0.30)
Kuwait	both	5.59(4.33,6.93)	11.97(8.94,15.65)	1.140372066	143.87(111.17,178.86)	0.59(0.44,0.77)	-2.23(-2.64,-1.82)
Kyrgyzstan	both	203.06(164.81,239.84)	154.36(120.36,192.07)	-0.239796654	5814.48(4671.17,6937.05)	3.37(2.66,4.17)	-2.40(-2.88,-1.92)
Lao People's Democratic Republic	both	64.34(44.74,85.62)	52.31(36.43,70.15)	-0.186973021	1671.74(1135.40,2264.31)	1.39(0.99,1.84)	-3.18(-3.37,-2.98)
Latvia	both	181.64(147.05,217.25)	83.65(62.27,107.66)	-0.539442843	4728.82(3742.71,5729.78)	2.18(1.61,2.80)	-3.06(-3.37,-2.74)
Lebanon	both	44.15(33.16,57.62)	84.97(63.65,116.14)	0.924317919	1053.70(785.83,1396.71)	1.63(1.22,2.23)	-0.42(-0.62,-0.21)
Lesotho	both	13.71(9.27,18.96)	18.42(13.17,24.91)	0.343702668	341.18(224.08,475.50)	1.53(1.11,2.04)	0.24(0.15,0.33)
Liberia	both	12.23(7.37,18.21)	10.83(6.01,16.65)	-0.114328807	280.84(163.51,423.16)	0.62(0.36,0.93)	-1.75(-2.06,-1.44)
Libya	both	22.20(15.51,29.29)	41.36(29.40,55.24)	0.863359796	541.01(375.60,728.66)	0.89(0.63,1.18)	-1.54(-1.81,-1.27)
Lithuania	both	201.25(159.34,244.1	108.09(80.16,140.45)	-0.46289	5104.07(3936.45,6278.15	1.92(1.41,2.5	-2.83(-3.05,-2

		5)		6068	)	2)	.61)
Luxembourg	both	14.86(11.61,18.43)	9.41(7.14,11.97)	-0.36705 0678	311.22(241.91,382.74)	0.91(0.69,1.1 6)	-3.79(-3.86,-3 .72)
North Macedonia	both	106.10(82.03,130.41)	124.72(91.15,165.81)	0.175573 124	2695.89(2084.51,3340.64)	3.79(2.79,4.9 9)	-1.74(-1.90,-1 .59)
Madagascar	both	38.29(21.16,57.82)	33.72(16.41,55.18)	-0.11917 1028	981.49(517.46,1515.39)	0.34(0.18,0.5 2)	-3.39(-3.68,-3 .10)
Malawi	both	14.33(8.56,20.48)	18.72(11.12,27.23)	0.306129 766	314.59(179.70,460.43)	0.31(0.19,0.4 4)	-1.90(-2.13,-1 .68)
Malaysia	both	135.38(103.29,167.0 6)	227.37(164.21,304.48)	0.679506 548	3210.39(2417.70,4036.02)	0.95(0.69,1.2 6)	-2.33(-2.58,-2 .09)
Maldives	both	1.27(0.89,1.66)	1.56(1.17,2.01)	0.230829 256	31.42(21.56,41.57)	0.62(0.46,0.7 9)	-4.50(-4.73,-4 .26)
Mali	both	36.92(20.25,56.94)	82.53(45.04,127.26)	1.235111 548	953.99(487.04,1516.83)	1.06(0.61,1.5 9)	0.59(0.47,0.7 1)
Malta	both	11.02(8.77,13.39)	8.29(6.44,10.29)	-0.24795 5449	241.40(192.91,287.79)	0.87(0.68,1.0 7)	-3.87(-3.97,-3 .77)
Marshall Islands	both	0.39(0.27,0.55)	0.58(0.36,0.84)	0.479824 316	11.04(7.28,15.71)	1.79(1.20,2.5 0)	-0.90(-1.08,-0 .72)
Mauritania	both	11.78(7.39,16.73)	13.23(8.28,19.78)	0.122807 485	299.39(179.43,427.38)	0.69(0.44,1.0 2)	-1.78(-1.94,-1 .62)
Mauritius	both	15.28(11.36,19.23)	14.06(9.63,18.76)	-0.07982 4225	365.48(263.79,470.02)	0.82(0.57,1.0 9)	-3.69(-3.89,-3 .49)
Mexico	both	828.03(599.33,1059. 98)	843.28(550.69,1174.6 7)	0.018417 859	17780.10(12230.48,2357 5.94)	0.76(0.50,1.0 4)	-3.73(-3.80,-3 .67)
Republic of Moldova	both	164.88(129.61,197.4	109.77(85.85,137.56)	-0.33420	4720.29(3713.33,5695.52	1.87(1.47,2.3	-1.52(-1.99,-1

		9)		9417	)	3)	.05)
Mongolia	both	94.30(65.78,126.33)	144.49(97.41,204.72)	0.532178851	2375.73(1627.80,3269.16)	6.84(4.82,9.35)	-1.75(-2.00,-1.49)
Montenegro	both	13.62(10.60,16.80)	20.85(15.74,26.07)	0.530920337	327.24(251.98,409.00)	2.06(1.56,2.57)	-0.19(-0.42,0.04)
Morocco	both	96.07(67.65,125.61)	150.14(103.13,200.44)	0.562798628	2324.52(1631.01,3041.29)	0.53(0.37,0.70)	-1.55(-1.82,-1.28)
Mozambique	both	30.57(16.77,45.59)	50.68(27.43,78.03)	0.657532954	720.00(380.45,1095.06)	0.53(0.30,0.79)	-0.11(-0.22,0.00)
Myanmar	both	711.90(490.83,981.02)	455.90(319.04,614.10)	-0.359596979	17920.82(11914.98,25255.09)	1.11(0.79,1.47)	-3.96(-4.07,-3.84)
Namibia	both	3.75(2.35,5.38)	4.50(2.75,6.53)	0.201126538	79.12(46.75,118.46)	0.38(0.24,0.55)	-1.61(-1.78,-1.43)
Nepal	both	131.72(76.39,191.54)	231.35(135.95,336.27)	0.756393412	3259.30(1797.21,4872.25)	1.19(0.73,1.67)	-0.72(-0.87,-0.57)
Netherlands	both	815.57(673.76,954.56)	520.91(407.98,636.42)	-0.361296512	16293.48(13476.30,18871.86)	1.44(1.14,1.76)	-3.69(-3.87,-3.51)
New Zealand	both	82.94(66.54,99.24)	56.31(43.43,70.35)	-0.321100108	1742.35(1385.71,2077.13)	0.71(0.54,0.88)	-3.90(-4.00,-3.80)
Nicaragua	both	28.39(20.45,36.73)	51.85(34.89,71.90)	0.826449668	670.09(462.12,900.93)	1.28(0.87,1.75)	-2.07(-2.30,-1.84)
Niger	both	10.60(6.15,16.00)	28.32(16.00,44.74)	1.670647279	282.43(151.21,437.89)	0.44(0.26,0.67)	0.58(0.43,0.73)
Nigeria	both	77.55(44.83,117.04)	94.92(56.71,148.65)	0.223942208	1812.24(990.75,2790.04)	0.13(0.08,0.20)	-1.07(-1.29,-0.86)
Democratic People's	both	690.97(481.52,953.0	1316.12(966.49,1698.	0.904736	19539.27(13175.62,2765	4.05(2.99,5.2	-0.15(-0.40,0.

Republic of Korea		0)	68)	302	6.96)	3)	10)
Northern Mariana Islands	both	0.41(0.26,0.57)	0.71(0.47,0.98)	0.740914226	12.56(7.47,18.28)	1.46(1.01,1.95)	-2.13(-2.37,-1.88)
Norway	both	152.77(120.86,183.71)	54.72(42.40,68.03)	-0.641801812	3047.17(2434.14,3663.44)	0.56(0.44,0.70)	-4.57(-4.84,-4.29)
Oman	both	10.02(6.68,13.92)	8.08(5.78,10.71)	-0.193915538	271.58(177.63,381.74)	0.66(0.47,0.87)	-3.12(-3.28,-2.97)
Pakistan	both	734.86(533.06,957.48)	879.07(598.30,1206.31)	0.196237114	15981.00(11411.18,21187.29)	0.94(0.66,1.26)	-1.35(-1.67,-1.04)
Palestine	both	15.92(11.09,21.58)	23.93(17.56,30.65)	0.502950061	374.88(258.06,514.78)	1.14(0.84,1.44)	-1.90(-2.01,-1.78)
Panama	both	33.04(25.33,40.82)	34.69(23.66,48.34)	0.05018266	735.68(556.26,926.63)	0.84(0.57,1.17)	-3.50(-3.66,-3.34)
Papua New Guinea	both	31.55(17.66,47.05)	64.83(32.85,102.64)	1.054922558	891.45(482.32,1350.35)	1.45(0.80,2.21)	-0.48(-0.63,-0.32)
Paraguay	both	51.27(39.71,64.30)	82.16(56.83,113.53)	0.602354926	1104.85(836.91,1425.51)	1.56(1.08,2.16)	-2.08(-2.62,-1.54)
Peru	both	148.61(91.15,222.29)	228.08(128.12,353.89)	0.534803821	3449.51(1965.52,5338.33)	0.72(0.41,1.12)	-1.68(-1.93,-1.42)
Philippines	both	393.68(293.62,498.43)	573.83(414.76,785.80)	0.457591294	10256.61(7423.73,13220.93)	0.80(0.60,1.08)	-2.20(-2.36,-2.04)
Poland	both	2251.79(1821.66,2638.80)	1497.64(1137.37,1928.16)	-0.334911286	53322.65(43318.41,62875.55)	2.11(1.60,2.70)	-3.30(-3.42,-3.18)
Portugal	both	628.78(496.40,752.88)	393.18(307.24,477.37)	-0.374692044	14350.87(11351.88,17323.19)	1.71(1.33,2.07)	-3.43(-3.50,-3.36)
Puerto Rico	both	51.01(37.37,65.60)	38.77(26.05,54.13)	-0.23985	1017.93(737.88,1311.76)	0.51(0.34,0.7	-3.82(-3.97,-3

				7525		1)	.68)
Qatar	both	1.35(0.95,1.85)	4.66(3.08,6.70)	2.449562164	37.85(26.23,52.21)	1.02(0.71,1.43)	-1.72(-2.10,-1.33)
Romania	both	922.01(725.89,1107.61)	756.13(558.36,985.53)	-0.179913497	23924.26(18721.80,28949.65)	2.07(1.53,2.69)	-1.66(-1.85,-1.47)
Russian Federation	both	9541.97(7516.44,11302.45)	6179.26(4770.99,7713.50)	-0.352413049	258294.64(203582.82,309777.29)	2.60(2.01,3.25)	-2.69(-2.98,-2.40)
Rwanda	both	35.00(20.72,50.77)	43.94(27.43,62.93)	0.255414559	854.02(494.91,1261.26)	0.88(0.57,1.23)	-2.42(-2.79,-2.05)
Saint Lucia	both	2.12(1.60,2.68)	2.74(1.97,3.60)	0.290161331	47.80(35.40,60.58)	1.29(0.93,1.69)	-2.48(-2.77,-2.19)
Saint Vincent and the Grenadines	both	1.04(0.75,1.36)	1.28(0.89,1.72)	0.233648431	23.26(16.37,30.51)	0.96(0.67,1.29)	-1.63(-1.92,-1.34)
Samoa	both	2.77(2.02,3.56)	3.25(2.44,4.24)	0.172074184	68.41(48.02,90.66)	2.38(1.80,3.06)	-1.06(-1.10,-1.01)
Sao Tome and Principe	both	0.45(0.27,0.67)	0.83(0.52,1.21)	0.857567192	10.06(5.79,15.46)	0.89(0.56,1.28)	1.05(0.97,1.14)
Saudi Arabia	both	37.81(25.09,52.41)	70.19(49.68,94.84)	0.856295381	976.35(635.73,1388.91)	0.45(0.33,0.61)	-1.80(-1.90,-1.71)
Senegal	both	44.09(29.18,61.11)	58.61(38.00,83.41)	0.329352594	1095.46(702.31,1542.45)	0.84(0.56,1.18)	-1.50(-1.70,-1.29)
Serbia	both	331.55(251.64,415.02)	324.02(238.98,427.07)	-0.022718161	8526.28(6409.06,10812.24)	1.99(1.45,2.61)	-1.58(-1.81,-1.35)
Seychelles	both	1.11(0.82,1.42)	1.15(0.84,1.47)	0.037100165	25.41(18.47,33.27)	1.10(0.82,1.39)	-2.30(-2.50,-2.11)
Sierra Leone	both	35.99(22.00,52.04)	34.88(19.28,54.46)	-0.03084	830.70(478.80,1242.72)	1.07(0.61,1.6	-1.67(-1.97,-1

				1498		3)	.37)
Singapore	both	71.62(56.43,87.89)	44.36(33.98,55.42)	-0.380624677	1682.56(1320.46,2062.10)	0.59(0.46,0.75)	-6.35(-6.57,-6.13)
Slovakia	both	227.08(178.34,275.73)	130.78(91.66,174.03)	-0.424067982	5445.12(4249.08,6640.07)	1.40(0.98,1.85)	-3.37(-3.59,-3.15)
Slovenia	both	99.89(71.24,132.32)	71.77(52.83,96.29)	-0.281487546	2405.63(1689.20,3244.96)	1.66(1.23,2.23)	-3.44(-3.63,-3.25)
Solomon Islands	both	6.11(3.80,8.64)	10.72(6.80,15.30)	0.755170715	180.34(108.14,262.92)	3.63(2.50,5.04)	-0.98(-1.05,-0.91)
Somalia	both	24.01(12.94,38.84)	41.12(20.28,64.14)	0.712505084	672.32(335.27,1123.49)	0.66(0.35,1.02)	-1.65(-1.72,-1.58)
South Africa	both	313.45(234.07,399.94)	279.24(206.29,360.04)	-0.109132848	8133.20(5818.59,10545.04)	0.65(0.48,0.83)	-3.46(-4.02,-2.89)
Republic of Korea	both	3647.40(3004.79,4299.26)	2377.08(1880.72,2869.29)	-0.348280556	103181.92(83346.62,121807.44)	2.67(2.11,3.22)	-5.82(-6.07,-5.57)
South Sudan	both	18.15(9.55,28.36)	16.60(8.20,27.26)	-0.084973525	442.09(225.13,718.87)	0.50(0.26,0.80)	-1.83(-1.94,-1.72)
Spain	both	1951.59(1610.87,2284.33)	1390.97(1122.53,1674.43)	-0.287262121	42904.65(35281.25,50111.22)	1.47(1.19,1.76)	-3.02(-3.30,-2.73)
Sri Lanka	both	114.77(76.36,154.03)	108.71(64.85,164.53)	-0.052868809	2576.86(1635.24,3605.30)	0.45(0.27,0.66)	-3.07(-3.32,-2.82)
Sudan	both	213.52(135.02,291.87)	361.19(232.47,523.42)	0.691626375	5306.26(3332.99,7380.87)	2.16(1.39,3.10)	-0.29(-0.38,-0.19)
Suriname	both	3.77(2.84,4.85)	6.23(4.50,8.22)	0.652477873	90.46(65.79,120.12)	1.06(0.76,1.40)	-1.46(-1.81,-1.11)
Eswatini	both	2.21(1.29,3.35)	2.37(1.32,3.73)	0.072965	54.02(28.73,85.03)	0.47(0.28,0.7	-1.82(-2.21,-1

				39		2)	.43)
Sweden	both	320.80(256.46,383.58)	144.41(111.94,178.26)	-0.549831512	5936.71(4801.48,7057.51)	0.64(0.50,0.78)	-4.04(-4.21,-3.87)
Switzerland	both	244.21(196.44,292.40)	138.84(109.60,170.92)	-0.431496668	4940.98(4010.02,5884.82)	0.76(0.61,0.94)	-3.65(-3.95,-3.35)
Syrian Arab Republic	both	56.24(40.07,73.70)	89.90(62.72,123.85)	0.598423184	1402.24(991.72,1867.93)	0.83(0.60,1.13)	-1.52(-1.77,-1.26)
Taiwan (Province of China)	both	576.85(472.65,678.35)	648.54(479.22,861.55)	0.124262569	14396.72(11742.79,16937.94)	1.60(1.18,2.14)	-2.99(-3.12,-2.85)
Tajikistan	both	173.08(137.46,212.22)	136.91(99.05,182.84)	-0.208999991	4817.29(3780.94,5891.36)	2.99(2.19,3.97)	-1.77(-2.07,-1.47)
United Republic of Tanzania	both	128.28(74.61,190.82)	184.20(104.04,272.59)	0.435946592	3150.24(1711.80,4813.14)	0.83(0.49,1.21)	-1.56(-1.69,-1.43)
Thailand	both	771.08(594.46,973.01)	767.42(514.62,1076.47)	-0.004747632	19884.36(14803.49,25754.27)	0.76(0.51,1.05)	-4.50(-4.78,-4.22)
Bahamas	both	1.94(1.38,2.53)	2.71(1.90,3.70)	0.402547901	47.37(32.93,62.02)	0.73(0.52,0.99)	-2.11(-2.30,-1.92)
Gambia	both	2.33(1.50,3.29)	3.96(2.62,5.61)	0.703085931	55.80(35.27,80.84)	0.46(0.31,0.63)	-1.78(-1.90,-1.65)
Timor-Leste	both	3.50(2.03,5.06)	8.20(4.92,11.82)	1.340729565	94.47(52.81,142.38)	1.11(0.69,1.56)	-1.11(-1.35,-0.87)
Togo	both	19.51(12.99,27.10)	34.99(23.86,49.08)	0.793582657	468.05(303.75,660.09)	1.11(0.78,1.53)	-1.64(-1.84,-1.45)
Tonga	both	1.67(1.20,2.16)	1.84(1.31,2.39)	0.102413337	37.83(26.55,50.14)	2.40(1.72,3.11)	-1.26(-1.55,-0.96)
Trinidad and Tobago	both	14.48(11.06,18.00)	9.60(6.40,13.43)	-0.33674	315.55(238.66,394.97)	0.52(0.35,0.7	-4.75(-5.12,-4

				7181		2)	.38)
Tunisia	both	65.95(49.05,85.50)	122.36(84.34,173.69)	0.8553929	1482.69(1070.91,1951.66)	1.03(0.71,1.44)	-1.36(-1.45,-1.28)
Turkey	both	1728.94(1337.30,2181.62)	1969.36(1456.32,2576.59)	0.139054612	47468.66(36452.14,60242.16)	2.24(1.65,2.92)	-2.20(-2.79,-1.61)
Turkmenistan	both	85.13(69.81,99.51)	56.42(41.11,74.89)	-0.3372797	2432.19(1973.49,2857.49)	1.41(1.04,1.85)	-4.14(-4.49,-3.79)
Uganda	both	36.95(19.50,57.26)	61.07(31.53,95.78)	0.6528774	909.05(465.60,1431.55)	0.48(0.26,0.74)	-1.12(-1.47,-0.77)
Ukraine	both	4118.20(3287.05,4902.86)	2034.13(1542.54,2576.30)	-0.506063339	113975.16(90129.17,136917.92)	2.72(2.04,3.47)	-3.46(-3.88,-3.04)
United Arab Emirates	both	5.59(3.78,7.82)	29.23(18.35,42.82)	4.227874434	156.73(104.31,226.30)	1.29(0.91,1.73)	-1.81(-2.16,-1.47)
United Kingdom	both	3214.55(2630.50,3758.30)	1567.78(1237.05,1905.90)	-0.512285615	61991.38(50721.75,72176.61)	1.17(0.93,1.42)	-4.00(-4.17,-3.83)
United States of America	both	4740.91(3858.64,5668.54)	3662.02(2912.80,4493.28)	-0.227569115	99997.03(82877.16,116861.61)	0.65(0.52,0.79)	-2.94(-3.04,-2.83)
Uruguay	both	136.07(108.32,163.53)	100.73(78.15,123.30)	-0.259694538	3058.49(2417.75,3688.23)	1.88(1.47,2.31)	-1.97(-2.05,-1.89)
Uzbekistan	both	288.28(214.05,365.53)	314.82(223.68,415.76)	0.092058818	8076.28(5892.47,10373.73)	1.48(1.09,1.92)	-1.80(-1.98,-1.63)
Vanuatu	both	1.35(0.81,1.99)	2.52(1.44,3.87)	0.86805918	35.18(20.04,54.33)	1.58(0.96,2.36)	-1.33(-1.42,-1.23)
Venezuela (Bolivarian Republic of)	both	306.30(225.41,387.57)	363.75(228.72,524.28)	0.187558742	7108.00(4988.26,9297.56)	1.29(0.83,1.85)	-3.83(-4.08,-3.59)
Viet nam	both	1210.81(852.03,161	1536.42(1102.55,203	0.268919	29782.23(20546.73,4076	1.70(1.23,2.2	-2.60(-2.89,-2

		7.30)	7.31)	389	9.57)	0)	.30)
Virginia	both	99.59(78.96,119.28)	89.62(66.94,116.77)	-0.100145519	2173.47(1760.09,2564.70)	0.62(0.47,0.81)	-3.04(-3.17,-2.90)
Yemen	both	179.22(117.54,254.20)	438.68(304.34,625.33)	1.447635559	4844.35(3118.83,6889.71)	3.63(2.55,5.07)	-0.26(-0.33,-0.18)
Zambia	both	24.16(12.47,37.17)	38.87(20.53,60.25)	0.609139881	569.64(275.91,892.01)	0.68(0.38,1.03)	-1.60(-1.91,-1.29)
Zimbabwe	both	73.60(52.34,95.34)	88.91(61.22,118.97)	0.20810108	1763.86(1208.16,2310.73)	1.44(1.02,1.91)	-1.01(-1.06,-0.96)
Monaco	both	1.85(1.33,2.43)	1.23(0.90,1.59)	-0.333317293	34.95(25.03,46.28)	1.24(0.90,1.61)	-2.44(-2.61,-2.26)
San Marino	both	2.11(1.61,2.70)	2.11(1.30,3.11)	-0.004007323	42.02(31.89,54.06)	3.09(1.91,4.64)	-2.42(-2.54,-2.30)
Saint Kitts and Nevis	both	0.47(0.34,0.62)	0.48(0.34,0.64)	0.018564073	9.93(7.09,13.04)	0.76(0.55,1.01)	-1.84(-2.01,-1.68)
Cook Islands	both	0.20(0.14,0.27)	0.21(0.15,0.29)	0.060570903	5.02(3.39,6.92)	0.87(0.61,1.19)	-2.25(-2.38,-2.13)
Nauru	both	0.11(0.07,0.17)	0.10(0.06,0.15)	-0.14409958	3.58(1.95,5.52)	2.58(1.66,3.60)	-0.78(-1.15,-0.40)
Niue	both	0.04(0.03,0.05)	0.03(0.02,0.04)	-0.322662094	0.90(0.61,1.25)	1.22(0.84,1.63)	-1.45(-1.56,-1.33)
Palau	both	0.19(0.13,0.27)	0.29(0.19,0.42)	0.483608157	5.31(3.41,7.72)	1.37(0.93,1.92)	-1.26(-1.33,-1.19)
Tokelau	both	0.02(0.02,0.03)	0.01(0.01,0.02)	-0.400119516	0.56(0.36,0.77)	1.15(0.77,1.58)	-1.60(-1.64,-1.56)
Tuvalu	both	0.19(0.13,0.26)	0.18(0.12,0.26)	-0.04444	4.99(3.24,6.93)	1.82(1.20,2.5	-1.41(-1.50,-1

				7319		5)	.33)
Afghanistan	female	7.78(3.75,12.27)	24.33(12.74,39.23)	2.128312163	215.59(96.61,351.98)	0.38(0.21,0.59)	1.92(1.83,2.01)
Albania	female	4.80(3.16,6.98)	8.58(5.61,12.50)	0.785982182	96.88(62.28,142.37)	0.37(0.24,0.53)	-1.51(-1.84,-1.18)
Algeria	female	6.12(3.79,9.32)	8.84(5.78,13.10)	0.444068228	136.96(85.45,212.76)	0.07(0.05,0.10)	-2.23(-2.31,-2.15)
American Samoa	female	0.11(0.07,0.17)	0.21(0.12,0.30)	0.825840161	2.92(1.66,4.37)	0.86(0.52,1.25)	-0.59(-0.82,-0.35)
Andorra	female	0.33(0.21,0.50)	0.56(0.36,0.81)	0.707316305	7.63(4.86,11.57)	0.77(0.48,1.10)	-1.85(-2.17,-1.52)
Angola	female	3.30(1.63,5.66)	6.14(3.33,10.18)	0.862756563	90.70(43.86,158.69)	0.11(0.06,0.18)	-2.05(-2.23,-1.86)
Antigua and Barbuda	female	0.11(0.08,0.16)	0.19(0.13,0.26)	0.642458567	2.09(1.43,2.86)	0.37(0.25,0.51)	-0.19(-0.41,0.03)
Argentina	female	257.48(192.66,332.45)	257.60(191.82,325.65)	0.000445759	5629.38(4151.77,7247.86)	0.83(0.61,1.05)	-1.70(-1.83,-1.58)
Armenia	female	4.49(2.62,6.91)	3.16(1.96,4.64)	-0.296255398	119.06(67.24,185.73)	0.13(0.08,0.19)	-2.87(-3.10,-2.63)
Australia	female	88.67(68.47,109.79)	65.70(48.14,83.68)	-0.259010419	1800.70(1397.74,2220.87)	0.28(0.21,0.36)	-3.71(-3.92,-3.49)
Austria	female	104.13(78.41,131.63)	55.21(42.15,69.05)	-0.469857205	2070.19(1572.40,2604.08)	0.54(0.42,0.67)	-3.33(-3.51,-3.15)
Azerbaijan	female	3.43(1.84,5.50)	5.11(2.51,8.60)	0.487994288	96.11(47.82,156.72)	0.10(0.05,0.16)	-0.56(-0.71,-0.40)
Bahrain	female	0.45(0.29,0.67)	0.50(0.31,0.73)	0.097726	10.81(6.90,16.12)	0.21(0.13,0.3	-4.61(-4.86,-4

				145		0)	.36)
Bangladesh	female	33.65(16.96,56.14)	54.35(25.92,100.06)	0.615186742	699.18(342.61,1170.47)	0.10(0.05,0.18)	-2.21(-2.58,-1.85)
Barbados	female	0.41(0.28,0.56)	0.41(0.27,0.58)	0.001933872	8.95(6.07,12.61)	0.15(0.10,0.21)	-1.52(-1.70,-1.33)
Belarus	female	94.15(59.62,135.35)	41.12(25.76,61.45)	-0.563281533	2684.87(1655.96,3891.93)	0.46(0.28,0.69)	-4.19(-4.47,-3.91)
Belgium	female	127.31(94.98,161.47)	65.75(50.47,82.32)	-0.483554388	2214.24(1691.58,2739.10)	0.48(0.37,0.59)	-3.51(-3.80,-3.22)
Belize	female	0.19(0.12,0.27)	0.31(0.20,0.45)	0.658494313	3.75(2.43,5.33)	0.25(0.16,0.36)	-1.58(-1.87,-1.30)
Benin	female	1.97(0.98,3.37)	2.71(1.46,4.35)	0.375256392	46.31(22.58,80.07)	0.12(0.06,0.19)	-1.16(-1.44,-0.88)
Bermuda	female	0.16(0.11,0.23)	0.14(0.09,0.20)	-0.146227597	3.20(2.00,4.57)	0.17(0.11,0.25)	-3.92(-4.22,-3.62)
Bhutan	female	0.29(0.15,0.48)	0.46(0.26,0.77)	0.60764421	7.65(3.80,13.19)	0.18(0.10,0.29)	-1.19(-1.24,-1.14)
Bolivia (Plurinational State of)	female	24.75(10.78,42.66)	31.74(13.75,55.16)	0.282181032	592.98(251.39,1043.43)	0.72(0.32,1.26)	-2.80(-3.11,-2.48)
Bosnia and Herzegovina	female	24.86(17.99,32.54)	35.27(24.70,47.63)	0.418762576	628.01(450.89,820.88)	1.05(0.73,1.41)	-0.31(-0.54,-0.07)
Botswana	female	1.13(0.60,1.94)	1.85(1.03,2.92)	0.629457974	24.33(11.78,42.42)	0.29(0.17,0.45)	-1.42(-1.67,-1.18)
Brazil	female	932.14(701.13,1166.78)	867.58(648.57,1098.03)	-0.069261683	21498.46(16150.42,26919.71)	0.67(0.50,0.85)	-4.09(-4.28,-3.90)
Brunei Darussalam	female	0.87(0.60,1.22)	0.78(0.54,1.08)	-0.09932	22.04(15.10,31.61)	0.62(0.44,0.8	-4.49(-4.74,-4

				3364		5)	.23)
Bulgaria	female	126.90(91.36,167.64)	66.67(46.75,90.80)	-0.474642036	3403.85(2477.74,4476.96)	0.90(0.61,1.22)	-1.69(-2.10,-1.28)
Burkina Faso	female	1.50(0.63,2.82)	2.30(0.98,4.05)	0.535875074	35.33(14.13,65.39)	0.05(0.02,0.09)	-0.83(-1.08,-0.58)
Burundi	female	2.33(0.94,4.44)	2.08(0.80,3.81)	-0.107248567	59.43(22.77,115.75)	0.10(0.04,0.18)	-3.05(-3.34,-2.76)
Cambodia	female	11.70(6.47,18.20)	17.45(10.22,26.40)	0.491416394	273.37(144.56,433.90)	0.29(0.18,0.44)	-1.81(-1.96,-1.67)
Cameroon	female	2.13(1.05,3.62)	3.66(1.79,6.34)	0.720476897	48.04(22.97,84.31)	0.08(0.04,0.13)	-0.94(-1.11,-0.78)
Canada	female	212.76(168.94,256.22)	178.63(135.94,224.10)	-0.160422642	4229.71(3381.08,5059.16)	0.45(0.35,0.56)	-3.30(-3.37,-3.22)
Cabo Verde	female	0.83(0.42,1.35)	0.54(0.30,0.86)	-0.344451818	16.41(7.91,26.70)	0.23(0.13,0.37)	-3.27(-3.90,-2.64)
Central African Republic	female	0.84(0.39,1.49)	0.92(0.42,1.72)	0.098035244	22.66(10.22,41.20)	0.09(0.04,0.16)	-1.92(-2.13,-1.72)
Chad	female	2.70(1.23,4.66)	4.01(1.92,6.90)	0.482598352	56.66(25.16,100.53)	0.19(0.09,0.32)	0.55(0.26,0.85)
Chile	female	148.50(106.60,200.63)	159.79(108.82,213.24)	0.076083759	3551.49(2449.39,4880.96)	1.20(0.81,1.60)	-2.84(-2.91,-2.77)
China	female	2668.80(1636.74,3938.68)	4245.28(2883.37,5900.84)	0.590706369	53446.33(31832.16,78788.99)	0.43(0.30,0.59)	-1.86(-2.65,-1.06)
Colombia	female	183.89(118.73,256.60)	133.66(82.57,198.51)	-0.273154864	4185.73(2571.02,5805.30)	0.46(0.29,0.69)	-5.61(-5.92,-5.30)
Comoros	female	0.11(0.05,0.19)	0.20(0.10,0.32)	0.753882	2.57(1.04,4.56)	0.08(0.04,0.1	-1.07(-1.21,-0

				374		3)	.93)
Congo	female	0.62(0.29,1.11)	0.78(0.37,1.33)	0.269065334	15.94(7.15,28.66)	0.06(0.03,0.11)	-1.90(-2.04,-1.76)
Costa Rica	female	14.99(9.93,20.92)	17.81(11.24,25.57)	0.187909245	332.84(211.30,470.06)	0.64(0.40,0.92)	-3.64(-3.87,-3.40)
C 么 te d'Ivoire	female	3.85(2.15,6.11)	10.54(6.02,16.91)	1.739636956	88.47(48.22,141.88)	0.27(0.16,0.43)	0.79(0.46,1.12)
Croatia	female	100.71(75.86,128.66)	45.87(33.40,62.17)	-0.544491061	2251.12(1691.54,2829.83)	0.89(0.65,1.21)	-3.85(-4.13,-3.57)
Cuba	female	35.29(25.35,45.94)	48.90(33.69,65.68)	0.385684554	782.87(559.60,1021.54)	0.48(0.33,0.64)	-0.78(-0.92,-0.65)
Cyprus	female	3.21(2.22,4.43)	5.87(4.24,7.80)	0.827643776	71.72(49.41,98.57)	0.56(0.41,0.74)	-0.82(-1.06,-0.57)
Czechia	female	149.96(108.65,196.58)	69.96(50.54,93.35)	-0.533442839	3072.73(2251.80,3934.83)	0.57(0.41,0.76)	-4.13(-4.23,-4.03)
Democratic Republic of the Congo	female	8.12(3.79,14.44)	9.82(4.54,17.85)	0.209501122	218.41(98.68,388.58)	0.05(0.03,0.10)	-2.16(-2.19,-2.13)
Denmark	female	90.45(72.75,108.16)	39.95(30.86,49.35)	-0.558281069	1665.52(1359.25,1993.40)	0.60(0.47,0.74)	-3.68(-3.82,-3.54)
Djibouti	female	0.08(0.04,0.14)	0.31(0.15,0.55)	2.889409359	2.02(0.92,3.77)	0.15(0.07,0.25)	-0.29(-0.42,-0.16)
Dominica	female	0.41(0.26,0.58)	0.25(0.16,0.36)	-0.388308097	7.77(4.97,11.10)	0.51(0.34,0.74)	-1.85(-2.05,-1.64)
Dominican Republic	female	11.34(8.17,15.59)	32.75(22.48,46.19)	1.887776895	234.08(163.15,322.31)	0.71(0.49,1.01)	0.73(0.43,1.03)
Ecuador	female	29.83(18.55,43.04)	43.69(26.04,66.02)	0.464416	634.82(367.65,935.82)	0.58(0.35,0.8	-2.39(-2.65,-2

				16		7)	.12)
Egypt	female	4.26(2.75,6.22)	7.88(4.66,12.44)	0.851829375	86.44(55.59,126.43)	0.04(0.03,0.07)	0.71(0.56,0.86)
El Salvador	female	8.80(5.69,12.56)	13.29(8.23,20.08)	0.510748227	206.08(133.41,297.11)	0.39(0.24,0.59)	-1.78(-2.01,-1.54)
Equatorial Guinea	female	0.09(0.04,0.16)	0.11(0.05,0.21)	0.296368089	2.26(0.91,4.31)	0.05(0.02,0.08)	-2.15(-2.24,-2.06)
Eritrea	female	0.13(0.05,0.24)	0.27(0.11,0.49)	1.112915265	3.56(1.30,6.73)	0.02(0.01,0.04)	-0.65(-0.87,-0.44)
Estonia	female	23.96(16.41,32.50)	14.07(9.49,19.46)	-0.41281025	585.44(394.97,791.52)	0.88(0.58,1.23)	-2.93(-3.35,-2.51)
Ethiopia	female	4.69(1.74,9.13)	3.98(1.53,7.79)	-0.150504166	133.71(48.07,264.04)	0.02(0.01,0.04)	-3.62(-3.87,-3.38)
Micronesia (Federated States of)	female	0.34(0.18,0.57)	0.42(0.22,0.68)	0.220235102	8.76(4.43,15.01)	1.23(0.67,1.98)	-0.65(-0.83,-0.48)
Fiji	female	0.94(0.52,1.50)	0.95(0.47,1.60)	0.011167415	25.39(12.86,42.10)	0.26(0.13,0.43)	-2.41(-2.61,-2.21)
Finland	female	57.37(41.96,73.97)	26.50(19.58,33.84)	-0.538171422	1229.87(921.49,1550.36)	0.39(0.29,0.48)	-4.26(-4.57,-3.96)
France	female	355.41(260.51,458.58)	295.79(219.85,380.03)	-0.167751598	6402.69(4807.37,8097.25)	0.35(0.26,0.44)	-2.61(-2.80,-2.42)
Gabon	female	0.31(0.13,0.56)	0.29(0.14,0.49)	-0.061453951	7.48(3.16,13.47)	0.06(0.03,0.10)	-2.13(-2.32,-1.93)
Georgia	female	14.40(8.92,20.99)	9.19(5.81,12.95)	-0.362250684	372.77(218.67,563.53)	0.26(0.16,0.37)	-0.63(-1.18,-0.07)
Germany	female	1281.20(968.81,160	756.42(577.13,951.06	-0.40960	24785.47(19132.44,3065	0.68(0.53,0.8	-3.09(-3.26,-2

		7.48)	)	0492	6.39)	4)	.92)
Ghana	female	4.45(2.47,6.84)	7.16(4.17,11.18)	0.608040485	104.06(55.83,165.24)	0.10(0.06,0.15)	-1.52(-1.86,-1.19)
Greece	female	105.29(77.22,135.49)	121.75(94.89,151.34)	0.156326556	2201.20(1631.77,2791.92)	0.94(0.73,1.15)	-1.46(-1.74,-1.17)
Greenland	female	0.49(0.36,0.63)	0.43(0.31,0.58)	-0.110483421	12.89(9.11,17.12)	1.40(1.01,1.88)	-3.26(-3.62,-2.90)
Grenada	female	0.21(0.14,0.29)	0.18(0.12,0.25)	-0.12037076	4.00(2.55,5.72)	0.32(0.21,0.44)	-1.08(-1.39,-0.76)
Guam	female	0.14(0.08,0.22)	0.27(0.17,0.40)	0.873659171	3.71(2.06,5.82)	0.28(0.17,0.41)	-1.79(-2.29,-1.30)
Guatemala	female	16.81(9.11,26.39)	37.46(20.28,59.17)	1.227809763	411.14(215.36,653.83)	0.67(0.37,1.05)	-2.00(-2.46,-1.54)
Guinea	female	2.34(1.21,3.92)	3.52(1.85,5.67)	0.503151499	52.50(26.79,90.84)	0.14(0.08,0.23)	-0.22(-0.33,-0.10)
Guinea-Bissau	female	0.26(0.12,0.46)	0.30(0.16,0.51)	0.179240152	6.54(2.90,11.94)	0.09(0.05,0.14)	-1.11(-1.55,-0.67)
Guyana	female	0.85(0.54,1.26)	0.73(0.43,1.08)	-0.148490261	19.96(12.04,30.74)	0.24(0.14,0.35)	-2.50(-2.67,-2.34)
Haiti	female	8.57(4.33,14.22)	10.32(4.70,18.45)	0.203882907	225.46(103.39,381.40)	0.31(0.15,0.54)	-1.88(-2.03,-1.73)
Honduras	female	5.29(3.06,7.98)	14.73(8.51,23.69)	1.783814145	132.01(73.43,205.93)	0.48(0.28,0.76)	-0.09(-0.32,0.14)
Hungary	female	161.99(123.00,205.82)	99.25(72.24,131.85)	-0.387278751	3693.28(2802.32,4692.29)	0.87(0.63,1.16)	-2.64(-2.74,-2.54)
Iceland	female	2.64(2.01,3.36)	1.15(0.85,1.49)	-0.56660	52.36(40.11,65.76)	0.36(0.28,0.4	-5.40(-5.61,-5

				1058		7)	.20)
India	female	530.62(308.86,803.41)	1014.43(638.66,1471.59)	0.911802222	13343.21(7440.26,20842.76)	0.19(0.12,0.27)	-1.70(-1.92,-1.48)
Indonesia	female	60.11(31.61,96.83)	123.09(68.22,190.81)	1.047808255	1429.28(716.28,2422.29)	0.14(0.08,0.21)	0.12(0.04,0.20)
Iran (Islamic Republic of)	female	58.89(35.33,90.58)	97.96(65.12,133.19)	0.663580737	1513.28(887.49,2304.85)	0.28(0.19,0.38)	-1.77(-2.16,-1.38)
Iraq	female	7.82(4.84,11.95)	18.17(11.80,25.52)	1.32458088	187.23(111.86,291.45)	0.17(0.11,0.23)	-0.41(-0.64,-0.19)
Ireland	female	50.53(39.39,61.32)	29.83(22.52,37.25)	-0.409762679	916.49(717.39,1110.80)	0.71(0.54,0.88)	-4.03(-4.21,-3.84)
Israel	female	29.36(22.07,37.43)	29.21(22.00,37.86)	-0.005197558	635.65(482.61,804.31)	0.45(0.34,0.57)	-3.80(-4.02,-3.58)
Italy	female	881.04(666.61,1115.12)	631.99(480.57,793.87)	-0.282673371	17337.52(13415.15,21730.16)	0.74(0.57,0.92)	-3.32(-3.53,-3.10)
Jamaica	female	5.52(3.92,7.66)	5.30(3.60,7.54)	-0.039675243	108.49(76.33,149.22)	0.33(0.22,0.47)	-1.81(-2.11,-1.52)
Japan	female	2031.27(1547.41,2551.91)	1215.43(863.63,1601.58)	-0.401638427	42273.23(32128.12,53291.08)	0.56(0.42,0.71)	-4.52(-4.69,-4.34)
Jordan	female	2.41(1.57,3.56)	6.33(4.18,9.21)	1.622020303	61.91(38.31,94.48)	0.26(0.18,0.36)	-1.80(-1.99,-1.62)
Kazakhstan	female	45.98(26.73,71.64)	24.77(14.15,36.99)	-0.461288226	1241.34(686.79,1912.73)	0.24(0.14,0.36)	-3.26(-3.46,-3.07)
Kenya	female	5.24(2.50,9.27)	13.77(6.97,24.53)	1.627040451	113.62(51.21,206.37)	0.14(0.08,0.25)	0.71(0.29,1.13)
Kiribati	female	0.26(0.16,0.39)	0.45(0.28,0.65)	0.739117	6.59(3.86,10.11)	1.41(0.90,1.9	0.23(0.13,0.3

				261		8)	4)
Kuwait	female	0.32(0.20,0.47)	0.50(0.32,0.71)	0.571986294	7.09(4.38,10.52)	0.06(0.04,0.09)	-2.99(-3.40,-2.58)
Kyrgyzstan	female	8.20(5.11,11.91)	6.11(4.02,8.46)	-0.254862644	211.68(131.67,311.75)	0.24(0.16,0.33)	-2.68(-3.38,-1.98)
Lao People's Democratic Republic	female	5.93(3.15,9.67)	4.55(2.52,7.27)	-0.232529196	147.50(75.39,247.45)	0.24(0.13,0.38)	-3.38(-3.70,-3.06)
Latvia	female	29.32(18.57,41.37)	13.46(8.27,19.83)	-0.540966782	765.37(476.65,1078.21)	0.59(0.36,0.90)	-3.23(-3.70,-2.75)
Lebanon	female	9.98(6.60,14.06)	24.16(17.04,33.18)	1.419852185	236.32(149.48,342.16)	0.84(0.59,1.16)	-0.26(-0.40,-0.13)
Lesotho	female	1.24(0.75,1.91)	1.60(0.90,2.58)	0.284449755	20.00(12.07,30.93)	0.29(0.16,0.46)	1.12(0.82,1.42)
Liberia	female	0.98(0.46,1.65)	1.07(0.51,1.93)	0.094602582	22.25(10.19,38.36)	0.13(0.06,0.22)	-0.98(-1.32,-0.64)
Libya	female	0.37(0.21,0.64)	0.56(0.32,0.88)	0.532545191	8.61(4.54,15.14)	0.02(0.01,0.04)	-2.11(-2.42,-1.81)
Lithuania	female	23.95(15.06,34.11)	14.11(9.24,19.87)	-0.410972103	665.10(409.77,943.66)	0.45(0.29,0.64)	-2.62(-3.06,-2.17)
Luxembourg	female	4.33(3.13,5.63)	2.73(2.00,3.60)	-0.369729563	86.97(63.55,112.32)	0.47(0.34,0.61)	-3.56(-3.63,-3.50)
North Macedonia	female	21.90(15.46,29.04)	24.11(16.53,32.99)	0.100642953	562.87(387.92,752.06)	1.42(0.97,1.92)	-1.81(-1.93,-1.69)
Madagascar	female	2.54(1.15,4.49)	2.76(1.18,4.94)	0.086340602	68.61(29.77,120.55)	0.06(0.03,0.10)	-2.67(-2.94,-2.40)
Malawi	female	1.35(0.59,2.39)	1.74(0.77,3.01)	0.293188	29.10(12.44,52.46)	0.05(0.02,0.0	-1.41(-1.55,-1

				174		8)	.28)
Malaysia	female	7.34(4.30,11.33)	12.91(7.77,20.02)	0.757418809	155.17(86.80,248.26)	0.12(0.07,0.19)	-1.53(-1.85,-1.21)
Maldives	female	0.11(0.05,0.19)	0.13(0.08,0.20)	0.174043729	2.86(1.17,5.06)	0.12(0.07,0.17)	-5.05(-5.36,-4.74)
Mali	female	2.75(1.14,5.08)	6.12(2.55,11.03)	1.228580545	69.53(27.53,129.49)	0.16(0.07,0.28)	0.44(0.39,0.48)
Malta	female	2.25(1.66,2.90)	1.59(1.19,2.09)	-0.29173396	50.24(37.42,64.26)	0.32(0.24,0.41)	-4.32(-4.58,-4.06)
Marshall Islands	female	0.05(0.03,0.07)	0.07(0.04,0.12)	0.617114507	1.15(0.64,1.85)	0.50(0.28,0.79)	-0.60(-0.68,-0.52)
Mauritania	female	1.03(0.48,1.75)	1.43(0.64,2.58)	0.389102519	28.38(12.71,49.18)	0.14(0.07,0.25)	-0.99(-1.13,-0.85)
Mauritius	female	1.05(0.62,1.60)	0.99(0.57,1.47)	-0.066167911	23.18(12.97,35.76)	0.10(0.06,0.15)	-3.77(-4.04,-3.50)
Mexico	female	189.55(121.52,272.74)	166.48(96.38,247.91)	-0.121710315	3953.80(2407.44,5850.78)	0.28(0.16,0.41)	-4.21(-4.30,-4.13)
Republic of Moldova	female	9.45(5.75,13.92)	5.88(3.76,8.47)	-0.377887649	261.50(156.23,383.79)	0.17(0.11,0.25)	-1.73(-2.18,-1.29)
Mongolia	female	7.98(4.17,12.99)	10.54(5.49,17.20)	0.321356821	177.64(87.02,293.23)	0.96(0.52,1.53)	-2.03(-2.31,-1.75)
Montenegro	female	3.30(2.34,4.43)	5.11(3.74,6.70)	0.549659796	79.72(57.64,107.21)	0.91(0.66,1.18)	-0.11(-0.41,0.20)
Morocco	female	3.19(1.98,4.73)	4.89(2.99,7.36)	0.533773426	80.15(47.93,123.99)	0.03(0.02,0.05)	-1.27(-1.38,-1.15)
Mozambique	female	3.44(1.47,5.95)	5.68(2.43,10.31)	0.652846	76.93(32.54,134.84)	0.11(0.05,0.1	-0.22(-0.45,0.

				226		9)	01)
Myanmar	female	150.83(89.06,230.18)	100.90(59.53,153.17)	-0.330987235	3393.64(1920.08,5277.74)	0.45(0.26,0.67)	-3.92(-4.19,-3.64)
Namibia	female	1.15(0.67,1.69)	1.65(0.97,2.57)	0.439641653	21.97(12.36,33.27)	0.24(0.14,0.36)	-1.20(-1.38,-1.03)
Nepal	female	38.29(19.54,61.06)	76.80(39.55,124.17)	1.005534963	936.94(439.20,1582.15)	0.77(0.41,1.19)	-0.41(-0.64,-0.19)
Netherlands	female	260.71(204.90,318.90)	190.91(144.86,240.31)	-0.267706246	4795.33(3784.95,5764.98)	0.94(0.72,1.16)	-2.97(-3.19,-2.75)
New Zealand	female	28.28(21.99,34.74)	18.30(13.74,23.30)	-0.352933036	571.87(443.52,701.02)	0.43(0.32,0.54)	-3.74(-3.80,-3.68)
Nicaragua	female	2.61(1.51,3.93)	4.76(2.76,7.35)	0.820755374	75.41(41.54,117.92)	0.20(0.12,0.31)	-2.19(-2.66,-1.72)
Niger	female	0.61(0.26,1.07)	2.06(0.87,3.72)	2.384491154	15.87(6.65,28.72)	0.06(0.03,0.10)	0.73(0.60,0.86)
Nigeria	female	10.34(5.33,17.55)	10.30(5.26,17.49)	-0.003809367	205.10(102.44,349.23)	0.03(0.01,0.05)	-2.28(-2.53,-2.03)
Democratic People's Republic of Korea	female	39.86(21.21,66.61)	74.21(40.88,118.84)	0.861812302	961.91(513.39,1618.20)	0.39(0.21,0.62)	-0.45(-0.78,-0.13)
Northern Mariana Islands	female	0.07(0.04,0.12)	0.12(0.07,0.20)	0.676768692	2.11(1.04,3.46)	0.54(0.30,0.84)	-2.92(-3.26,-2.57)
Norway	female	44.13(33.10,57.25)	17.40(12.83,22.48)	-0.605732836	887.12(671.15,1134.01)	0.34(0.25,0.43)	-4.05(-4.43,-3.67)
Oman	female	0.53(0.31,0.87)	0.45(0.27,0.66)	-0.164533465	12.22(6.86,20.19)	0.08(0.05,0.13)	-3.23(-3.38,-3.08)
Pakistan	female	44.55(25.47,67.87)	63.27(37.79,95.85)	0.420325	1065.76(591.28,1615.61)	0.13(0.08,0.1	-0.87(-1.26,-0

				741		9)	.47)
Palestine	female	0.90(0.51,1.49)	1.34(0.82,1.89)	0.497221963	20.23(10.96,34.89)	0.13(0.08,0.18)	-1.63(-1.71,-1.55)
Panama	female	4.40(2.95,6.03)	5.74(3.64,8.25)	0.303456512	90.06(60.34,127.22)	0.26(0.16,0.37)	-2.88(-3.06,-2.70)
Papua New Guinea	female	6.37(2.99,10.69)	13.36(6.59,22.97)	1.098464939	165.20(76.26,286.10)	0.70(0.36,1.18)	-0.03(-0.22,0.17)
Paraguay	female	11.01(7.36,15.41)	14.49(9.16,21.25)	0.31617579	211.58(138.41,302.93)	0.51(0.32,0.75)	-2.75(-3.36,-2.13)
Peru	female	21.37(10.48,34.93)	37.92(17.90,64.64)	0.774624116	505.64(238.39,841.60)	0.23(0.11,0.39)	-1.76(-2.08,-1.44)
Philippines	female	52.68(31.51,78.75)	82.55(51.75,119.06)	0.567082563	1089.09(632.32,1672.24)	0.24(0.15,0.34)	-2.08(-2.24,-1.91)
Poland	female	409.81(310.99,525.17)	355.27(252.84,472.39)	-0.13308604	9465.78(7189.08,11871.19)	0.86(0.62,1.14)	-2.34(-2.51,-2.18)
Portugal	female	89.05(63.54,119.63)	52.33(37.76,68.17)	-0.412435072	2080.15(1481.89,2761.11)	0.45(0.32,0.59)	-3.61(-3.87,-3.35)
Puerto Rico	female	9.18(6.25,12.73)	9.31(6.11,13.51)	0.01339681	177.52(117.42,246.08)	0.21(0.14,0.30)	-3.24(-3.42,-3.06)
Qatar	female	0.05(0.03,0.07)	0.12(0.07,0.20)	1.69569538	1.31(0.70,2.18)	0.09(0.05,0.14)	-1.21(-1.56,-0.86)
Romania	female	138.72(92.15,194.06)	100.70(69.78,138.03)	-0.274091185	3568.35(2371.92,4900.96)	0.49(0.34,0.68)	-2.15(-2.27,-2.04)
Russian Federation	female	811.60(506.51,1182.07)	683.16(451.68,955.10)	-0.15825178	21679.06(12918.76,32736.46)	0.49(0.32,0.69)	-1.47(-1.87,-1.08)
Rwanda	female	6.32(3.09,10.47)	11.84(6.74,17.96)	0.872383	140.33(66.10,240.05)	0.43(0.25,0.6	-0.94(-1.18,-0

				041		5)	.69)
Saint Lucia	female	0.32(0.22,0.44)	0.34(0.23,0.47)	0.072598612	6.66(4.46,9.28)	0.30(0.20,0.41)	-3.25(-3.62,-2.88)
Saint Vincent and the Grenadines	female	0.16(0.11,0.23)	0.16(0.11,0.24)	-0.009555244	3.20(2.04,4.52)	0.25(0.16,0.37)	-1.59(-1.97,-1.22)
Samoa	female	0.41(0.25,0.63)	0.58(0.36,0.84)	0.427615922	10.08(6.11,15.99)	0.82(0.51,1.19)	-0.43(-0.52,-0.34)
Sao Tome and Principe	female	0.05(0.02,0.09)	0.09(0.04,0.15)	0.725859618	1.08(0.48,1.90)	0.19(0.09,0.33)	0.94(0.84,1.04)
Saudi Arabia	female	1.67(0.99,2.54)	2.64(1.66,4.05)	0.580166341	39.05(23.32,60.85)	0.05(0.03,0.08)	-1.47(-1.61,-1.34)
Senegal	female	1.33(0.59,2.40)	2.29(1.08,4.15)	0.726006048	32.42(13.94,59.17)	0.06(0.03,0.11)	-0.59(-0.90,-0.28)
Serbia	female	76.80(52.08,104.87)	75.50(52.45,103.66)	-0.016953636	1991.19(1311.46,2778.59)	0.87(0.60,1.20)	-1.81(-2.03,-1.58)
Seychelles	female	0.06(0.04,0.10)	0.07(0.04,0.10)	0.062425361	1.30(0.77,2.02)	0.12(0.07,0.18)	-1.73(-1.89,-1.57)
Sierra Leone	female	3.05(1.24,5.30)	3.67(1.46,6.49)	0.204230296	77.75(30.41,137.38)	0.22(0.09,0.38)	-0.80(-1.12,-0.48)
Singapore	female	8.97(6.34,12.35)	7.01(5.01,9.52)	-0.218651876	219.98(152.84,300.98)	0.17(0.12,0.23)	-5.32(-5.50,-5.15)
Slovakia	female	36.73(25.62,49.87)	24.26(15.91,34.24)	-0.339528943	955.49(656.05,1286.30)	0.46(0.30,0.66)	-2.88(-3.15,-2.62)
Slovenia	female	28.23(18.88,39.42)	17.64(12.22,24.09)	-0.37489138	644.02(431.39,903.92)	0.73(0.51,1.00)	-3.66(-3.91,-3.41)
Solomon Islands	female	0.79(0.40,1.36)	1.66(0.88,2.69)	1.103321	23.09(10.77,40.71)	1.20(0.68,1.9	-0.60(-0.72,-0

				659		3)	.47)
Somalia	female	1.42(0.57,2.70)	3.42(1.29,6.90)	1.400470223	36.46(14.20,70.42)	0.11(0.04,0.21)	-0.49(-0.55,-0.43)
South Africa	female	69.16(46.67,97.68)	58.04(40.28,77.85)	-0.160834114	1618.87(1068.94,2255.52)	0.24(0.16,0.32)	-3.48(-3.94,-3.02)
Republic of Korea	female	300.05(206.44,408.27)	211.78(147.59,286.53)	-0.294193016	7548.10(5137.67,10301.03)	0.42(0.29,0.56)	-6.01(-6.31,-5.71)
South Sudan	female	1.14(0.45,2.23)	1.20(0.45,2.36)	0.05520523	24.38(9.16,48.15)	0.08(0.03,0.16)	-1.28(-1.32,-1.25)
Spain	female	278.02(201.17,361.50)	255.54(194.98,321.07)	-0.080857809	6510.61(4732.58,8286.94)	0.54(0.41,0.68)	-1.76(-2.32,-1.20)
Sri Lanka	female	8.61(5.02,13.16)	8.80(5.14,13.84)	0.022122168	183.48(103.79,287.99)	0.07(0.04,0.10)	-3.77(-3.92,-3.62)
Sudan	female	9.23(4.87,14.88)	15.20(8.61,23.74)	0.646903768	238.98(126.81,391.63)	0.20(0.11,0.31)	0.00(-0.15,0.16)
Suriname	female	0.59(0.40,0.84)	0.96(0.61,1.39)	0.621177362	13.53(8.75,19.72)	0.30(0.19,0.43)	-1.58(-1.85,-1.31)
Eswatini	female	0.43(0.24,0.67)	0.52(0.28,0.86)	0.218362205	7.25(4.01,11.37)	0.20(0.11,0.33)	-1.31(-1.71,-0.91)
Sweden	female	110.97(84.63,138.25)	59.35(45.08,74.15)	-0.465158019	2029.54(1577.72,2489.58)	0.48(0.37,0.60)	-3.21(-3.38,-3.04)
Switzerland	female	59.67(44.48,75.41)	40.99(30.69,52.45)	-0.313077896	1165.40(888.41,1444.22)	0.40(0.30,0.50)	-2.94(-3.22,-2.66)
Syrian Arab Republic	female	4.52(2.78,6.71)	6.64(4.02,10.35)	0.469427434	114.27(65.94,174.54)	0.13(0.08,0.20)	-1.56(-1.81,-1.31)
Taiwan (Province of	female	20.43(12.10,29.38)	20.94(12.48,31.35)	0.025217	576.66(335.81,833.52)	0.10(0.06,0.1	-3.52(-3.70,-3

China)				499		5)	.34)
Tajikistan	female	6.05(3.59,9.28)	6.37(3.82,9.56)	0.051983996	162.76(94.86,249.02)	0.25(0.15,0.37)	-1.22(-1.50,-0.95)
United Republic of Tanzania	female	20.03(8.95,35.17)	36.98(17.66,63.31)	0.846205213	475.68(208.53,853.00)	0.32(0.16,0.56)	-0.59(-0.66,-0.51)
Thailand	female	67.05(41.14,100.09)	55.66(33.78,85.20)	-0.169965806	1482.67(863.59,2248.76)	0.10(0.06,0.15)	-5.19(-5.48,-4.89)
Bahamas	female	0.34(0.22,0.47)	0.50(0.33,0.74)	0.498268698	7.62(4.82,10.65)	0.25(0.17,0.36)	-1.67(-1.83,-1.51)
Gambia	female	0.07(0.03,0.12)	0.14(0.07,0.23)	0.934493434	1.36(0.60,2.45)	0.03(0.02,0.05)	-1.69(-1.83,-1.55)
Timor-Leste	female	0.28(0.12,0.49)	0.52(0.23,0.93)	0.854508253	7.08(2.85,12.75)	0.14(0.07,0.26)	-2.10(-2.27,-1.92)
Togo	female	1.96(1.10,3.03)	4.22(2.45,6.49)	1.15913923	43.79(24.01,68.99)	0.24(0.14,0.36)	-1.21(-1.33,-1.09)
Tonga	female	0.18(0.11,0.27)	0.20(0.12,0.31)	0.106609597	4.07(2.28,6.26)	0.47(0.28,0.72)	-1.40(-1.50,-1.29)
Trinidad and Tobago	female	2.28(1.58,3.09)	1.45(0.90,2.19)	-0.366502861	47.23(32.67,64.00)	0.15(0.09,0.23)	-5.08(-5.48,-4.68)
Tunisia	female	2.61(1.62,3.92)	4.57(2.78,7.04)	0.749268965	59.59(36.48,89.99)	0.07(0.04,0.11)	-1.69(-1.78,-1.59)
Turkey	female	179.38(110.90,263.46)	205.22(138.24,284.28)	0.144050048	5236.28(3163.80,7857.50)	0.44(0.30,0.61)	-2.41(-3.04,-1.77)
Turkmenistan	female	4.98(3.12,7.13)	3.00(1.78,4.63)	-0.397801893	137.75(83.23,199.57)	0.13(0.08,0.20)	-4.55(-5.04,-4.05)
Uganda	female	3.89(1.67,7.12)	8.81(4.04,14.85)	1.266992	79.75(32.87,146.83)	0.14(0.06,0.2	-0.12(-0.38,0.

				827		3)	14)
Ukraine	female	315.58(190.71,473.08)	149.20(88.60,222.83)	-0.527204249	9066.18(5253.91,13785.50)	0.35(0.20,0.54)	-3.36(-3.82,-2.89)
United Arab Emirates	female	0.43(0.25,0.71)	1.34(0.75,2.16)	2.146490681	11.66(6.58,19.87)	0.16(0.10,0.24)	-2.29(-3.31,-1.26)
United Kingdom	female	1110.15(889.81,1322.48)	558.29(433.36,689.26)	-0.49710308	19607.48(15886.57,23370.12)	0.74(0.58,0.90)	-3.60(-3.74,-3.46)
United States of America	female	1618.84(1290.15,1966.10)	1238.47(969.42,1532.00)	-0.234965432	31639.72(25571.57,37430.61)	0.39(0.31,0.48)	-2.73(-2.81,-2.65)
Uruguay	female	26.43(18.61,34.87)	22.63(16.11,29.65)	-0.143579994	583.42(405.26,767.08)	0.73(0.52,0.95)	-1.31(-1.46,-1.17)
Uzbekistan	female	9.95(5.22,15.27)	12.95(6.46,21.41)	0.302061593	271.39(139.11,432.80)	0.10(0.05,0.16)	-1.34(-1.55,-1.13)
Vanuatu	female	0.08(0.04,0.14)	0.16(0.08,0.27)	0.903780268	1.87(0.89,3.22)	0.23(0.12,0.38)	-1.37(-1.50,-1.23)
Venezuela (Bolivarian Republic of)	female	72.23(46.34,101.51)	80.13(46.96,124.20)	0.109384018	1632.03(1032.02,2346.91)	0.54(0.31,0.82)	-3.94(-4.30,-3.58)
Viet nam	female	51.60(26.69,85.78)	53.52(29.02,83.56)	0.037273782	1049.42(517.42,1788.85)	0.11(0.06,0.17)	-2.87(-3.48,-2.27)
Virginia	female	33.16(25.37,41.77)	30.14(21.48,39.97)	-0.091109085	660.82(516.19,807.95)	0.38(0.27,0.50)	-2.72(-2.82,-2.63)
Yemen	female	19.81(11.16,31.39)	50.01(31.89,73.85)	1.525085504	524.72(281.60,847.49)	0.80(0.52,1.18)	-0.20(-0.29,-0.12)
Zambia	female	2.94(1.38,4.90)	5.73(2.80,9.36)	0.950239409	58.85(26.16,100.02)	0.22(0.11,0.35)	-0.94(-1.12,-0.75)
Zimbabwe	female	10.72(6.43,16.01)	15.88(9.38,24.59)	0.481832	201.77(122.04,314.73)	0.49(0.30,0.7	0.54(-0.04,1.1

				318		5)	1)
Monaco	female	0.50(0.32,0.72)	0.30(0.20,0.44)	-0.396320021	9.05(5.78,12.88)	0.56(0.36,0.81)	-2.51(-2.77,-2.25)
San Marino	female	0.52(0.36,0.71)	0.48(0.28,0.77)	-0.073510252	9.91(6.90,13.38)	1.32(0.77,2.14)	-2.28(-2.43,-2.14)
Saint Kitts and Nevis	female	0.09(0.06,0.12)	0.06(0.04,0.09)	-0.30410354	1.62(1.10,2.30)	0.20(0.13,0.28)	-1.66(-1.90,-1.41)
Cook Islands	female	0.03(0.02,0.05)	0.03(0.02,0.05)	0.076520051	0.71(0.38,1.12)	0.25(0.15,0.39)	-2.44(-2.67,-2.21)
Nauru	female	0.03(0.01,0.05)	0.03(0.01,0.05)	0.036560606	0.83(0.40,1.42)	1.30(0.71,2.05)	-0.58(-0.87,-0.29)
Niue	female	0.01(0.00,0.01)	0.01(0.00,0.01)	-0.359030551	0.17(0.09,0.26)	0.42(0.25,0.63)	-1.43(-1.52,-1.35)
Palau	female	0.01(0.01,0.02)	0.02(0.01,0.03)	0.294893333	0.32(0.16,0.53)	0.17(0.10,0.25)	-1.39(-1.51,-1.28)
Tokelau	female	0.01(0.00,0.01)	0.00(0.00,0.01)	-0.450995459	0.16(0.09,0.25)	0.57(0.34,0.90)	-1.53(-1.57,-1.49)
Tuvalu	female	0.04(0.02,0.07)	0.04(0.02,0.06)	-0.050077623	1.09(0.59,1.80)	0.74(0.42,1.16)	-1.32(-1.36,-1.29)
Afghanistan	male	106.16(65.60,151.62)	227.05(146.22,315.11)	1.138792138	2531.13(1518.08,3666.49)	4.35(2.90,5.93)	1.43(1.28,1.57)
Albania	male	63.13(51.28,75.89)	101.57(70.63,140.39)	0.60893573	1567.24(1267.39,1895.85)	4.96(3.45,6.84)	-1.26(-1.52,-0.99)
Algeria	male	160.98(116.02,211.41)	233.59(170.62,315.76)	0.450999774	3566.30(2534.79,4694.59)	1.57(1.15,2.10)	-2.64(-2.76,-2.52)
American Samoa	male	0.55(0.38,0.73)	0.77(0.53,1.03)	0.393723	15.05(10.04,20.72)	3.54(2.47,4.6	-1.48(-1.63,-1

				805		7)	.34)
Andorra	male	1.12(0.79,1.56)	1.53(1.11,2.06)	0.37216324	24.84(17.05,35.15)	2.22(1.62,2.98)	-2.32(-2.38,-2.26)
Angola	male	47.25(29.04,67.57)	71.99(47.65,100.72)	0.523726846	1303.64(770.48,1900.85)	1.63(1.13,2.21)	-2.07(-2.23,-1.91)
Antigua and Barbuda	male	0.70(0.52,0.89)	0.81(0.58,1.08)	0.160024908	14.51(10.81,18.74)	1.86(1.34,2.46)	-1.71(-2.03,-1.40)
Argentina	male	796.24(639.29,948.60)	672.69(528.45,810.56)	-0.155165989	18647.75(14917.52,22287.91)	2.85(2.24,3.43)	-2.29(-2.38,-2.21)
Armenia	male	140.60(117.41,163.15)	111.06(86.63,136.37)	-0.210131213	3963.44(3264.75,4619.63)	6.39(4.96,7.88)	-2.39(-2.58,-2.20)
Australia	male	209.44(170.07,247.39)	129.11(101.34,158.46)	-0.383537856	4721.02(3819.03,5593.30)	0.67(0.52,0.82)	-4.51(-4.67,-4.35)
Austria	male	285.02(231.61,337.89)	135.29(108.78,163.96)	-0.525312254	6072.86(4963.42,7177.37)	1.69(1.36,2.04)	-4.88(-5.10,-4.67)
Azerbaijan	male	285.96(224.83,346.80)	368.09(268.57,487.71)	0.287208392	8121.36(6275.11,9923.06)	9.89(7.41,13.54)	-1.14(-1.44,-0.84)
Bahrain	male	3.04(2.26,3.86)	5.09(3.59,7.02)	0.672826801	73.43(54.11,94.31)	1.52(1.08,2.03)	-4.05(-4.41,-3.68)
Bangladesh	male	926.24(662.51,1211.48)	1032.67(710.30,1470.46)	0.114903198	22964.72(15727.25,30699.26)	1.61(1.11,2.25)	-3.06(-3.34,-2.79)
Barbados	male	4.14(3.16,5.30)	3.33(2.43,4.39)	-0.195729733	80.68(60.99,102.53)	1.54(1.12,2.01)	-3.18(-3.41,-2.94)
Belarus	male	767.33(620.43,909.47)	406.39(300.38,531.68)	-0.470375206	20324.11(16193.43,24247.67)	6.91(5.15,9.04)	-3.62(-3.87,-3.36)
Belgium	male	295.90(242.21,349.2	172.38(138.31,207.26	-0.41745	5814.17(4759.74,6822.20	1.65(1.33,1.9	-3.60(-3.73,-3

		4)	)	0855	)	8)	.48)
Belize	male	1.18(0.87,1.51)	2.47(1.80,3.23)	1.092159776	25.85(18.80,33.30)	1.90(1.39,2.49)	-1.74(-2.34,-1.13)
Benin	male	24.36(15.77,34.04)	28.48(17.83,41.53)	0.168992086	529.06(334.66,755.88)	1.51(0.96,2.15)	-1.53(-1.72,-1.34)
Bermuda	male	0.76(0.54,0.99)	0.68(0.49,0.89)	-0.108318922	17.10(11.89,22.66)	1.17(0.85,1.55)	-2.91(-3.13,-2.70)
Bhutan	male	1.44(0.85,2.12)	2.52(1.56,3.69)	0.748505031	37.88(21.78,57.12)	0.98(0.62,1.41)	-1.23(-1.36,-1.11)
Bolivia (Plurinational State of)	male	134.62(91.47,185.11)	170.44(111.79,246.21)	0.266120008	3112.16(2051.74,4365.99)	4.38(2.89,6.28)	-3.04(-3.39,-2.69)
Bosnia and Herzegovina	male	91.91(73.64,110.61)	122.77(89.63,162.23)	0.335848481	2403.96(1894.50,2901.46)	4.69(3.44,6.12)	-0.69(-0.93,-0.45)
Botswana	male	6.72(4.08,9.87)	10.72(7.08,15.12)	0.59432806	173.35(101.31,261.48)	2.19(1.51,2.99)	-1.92(-2.53,-1.30)
Brazil	male	2991.00(2448.13,3530.82)	2597.17(2026.15,3163.57)	-0.13166973	72515.42(58752.60,86417.49)	2.54(1.98,3.10)	-3.88(-4.09,-3.67)
Brunei Darussalam	male	5.28(4.12,6.64)	4.13(3.09,5.45)	-0.218047619	134.76(105.01,169.24)	4.19(3.16,5.41)	-4.21(-4.58,-3.84)
Bulgaria	male	481.59(390.14,563.95)	287.47(209.38,372.21)	-0.403089233	11984.41(9759.59,14147.95)	4.60(3.37,5.95)	-1.00(-1.42,-0.58)
Burkina Faso	male	35.01(18.53,54.25)	45.16(24.91,69.76)	0.289777453	890.82(457.04,1405.79)	1.19(0.68,1.80)	-1.10(-1.38,-0.82)
Burundi	male	27.87(16.23,41.74)	21.90(11.76,34.46)	-0.21395166	707.72(395.38,1073.70)	1.02(0.58,1.55)	-4.15(-4.41,-3.89)
Cambodia	male	121.22(86.50,165.22	170.45(125.35,215.90	0.406085	3043.95(2151.71,4185.84	4.19(3.15,5.2	-1.75(-1.84,-1

		)	)	209	)	3)	.66)
Cameroon	male	46.18(30.05,65.60)	80.84(48.55,124.45)	0.750475241	1191.34(745.88,1737.96)	1.55(0.98,2.31)	-1.11(-1.28,-0.94)
Canada	male	455.64(376.02,541.09)	373.30(294.54,457.89)	-0.180710564	9850.78(8117.48,11556.83)	1.16(0.91,1.42)	-3.64(-3.78,-3.51)
Cabo Verde	male	4.04(2.66,5.49)	5.29(3.67,7.26)	0.30710269	88.26(57.88,119.45)	3.18(2.21,4.31)	-2.40(-2.98,-1.82)
Central African Republic	male	13.27(7.98,19.73)	14.47(8.41,22.02)	0.09041862	380.23(219.89,575.56)	1.54(0.98,2.24)	-1.94(-2.10,-1.78)
Chad	male	27.96(17.10,40.88)	45.15(26.88,67.50)	0.614906859	636.41(368.38,956.76)	1.71(1.06,2.49)	-0.58(-0.75,-0.41)
Chile	male	403.06(312.78,502.98)	434.66(325.52,546.08)	0.078388217	10250.59(7673.74,12844.57)	4.01(3.01,5.04)	-2.73(-2.88,-2.58)
China	male	56113.73(42264.20,71460.33)	89608.60(67259.22,113081.34)	0.596910478	1422885.60(1051944.92,1841573.05)	9.83(7.43,12.36)	-1.40(-1.84,-0.95)
Colombia	male	517.27(392.54,647.67)	402.74(255.07,579.42)	-0.221410405	12908.10(9451.71,16453.75)	1.69(1.07,2.44)	-4.63(-4.80,-4.46)
Comoros	male	1.86(0.95,2.84)	2.31(1.40,3.45)	0.240733611	42.71(19.32,67.69)	1.18(0.72,1.72)	-1.84(-1.98,-1.69)
Congo	male	9.66(6.01,13.70)	12.40(7.91,18.13)	0.284268915	256.83(156.08,374.03)	1.18(0.81,1.63)	-2.76(-3.05,-2.47)
Costa Rica	male	93.90(73.61,114.25)	89.75(62.10,123.19)	-0.04417985	2062.32(1597.58,2529.99)	3.90(2.69,5.36)	-4.17(-4.49,-3.86)
C 么 te d'Ivoire	male	47.40(30.68,66.06)	93.82(62.22,132.13)	0.97951091	1320.75(839.42,1891.72)	1.90(1.30,2.61)	-0.70(-0.83,-0.57)
Croatia	male	286.70(233.88,337.3	142.92(105.13,186.50	-0.50151	6929.35(5623.50,8157.91	3.81(2.81,4.9	-3.86(-4.01,-3

		4)	)	7148	)	6)	.70)
Cuba	male	138.94(113.15,163.38)	169.64(125.97,219.04)	0.220957838	2938.58(2360.46,3486.67)	1.89(1.40,2.44)	-1.05(-1.16,-0.94)
Cyprus	male	10.58(8.10,13.27)	20.49(15.83,25.70)	0.937019725	228.25(173.65,288.11)	2.24(1.73,2.81)	-0.78(-1.05,-0.51)
Czechia	male	404.29(326.29,477.29)	196.92(143.41,253.80)	-0.512921143	9021.24(7257.25,10642.54)	2.11(1.54,2.71)	-4.33(-4.43,-4.24)
Democratic Republic of the Congo	male	113.13(57.93,178.54)	125.99(65.07,207.36)	0.113651569	2970.53(1452.21,4754.09)	0.87(0.48,1.38)	-2.34(-2.42,-2.25)
Denmark	male	146.67(122.06,171.28)	77.68(62.19,93.24)	-0.470374567	2974.18(2454.74,3478.39)	1.43(1.15,1.72)	-3.48(-3.68,-3.27)
Djibouti	male	1.24(0.66,1.93)	4.96(2.84,7.59)	3.007972948	34.31(17.65,55.03)	1.82(1.13,2.67)	-0.92(-1.07,-0.76)
Dominica	male	1.63(1.19,2.09)	1.25(0.86,1.72)	-0.23484592	34.73(25.27,45.04)	2.88(1.99,3.97)	-2.35(-2.60,-2.09)
Dominican Republic	male	26.62(19.73,34.11)	84.39(57.20,120.28)	2.169868281	598.18(437.37,776.02)	2.01(1.37,2.89)	1.44(1.19,1.68)
Ecuador	male	153.07(110.19,199.34)	218.27(146.67,316.31)	0.42601185	3319.13(2296.12,4464.63)	3.27(2.23,4.66)	-2.36(-2.55,-2.17)
Egypt	male	220.14(169.70,271.60)	500.24(348.54,712.46)	1.272353928	5442.20(4181.97,6780.98)	1.62(1.13,2.27)	-0.24(-0.37,-0.11)
El Salvador	male	28.91(21.07,37.76)	48.12(31.29,69.12)	0.664237084	718.76(512.99,963.69)	1.92(1.24,2.75)	-0.83(-1.15,-0.52)
Equatorial Guinea	male	1.92(0.99,3.10)	1.57(0.86,2.55)	-0.182861709	52.92(26.13,86.27)	0.90(0.51,1.43)	-3.78(-4.28,-3.27)
Eritrea	male	7.49(3.95,12.11)	12.00(6.88,18.59)	0.602926	237.90(120.92,388.71)	1.03(0.62,1.4	-1.99(-2.26,-1

				291		9)	.71)
Estonia	male	85.24(67.89,101.84)	43.22(31.18,58.03)	-0.492982497	2207.00(1755.68,2646.77)	4.38(3.16,5.88)	-3.40(-3.59,-3.20)
Ethiopia	male	103.21(52.23,159.34)	60.44(33.60,94.47)	-0.414443614	2776.84(1342.12,4306.60)	0.32(0.18,0.50)	-4.58(-4.88,-4.28)
Micronesia (Federated States of)	male	1.25(0.79,1.76)	1.40(0.80,2.05)	0.123287995	35.00(21.44,51.56)	4.42(2.88,6.28)	-0.93(-1.04,-0.82)
Fiji	male	3.74(2.52,5.07)	4.65(2.83,6.77)	0.244288308	101.47(64.99,143.07)	1.53(1.00,2.13)	-1.58(-1.71,-1.45)
Finland	male	117.99(94.92,141.69)	62.06(48.44,77.02)	-0.474020742	2637.04(2100.58,3156.76)	1.12(0.88,1.39)	-4.68(-4.92,-4.44)
France	male	1395.11(1141.23,1650.44)	891.48(705.10,1091.36)	-0.360995878	28031.53(22638.95,32997.69)	1.47(1.16,1.79)	-3.70(-3.82,-3.57)
Gabon	male	4.02(2.42,5.93)	4.82(2.91,7.23)	0.196651341	105.11(60.75,160.92)	1.08(0.70,1.55)	-1.56(-1.62,-1.49)
Georgia	male	197.27(155.64,241.71)	139.86(105.30,177.52)	-0.291014565	5559.42(4336.22,6882.15)	5.80(4.42,7.32)	-0.11(-0.60,0.39)
Germany	male	2495.94(2052.20,2944.62)	1784.70(1428.20,2147.59)	-0.284957955	52828.06(43543.19,62161.08)	2.06(1.65,2.48)	-3.34(-3.56,-3.13)
Ghana	male	26.13(15.43,39.77)	27.16(15.63,41.17)	0.039303015	618.40(341.37,975.92)	0.49(0.30,0.72)	-3.24(-3.69,-2.79)
Greece	male	422.30(352.08,493.51)	379.78(308.67,450.51)	-0.100696609	9008.94(7444.17,10487.66)	3.41(2.79,4.02)	-2.41(-2.57,-2.25)
Greenland	male	1.24(0.92,1.55)	1.37(0.97,1.81)	0.104790858	34.86(24.94,44.79)	3.83(2.82,4.95)	-2.55(-2.69,-2.41)
Grenada	male	0.84(0.60,1.10)	0.74(0.53,0.99)	-0.11634	17.87(12.88,23.64)	1.49(1.09,1.9	-1.83(-2.32,-1

				7902		3)	.34)
Guam	male	0.58(0.39,0.79)	0.95(0.64,1.33)	0.644198327	15.49(10.24,21.50)	1.04(0.70,1.44)	-1.60(-1.94,-1.26)
Guatemala	male	61.04(39.32,84.39)	132.65(82.38,192.81)	1.173255791	1432.64(877.02,2057.24)	2.89(1.84,4.17)	-1.38(-1.98,-0.77)
Guinea	male	35.00(21.72,50.01)	70.41(43.95,103.33)	1.011762619	834.86(493.12,1217.68)	2.69(1.72,3.89)	0.91(0.65,1.17)
Guinea-Bissau	male	4.91(2.93,7.23)	4.09(2.50,6.08)	-0.167649304	128.58(74.50,192.56)	1.40(0.90,2.04)	-1.80(-2.24,-1.36)
Guyana	male	4.45(3.19,5.88)	3.71(2.43,5.29)	-0.166017125	114.51(81.35,154.93)	1.30(0.88,1.83)	-2.35(-2.50,-2.21)
Haiti	male	42.82(23.42,65.52)	43.10(22.76,68.67)	0.006678211	1148.73(619.54,1782.09)	1.40(0.76,2.18)	-2.37(-2.44,-2.31)
Honduras	male	27.58(19.77,37.22)	72.55(50.51,99.14)	1.630324137	660.46(454.69,909.03)	2.77(1.94,3.74)	0.09(-0.24,0.43)
Hungary	male	525.15(419.24,615.41)	258.05(194.19,327.03)	-0.508622056	11968.47(9649.54,13988.11)	3.30(2.48,4.16)	-3.40(-3.50,-3.30)
Iceland	male	6.73(5.42,8.14)	3.63(2.79,4.56)	-0.4612629	137.44(112.55,163.67)	1.37(1.05,1.71)	-4.58(-4.71,-4.45)
India	male	4589.74(3226.95,6031.17)	6138.23(4206.80,8270.49)	0.337382533	121382.99(83628.61,161903.44)	1.19(0.84,1.61)	-2.52(-2.68,-2.36)
Indonesia	male	997.88(702.75,1354.56)	1898.55(1313.46,2504.53)	0.902585433	25814.10(17640.11,35629.22)	2.24(1.63,2.89)	0.00(-0.15,0.16)
Iran (Islamic Republic of)	male	844.82(639.11,1051.73)	1359.88(1082.55,1649.03)	0.6096732	21602.83(16314.98,26969.77)	4.04(3.18,4.88)	-1.63(-1.98,-1.28)
Iraq	male	98.65(71.72,128.56)	196.17(142.47,252.22	0.988557	2417.47(1710.98,3255.27	2.02(1.51,2.5	-1.40(-1.51,-1

			)	821	)	5)	.29)
Ireland	male	94.63(77.74,112.13)	55.21(43.81,67.61)	-0.41653 5922	1919.97(1559.47,2274.87)	1.56(1.25,1.9 2)	-4.16(-4.25,-4 .07)
Israel	male	88.54(71.79,105.76)	88.69(70.16,107.22)	0.001729 463	1773.22(1460.15,2109.10)	1.67(1.32,2.0 2)	-3.50(-3.68,-3 .31)
Italy	male	2859.89(2347.10,33 36.60)	1612.78(1289.46,194 5.79)	-0.43606 8338	59397.20(48388.23,6985 3.58)	2.45(1.97,2.9 5)	-4.09(-4.18,-4 .00)
Jamaica	male	30.35(23.22,37.16)	30.79(21.65,41.45)	0.014495 701	646.92(496.15,795.91)	2.20(1.55,2.9 5)	-1.23(-1.68,-0 .78)
Japan	male	11898.78(9940.45,1 3716.19)	8767.37(6995.77,106 23.51)	-0.26317 0332	267070.12(221987.43,30 7333.90)	5.18(4.18,6.2 5)	-4.06(-4.19,-3 .93)
Jordan	male	19.68(15.01,24.98)	53.01(38.15,70.23)	1.693229 391	507.42(383.21,654.62)	1.79(1.31,2.3 8)	-2.49(-2.69,-2 .30)
Kazakhstan	male	705.02(560.33,860.7 1)	397.66(306.14,505.18)	-0.43595 4982	19902.73(15515.60,2448 6.27)	5.82(4.48,7.2 9)	-2.94(-3.13,-2 .76)
Kenya	male	46.47(28.30,68.81)	112.58(70.31,163.49)	1.422506 015	1120.37(645.45,1717.17)	1.26(0.82,1.8 0)	0.16(-0.44,0.7 7)
Kiribati	male	1.23(0.84,1.67)	1.88(1.23,2.68)	0.528345 271	36.03(23.29,49.95)	7.22(5.11,9.5 8)	-0.32(-0.36,-0 .27)
Kuwait	male	5.28(4.11,6.55)	11.47(8.56,15.03)	1.174437 211	136.78(105.97,170.42)	0.94(0.70,1.2 3)	-2.33(-2.70,-1 .96)
Kyrgyzstan	male	194.86(158.75,229.4 7)	148.26(115.78,183.90)	-0.23916 2887	5602.80(4531.51,6675.34)	7.61(6.04,9.3 7)	-2.55(-3.08,-2 .03)
Lao People's Democratic Republic	male	58.41(40.00,79.19)	47.76(33.63,63.93)	-0.18235 0382	1524.24(1044.46,2061.86)	2.74(1.98,3.6 1)	-3.18(-3.38,-2 .98)
Latvia	male	152.32(123.89,179.8	70.20(51.52,91.21)	-0.53914	3963.45(3191.37,4742.27	4.84(3.57,6.2	-3.13(-3.43,-2

		5)		9546	)	9)	.83)
Lebanon	male	34.17(25.85,45.08)	60.81(45.57,86.17)	0.779548158	817.38(606.87,1087.51)	2.60(1.95,3.68)	-0.29(-0.57,-0.01)
Lesotho	male	12.47(8.19,17.36)	16.82(11.98,23.12)	0.34961878	321.19(209.00,450.72)	3.53(2.57,4.72)	0.30(0.18,0.41)
Liberia	male	11.26(6.73,16.63)	9.77(5.45,15.24)	-0.132435501	258.59(151.67,390.42)	1.10(0.64,1.66)	-1.59(-1.89,-1.30)
Libya	male	21.83(15.23,28.76)	40.80(29.08,54.41)	0.86892204	532.40(367.56,716.49)	1.72(1.23,2.29)	-1.42(-1.68,-1.15)
Lithuania	male	177.30(140.69,213.25)	93.98(70.13,121.49)	-0.469909781	4438.98(3479.34,5405.15)	4.44(3.33,5.77)	-2.80(-3.00,-2.60)
Luxembourg	male	10.53(8.35,12.82)	6.68(5.07,8.43)	-0.365947948	224.25(176.44,274.19)	1.46(1.11,1.84)	-4.25(-4.34,-4.15)
North Macedonia	male	84.19(65.43,102.99)	100.61(73.71,133.56)	0.195067975	2133.01(1633.71,2627.18)	6.49(4.78,8.50)	-1.68(-1.87,-1.49)
Madagascar	male	35.75(20.00,54.25)	30.97(14.81,50.76)	-0.133767309	912.88(485.24,1415.98)	0.64(0.35,1.01)	-3.23(-3.55,-2.91)
Malawi	male	12.99(7.92,18.58)	16.98(10.23,24.49)	0.307471827	285.49(164.08,415.24)	0.73(0.47,1.03)	-1.32(-1.59,-1.05)
Malaysia	male	128.03(98.49,158.60)	214.46(155.09,288.02)	0.675037389	3055.23(2304.75,3830.03)	1.80(1.30,2.39)	-2.54(-2.84,-2.25)
Maldives	male	1.16(0.83,1.53)	1.43(1.07,1.85)	0.236305186	28.56(19.84,38.30)	1.06(0.77,1.36)	-3.97(-4.20,-3.73)
Mali	male	34.17(18.59,53.04)	76.40(41.98,118.62)	1.23563669	884.47(451.85,1405.87)	1.90(1.12,2.88)	0.40(0.25,0.56)
Malta	male	8.78(7.08,10.53)	6.70(5.22,8.32)	-0.23674	191.16(152.94,228.07)	1.53(1.19,1.9	-3.90(-4.01,-3

				3249		0)	.79)
Marshall Islands	male	0.35(0.23,0.49)	0.51(0.32,0.74)	0.461999378	9.88(6.50,13.88)	2.99(1.96,4.17)	-1.27(-1.51,-1.03)
Mauritania	male	10.75(6.80,15.37)	11.80(7.33,17.46)	0.097298816	271.02(162.99,388.43)	1.22(0.77,1.79)	-2.15(-2.34,-1.97)
Mauritius	male	14.22(10.57,17.87)	13.07(8.98,17.38)	-0.080837036	342.30(247.15,436.47)	1.76(1.24,2.31)	-3.75(-3.97,-3.53)
Mexico	male	638.48(474.12,798.64)	676.80(451.28,940.47)	0.060019813	13826.30(9814.53,17748.77)	1.32(0.89,1.83)	-3.48(-3.57,-3.40)
Republic of Moldova	male	155.43(123.00,184.54)	103.90(81.25,130.25)	-0.331553567	4458.79(3495.92,5377.44)	4.25(3.34,5.28)	-1.59(-2.05,-1.12)
Mongolia	male	86.32(60.78,115.39)	133.95(91.30,189.18)	0.551667415	2198.08(1504.42,3024.68)	15.57(11.15,20.89)	-1.49(-1.74,-1.24)
Montenegro	male	10.32(7.96,12.89)	15.74(11.63,20.08)	0.524929979	247.51(189.38,309.62)	3.55(2.65,4.52)	-0.31(-0.52,-0.11)
Morocco	male	92.88(65.10,122.34)	145.25(100.31,193.61)	0.563793937	2244.37(1579.66,2958.85)	1.05(0.73,1.41)	-1.64(-1.92,-1.35)
Mozambique	male	27.14(15.26,40.38)	45.00(24.36,69.09)	0.658126843	643.06(345.44,978.81)	1.13(0.66,1.67)	0.29(0.18,0.40)
Myanmar	male	561.07(389.05,791.35)	355.00(255.25,471.88)	-0.36728778	14527.18(9717.58,20601.41)	2.05(1.52,2.65)	-3.75(-3.82,-3.68)
Namibia	male	2.60(1.61,3.85)	2.85(1.70,4.13)	0.095845439	57.15(33.61,86.12)	0.62(0.38,0.87)	-1.63(-1.80,-1.47)
Nepal	male	93.43(55.14,137.38)	154.56(94.17,223.57)	0.654280165	2322.36(1286.35,3489.85)	1.65(1.05,2.33)	-0.77(-0.95,-0.60)
Netherlands	male	554.86(462.81,642.8	329.99(262.16,400.52	-0.40527	11498.15(9614.74,13269.	2.07(1.65,2.5	-4.26(-4.39,-4

		7)	)	0658	25)	1)	.12)
New Zealand	male	54.66(44.14,65.70)	38.01(29.70,47.18)	-0.304628842	1170.48(928.94,1400.91)	1.03(0.81,1.28)	-4.12(-4.26,-3.99)
Nicaragua	male	25.78(18.52,33.29)	47.09(31.93,65.65)	0.827027032	594.69(414.40,792.02)	2.68(1.83,3.64)	-2.05(-2.23,-1.87)
Niger	male	9.99(5.68,15.18)	26.26(14.85,41.12)	1.627134478	266.56(145.21,415.24)	0.86(0.52,1.30)	0.67(0.51,0.84)
Nigeria	male	67.22(38.01,103.29)	84.62(50.40,133.38)	0.258969317	1607.14(859.89,2537.45)	0.24(0.15,0.37)	-1.03(-1.16,-0.91)
Democratic People's Republic of Korea	male	651.11(448.15,894.74)	1241.90(905.08,1622.27)	0.907364094	18577.36(12384.71,26225.05)	9.60(7.23,12.40)	-0.46(-0.74,-0.18)
Northern Mariana Islands	male	0.33(0.22,0.46)	0.59(0.39,0.80)	0.755206158	10.45(6.34,15.04)	2.41(1.71,3.19)	-1.62(-1.87,-1.37)
Norway	male	108.64(86.17,130.18)	37.32(29.24,45.93)	-0.656453504	2160.06(1724.19,2565.47)	0.82(0.65,1.01)	-5.06(-5.32,-4.80)
Oman	male	9.49(6.33,13.24)	7.63(5.48,10.07)	-0.195569802	259.37(169.87,366.95)	1.22(0.86,1.62)	-3.26(-3.42,-3.10)
Pakistan	male	690.31(498.38,911.64)	815.79(550.97,1132.98)	0.181776258	14915.24(10628.68,19870.70)	1.71(1.20,2.33)	-1.08(-1.41,-0.76)
Palestine	male	15.02(10.44,20.27)	22.58(16.52,28.91)	0.503291611	354.65(242.84,484.87)	2.41(1.80,3.06)	-1.83(-1.93,-1.73)
Panama	male	28.63(21.94,35.42)	28.96(19.93,40.28)	0.011234757	645.62(489.83,806.15)	1.47(1.02,2.04)	-3.48(-3.66,-3.29)
Papua New Guinea	male	25.18(14.06,38.16)	51.48(25.76,83.96)	1.043917805	726.25(393.46,1123.42)	2.13(1.16,3.31)	-0.69(-0.83,-0.55)
Paraguay	male	40.26(31.04,50.79)	67.67(46.89,93.03)	0.680609	893.26(675.55,1146.49)	2.78(1.97,3.7	-1.89(-2.41,-1

				549		7)	.37)
Peru	male	127.24(78.60,188.92)	190.16(108.50,295.20)	0.494532355	2943.88(1695.66,4511.14)	1.26(0.71,1.95)	-1.64(-1.89,-1.38)
Philippines	male	341.01(257.10,433.77)	491.28(350.74,678.31)	0.440677626	9167.52(6616.39,11814.38)	1.52(1.10,2.07)	-1.97(-2.13,-1.80)
Poland	male	1841.99(1515.42,2134.49)	1142.38(837.17,1516.21)	-0.379813297	43856.88(36049.41,51273.09)	3.87(2.85,5.14)	-3.69(-3.83,-3.55)
Portugal	male	539.73(432.76,645.89)	340.86(266.33,410.44)	-0.368464498	12270.72(9759.15,14707.79)	3.33(2.62,4.03)	-3.54(-3.61,-3.48)
Puerto Rico	male	41.82(30.66,53.28)	29.47(19.87,40.70)	-0.295470018	840.41(603.88,1075.65)	0.90(0.60,1.24)	-3.86(-4.00,-3.72)
Qatar	male	1.31(0.91,1.79)	4.54(3.01,6.53)	2.475817152	36.55(25.09,50.71)	1.33(0.92,1.87)	-2.73(-3.13,-2.32)
Romania	male	783.29(618.17,932.95)	655.43(483.12,858.05)	-0.163234301	20355.92(15850.13,24380.19)	4.12(3.04,5.40)	-1.52(-1.73,-1.31)
Russian Federation	male	8730.37(6942.03,10380.41)	5496.09(4184.92,6909.27)	-0.370462882	236615.58(188830.87,282136.71)	6.26(4.78,7.80)	-3.02(-3.29,-2.75)
Rwanda	male	28.68(17.10,41.40)	32.10(20.22,46.31)	0.11941869	713.69(409.84,1046.85)	1.59(1.05,2.21)	-2.55(-2.95,-2.15)
Saint Lucia	male	1.81(1.37,2.26)	2.40(1.74,3.16)	0.328255648	41.14(30.76,51.92)	2.46(1.80,3.23)	-2.69(-2.99,-2.39)
Saint Vincent and the Grenadines	male	0.87(0.64,1.14)	1.12(0.78,1.50)	0.279354007	20.06(14.34,26.35)	1.65(1.16,2.19)	-2.09(-2.36,-1.82)
Samoa	male	2.37(1.71,3.05)	2.67(2.00,3.47)	0.128275173	58.33(41.06,77.28)	4.13(3.11,5.30)	-1.21(-1.25,-1.16)
Sao Tome and	male	0.39(0.24,0.59)	0.74(0.45,1.10)	0.875016	8.99(5.27,13.80)	1.68(1.05,2.4	1.02(0.92,1.1

Principe				72		5)	1)
Saudi Arabia	male	36.14(24.03,50.38)	67.55(47.65,91.60)	0.869055109	937.30(606.16,1334.69)	0.73(0.54,0.98)	-1.97(-2.08,-1.86)
Senegal	male	42.76(28.19,59.01)	56.32(36.83,80.42)	0.317038883	1063.04(681.30,1492.62)	1.68(1.13,2.38)	-1.32(-1.52,-1.11)
Serbia	male	254.75(190.20,324.58)	248.52(179.87,332.60)	-0.024456022	6535.09(4892.14,8408.29)	3.35(2.43,4.45)	-1.44(-1.68,-1.20)
Seychelles	male	1.04(0.77,1.35)	1.08(0.79,1.38)	0.035564099	24.11(17.55,31.64)	2.36(1.77,2.96)	-2.51(-2.72,-2.30)
Sierra Leone	male	32.94(20.44,47.28)	31.20(17.60,48.75)	-0.052614329	752.95(443.91,1111.74)	1.96(1.14,2.98)	-1.59(-1.86,-1.31)
Singapore	male	62.65(49.71,76.11)	37.35(28.46,46.79)	-0.403827486	1462.58(1154.53,1790.37)	1.11(0.85,1.39)	-6.58(-6.79,-6.38)
Slovakia	male	190.35(146.78,229.85)	106.52(74.71,141.99)	-0.440379206	4489.62(3487.49,5441.81)	2.81(2.00,3.73)	-3.36(-3.54,-3.17)
Slovenia	male	71.67(51.13,94.66)	54.13(39.35,72.32)	-0.244699748	1761.61(1244.74,2360.26)	2.85(2.08,3.82)	-3.70(-3.89,-3.52)
Solomon Islands	male	5.32(3.31,7.48)	9.06(5.92,12.75)	0.703541639	157.25(95.16,229.60)	5.95(4.07,8.23)	-0.75(-0.81,-0.70)
Somalia	male	22.59(12.17,36.53)	37.71(19.07,59.57)	0.669176411	635.86(321.48,1064.79)	1.49(0.82,2.27)	-1.30(-1.41,-1.18)
South Africa	male	244.28(183.81,312.61)	221.20(163.84,285.20)	-0.094495563	6514.33(4689.18,8574.86)	1.27(0.96,1.61)	-3.37(-3.99,-2.73)
Republic of Korea	male	3347.35(2761.20,3937.63)	2165.30(1707.10,2609.79)	-0.353128891	95633.81(77733.03,112138.26)	5.79(4.62,7.02)	-5.98(-6.25,-5.70)
South Sudan	male	17.01(9.07,26.79)	15.40(7.64,25.35)	-0.09434	417.70(213.84,676.60)	0.87(0.45,1.3	-1.82(-1.91,-1

				2693		8)	.74)
Spain	male	1673.57(1383.97,1948.44)	1135.43(918.70,1366.46)	-0.321551167	36394.04(29983.58,42247.00)	2.66(2.17,3.18)	-3.42(-3.65,-3.18)
Sri Lanka	male	106.16(70.75,142.38)	99.90(59.71,151.65)	-0.058951026	2393.37(1511.78,3323.33)	0.97(0.60,1.42)	-2.45(-2.73,-2.16)
Sudan	male	204.29(127.29,280.80)	345.99(220.97,504.13)	0.693646918	5067.27(3153.58,7067.94)	3.78(2.41,5.45)	-0.48(-0.56,-0.41)
Suriname	male	3.18(2.40,4.05)	5.27(3.87,7.03)	0.658324079	76.93(56.19,99.85)	1.98(1.47,2.63)	-1.30(-1.66,-0.94)
Eswatini	male	1.78(0.97,2.76)	1.85(0.99,2.97)	0.037999166	46.77(24.13,75.30)	0.91(0.53,1.40)	-1.73(-2.08,-1.37)
Sweden	male	209.83(169.29,251.42)	85.06(66.58,104.51)	-0.594613328	3907.17(3160.60,4636.59)	0.82(0.64,1.00)	-4.75(-4.92,-4.59)
Switzerland	male	184.54(150.42,217.97)	97.84(77.65,118.87)	-0.469788727	3775.58(3103.71,4448.52)	1.21(0.96,1.46)	-4.19(-4.48,-3.90)
Syrian Arab Republic	male	51.72(36.70,67.94)	83.25(58.02,114.46)	0.609700698	1287.96(910.56,1712.85)	1.45(1.03,1.97)	-1.55(-1.85,-1.25)
Taiwan (Province of China)	male	556.43(456.40,654.59)	627.60(464.90,833.22)	0.127898393	13820.06(11340.77,16259.05)	3.35(2.48,4.46)	-2.57(-2.70,-2.43)
Tajikistan	male	167.03(132.61,204.14)	130.54(93.60,174.98)	-0.218455948	4654.52(3657.38,5720.67)	6.13(4.49,8.16)	-2.03(-2.37,-1.69)
United Republic of Tanzania	male	108.25(63.69,159.76)	147.22(85.70,217.94)	0.360040289	2674.56(1506.11,4041.09)	1.40(0.85,2.05)	-1.71(-1.91,-1.50)
Thailand	male	704.03(543.82,886.49)	711.77(480.85,999.78)	0.010988269	18401.70(13918.60,23808.23)	1.57(1.08,2.19)	-4.43(-4.73,-4.14)
Bahamas	male	1.60(1.13,2.07)	2.21(1.56,2.99)	0.382439	39.75(27.64,52.33)	1.35(0.96,1.8	-2.39(-2.61,-2

				239		1)	.17)
Gambia	male	2.26(1.46,3.19)	3.83(2.53,5.40)	0.695914423	54.44(34.51,78.93)	0.94(0.64,1.31)	-1.57(-1.68,-1.45)
Timor-Leste	male	3.22(1.87,4.63)	7.67(4.55,10.98)	1.383123792	87.39(48.19,130.89)	2.10(1.27,2.94)	-0.96(-1.25,-0.66)
Togo	male	17.55(11.46,24.40)	30.77(20.88,43.48)	0.752836109	424.27(274.64,604.80)	2.48(1.72,3.41)	-1.04(-1.27,-0.80)
Tonga	male	1.48(1.07,1.93)	1.63(1.17,2.13)	0.101898146	33.76(23.63,44.72)	4.87(3.51,6.34)	-1.03(-1.36,-0.71)
Trinidad and Tobago	male	12.19(9.31,15.08)	8.15(5.54,11.35)	-0.331171838	268.33(204.16,334.07)	0.95(0.65,1.32)	-4.72(-5.09,-4.35)
Tunisia	male	63.34(46.90,82.56)	117.79(81.03,168.16)	0.859768625	1423.10(1026.77,1892.21)	2.08(1.44,2.92)	-1.10(-1.18,-1.02)
Turkey	male	1549.56(1197.20,1971.33)	1764.14(1302.28,2325.63)	0.138476324	42232.39(31910.45,54261.52)	4.32(3.18,5.67)	-2.09(-2.65,-1.53)
Turkmenistan	male	80.15(65.58,93.30)	53.42(38.95,71.14)	-0.333516972	2294.44(1862.12,2684.79)	3.07(2.29,4.04)	-4.33(-4.69,-3.96)
Uganda	male	33.06(17.83,51.38)	52.26(27.38,82.20)	0.580668843	829.30(430.36,1291.34)	0.95(0.53,1.45)	-0.90(-1.29,-0.50)
Ukraine	male	3802.62(3063.96,4506.40)	1884.93(1414.42,2421.77)	-0.504308863	104908.97(83433.33,124880.71)	6.49(4.86,8.33)	-3.59(-3.98,-3.20)
United Arab Emirates	male	5.16(3.46,7.37)	27.88(17.54,40.98)	4.399628116	145.08(94.65,212.69)	1.76(1.25,2.38)	-2.54(-2.93,-2.15)
United Kingdom	male	2104.40(1738.60,2446.23)	1009.49(811.49,1218.63)	-0.520294982	42383.90(34878.98,49169.70)	1.69(1.37,2.03)	-4.48(-4.66,-4.30)
United States of	male	3122.07(2539.34,37	2423.56(1937.11,297	-0.22373	68357.31(56776.32,7963	0.95(0.77,1.1	-3.23(-3.36,-3

America		20.35)	5.13)	4025	8.87)	7)	.10)
Uruguay	male	109.64(87.73,130.98)	78.10(60.81,95.47)	-0.287681502	2475.08(1975.20,2968.02)	3.44(2.69,4.20)	-2.09(-2.17,-2.01)
Uzbekistan	male	278.33(208.21,353.25)	301.87(214.46,397.78)	0.084554493	7804.89(5698.23,9994.54)	3.33(2.46,4.28)	-1.93(-2.11,-1.75)
Vanuatu	male	1.27(0.77,1.87)	2.37(1.36,3.58)	0.865753511	33.31(19.10,51.21)	2.81(1.70,4.12)	-1.09(-1.19,-1.00)
Venezuela (Bolivarian Republic of)	male	234.07(173.54,289.02)	283.62(183.04,401.90)	0.211681923	5475.97(3896.82,6988.04)	2.20(1.43,3.11)	-3.79(-4.08,-3.50)
Viet nam	male	1159.20(816.02,1550.63)	1482.89(1059.65,1962.14)	0.279230979	28732.80(19839.22,39349.39)	4.06(2.95,5.17)	-2.56(-2.85,-2.27)
Virginia	male	66.43(53.07,79.85)	59.48(42.32,79.45)	-0.104656241	1512.64(1229.75,1789.76)	0.92(0.66,1.24)	-3.42(-3.58,-3.25)
Yemen	male	159.42(102.60,229.63)	388.67(266.31,556.82)	1.438013569	4319.63(2761.98,6209.99)	6.57(4.56,9.23)	-0.56(-0.64,-0.48)
Zambia	male	21.22(10.86,32.55)	33.14(17.37,51.41)	0.561894541	510.79(249.48,801.01)	1.21(0.66,1.83)	-1.26(-1.59,-0.92)
Zimbabwe	male	62.88(44.63,81.43)	73.04(50.80,97.88)	0.16145743	1562.09(1076.88,2031.04)	2.85(2.06,3.73)	-0.88(-0.98,-0.78)
Monaco	male	1.34(0.99,1.77)	0.93(0.68,1.21)	-0.309837692	25.90(18.82,33.88)	2.05(1.51,2.66)	-2.66(-2.81,-2.50)
San Marino	male	1.59(1.19,2.04)	1.62(0.99,2.36)	0.018636979	32.11(24.16,41.36)	5.20(3.21,7.64)	-2.62(-2.75,-2.49)
Saint Kitts and Nevis	male	0.39(0.28,0.51)	0.42(0.30,0.56)	0.090041467	8.31(5.90,10.87)	1.41(1.02,1.85)	-2.06(-2.21,-1.92)
Cook Islands	male	0.17(0.12,0.23)	0.18(0.13,0.25)	0.057826	4.31(2.86,5.92)	1.54(1.08,2.0	-2.02(-2.12,-1

				63		8)	.92)
Nauru	male	0.09(0.05,0.13)	0.07(0.04,0.10)	-0.20129644	2.74(1.53,4.26)	3.98(2.55,5.53)	-0.61(-0.93,-0.30)
Niue	male	0.03(0.02,0.04)	0.02(0.01,0.03)	-0.313427259	0.73(0.50,1.02)	2.26(1.59,2.98)	-1.51(-1.60,-1.42)
Palau	male	0.18(0.12,0.25)	0.27(0.18,0.39)	0.496965585	4.99(3.23,7.24)	2.63(1.81,3.65)	-1.22(-1.30,-1.15)
Tokelau	male	0.02(0.01,0.02)	0.01(0.01,0.02)	-0.381127966	0.40(0.27,0.55)	1.72(1.19,2.38)	-1.78(-1.83,-1.73)
Tuvalu	male	0.15(0.10,0.20)	0.14(0.09,0.20)	-0.042819257	3.91(2.55,5.46)	3.06(2.05,4.26)	-1.80(-1.92,-1.69)

Note: ASDR, age-standardized death rate; EAPC, estimated annual percentage change.UI is the uncertainty interval,which reflects the certainty of an estimate based on data availability, study size, and consistency across data sources.

Supplementary table 6. The DALYs and age-standardized DALY rate of gastric cancer attributable to smoking in 1990 and 2019, and its temporal trends from 1990 to 2019

Nation	Sex	DALYs Cases No. (95% UI)		Change in absolute number (%)	Age-standardized DALY rate per 100,000 No.(95% UI)		1990-2019 EAPC No. (95%CI)
		1990	2019		1990	2019	
Afghanistan	both	2746.73(1657.18,3984.42)	6896.30(4226.20,10017.61)	1.510735797	37.47(23.22,53.50)	51.48(33.24,71.42)	1.21(1.08,1.33)
Albania	both	1664.13(1345.23,2015.29)	2350.66(1600.37,3253.03)	0.412548104	77.97(63.01,94.14)	54.89(37.50,75.34)	-1.27(-1.52,-1.02)
Algeria	both	3703.26(2657.68,4903.54)	5033.47(3575.14,6830.91)	0.359199234	32.48(23.71,42.50)	15.55(11.30,20.84)	-2.59(-2.73,-2.45)
American Samoa	both	17.97(12.00,24.49)	24.72(16.23,34.82)	0.376197107	73.50(51.42,97.46)	49.53(33.39,68.59)	-1.39(-1.55,-1.23)
Andorra	both	32.48(22.39,45.76)	42.09(29.89,57.47)	0.295918137	57.39(39.98,80.51)	30.20(21.43,41.23)	-2.29(-2.39,-2.20)
Angola	both	1394.34(838.44,2034.82)	2100.35(1335.47,3054.03)	0.506344247	33.39(20.66,47.65)	17.75(11.70,24.98)	-2.56(-2.72,-2.41)
Antigua and Barbuda	both	16.60(12.40,21.31)	21.37(15.14,28.40)	0.287457169	32.12(23.97,41.40)	20.85(14.79,27.59)	-1.57(-1.79,-1.34)
Argentina	both	24277.13(19162.73,29194.42)	20351.18(15886.48,24636.78)	-0.161713891	73.69(58.27,88.52)	38.36(29.73,46.56)	-2.17(-2.24,-2.10)

Armenia	both	4082.50(3357.19,4780.05)	2692.83(2075.86,3316.74)	-0.340396476	138.11(114.40,160.79)	64.41(49.47,79.38)	-2.90(-3.10,-2.69)
Australia	both	6521.72(5236.03,7809.55)	4144.00(3197.38,5088.70)	-0.36458516	33.36(26.66,39.89)	10.78(8.25,13.32)	-3.95(-4.12,-3.78)
Austria	both	8143.05(6593.14,9725.59)	3699.64(2947.17,4483.18)	-0.545668627	71.45(57.93,85.95)	22.36(17.80,27.12)	-4.32(-4.56,-4.09)
Azerbaijan	both	8217.46(6348.02,10049.47)	9729.77(7055.00,13063.01)	0.184035732	150.70(117.51,183.77)	96.46(70.66,129.02)	-1.63(-1.89,-1.37)
Bahrain	both	84.24(62.44,107.78)	137.24(94.79,191.97)	0.629029813	51.07(38.59,64.87)	15.66(11.26,20.90)	-4.60(-4.92,-4.28)
Bangladesh	both	23663.90(16190.97,31446.08)	24262.28(15773.81,35400.10)	0.025286388	50.42(34.90,66.27)	18.55(12.37,26.97)	-3.41(-3.62,-3.20)
Barbados	both	89.63(66.83,113.71)	74.21(52.52,100.05)	-0.171959488	32.04(23.76,40.53)	15.26(10.78,20.56)	-2.93(-3.11,-2.75)
Belarus	both	23008.98(18142.61,27795.37)	10954.56(7866.69,14680.98)	-0.523900594	175.87(138.58,211.76)	70.17(50.57,94.21)	-3.96(-4.24,-3.67)
Belgium	both	8028.42(6555.12,9531.76)	4326.55(3445.98,5197.71)	-0.46109529	52.57(42.67,62.41)	20.24(16.22,24.29)	-3.20(-3.32,-3.07)
Belize	both	29.60(21.50,38.26)	65.31(45.51,87.57)	1.206206649	32.37(23.62,41.80)	23.45(16.51,30.96)	-1.62(-2.17,-1.06)
Benin	both	575.37(365.77,822.25)	695.44(418.93,1065.88)	0.208671229	29.34(18.72,41.52)	14.88(9.24,22.38)	-1.91(-2.10,-1.71)
Bermuda	both	20.30(13.98,26.90)	15.71(10.95,21.25)	-0.226322901	32.21(22.33,42.68)	12.24(8.49,16.56)	-3.18(-3.40,-2.96)
Bhutan	both	45.52(26.74,67.26)	63.43(39.27,94.21)	0.393479669	17.83(10.77,25.82)	11.48(7.16,16.89)	-1.43(-1.51,-1.35)

Bolivia (Plurinational State of)	both	3705.14(2413.60,5327.52)	4332.50(2631.80,6386.83)	0.16932145	115.97(76.13,165.30)	49.13(30.41,71.84)	-3.22(-3.58,-2.86)
Bosnia and Herzegovina	both	3031.97(2399.47,3650.16)	3483.44(2539.16,4680.76)	0.148905581	69.28(55.16,82.84)	58.76(42.79,78.45)	-0.81(-1.06,-0.55)
Botswana	both	197.68(114.75,298.77)	319.93(198.08,470.51)	0.618474021	33.81(20.31,50.06)	22.67(14.72,32.24)	-2.27(-2.93,-1.61)
Brazil	both	94013.89(74870.15,113021.59)	76576.56(58511.33,94793.65)	-0.185476048	103.48(82.93,124.56)	31.94(24.56,39.41)	-4.06(-4.26,-3.85)
Brunei Darussalam	both	156.79(122.68,194.66)	123.91(91.95,164.85)	-0.209693252	156.37(122.45,194.01)	40.60(30.73,52.15)	-4.85(-5.10,-4.59)
Bulgaria	both	15388.25(12427.87,18133.42)	8354.89(6119.80,10951.52)	-0.457060394	119.05(96.47,140.89)	63.67(46.09,83.91)	-1.30(-1.70,-0.89)
Burkina Faso	both	926.15(473.18,1456.10)	1225.29(630.69,1948.75)	0.322988913	20.24(10.59,31.51)	12.90(6.90,20.21)	-1.37(-1.63,-1.11)
Burundi	both	767.15(426.62,1173.05)	634.43(320.61,1013.54)	-0.173004486	32.10(18.26,48.83)	13.22(6.95,20.60)	-3.80(-4.07,-3.53)
Cambodia	both	3317.32(2355.48,4526.91)	4296.85(3061.22,5681.38)	0.295274326	73.41(53.07,99.41)	36.46(26.66,46.99)	-2.31(-2.38,-2.23)
Cameroon	both	1239.38(774.15,1809.74)	2143.19(1227.69,3352.03)	0.729239575	26.68(17.16,38.30)	17.68(10.44,27.06)	-1.24(-1.43,-1.06)
Canada	both	14080.49(11536.90,16438.73)	10573.28(8292.35,12779.46)	-0.249083088	43.63(35.76,51.08)	15.79(12.43,19.05)	-3.52(-3.62,-3.42)
Cabo Verde	both	104.67(67.06,143.23)	129.27(86.52,180.94)	0.234959237	46.69(29.91,64.48)	30.79(20.70,42.44)	-2.62(-3.09,-2.15)
Central African Republic	both	402.90(233.21,611.54)	457.27(250.79,717.05)	0.134948857	30.89(18.63,46.05)	18.31(10.77,27.75)	-2.06(-2.22,-1.90)

Chad	both	693.07(396.28,1036.82)	1149.40(644.75,1762.23)	0.658417245	24.60(14.39,36.46)	20.99(12.29,31.91)	-0.25(-0.43,-0.08)
Chile	both	13802.08(10131.48,17413.51)	13462.44(9776.55,17247.34)	-0.024607781	133.98(99.76,167.73)	55.82(40.59,71.42)	-2.94(-3.04,-2.85)
China	both	1476331.93(1085512.29,1905742.56)	2108852.15(1567190.43,2700370.03)	0.428440381	166.85(124.30,213.15)	100.89(75.25,128.83)	-1.66(-2.16,-1.16)
Colombia	both	17093.83(12311.51,22125.62)	12009.91(7222.08,17923.91)	-0.297412841	95.82(69.91,123.34)	22.86(13.78,34.12)	-5.00(-5.17,-4.82)
Comoros	both	45.28(20.40,71.66)	56.30(32.13,85.68)	0.243351408	20.53(9.68,32.04)	11.62(6.79,17.50)	-2.15(-2.31,-1.98)
Congo	both	272.76(161.93,401.51)	343.37(201.47,527.16)	0.258856516	24.26(14.82,34.93)	12.58(8.02,18.48)	-2.78(-3.11,-2.44)
Costa Rica	both	2395.15(1839.04,2958.00)	2211.31(1461.02,3145.09)	-0.076754405	138.39(106.35,169.77)	42.98(28.41,61.09)	-4.34(-4.65,-4.02)
C 么 te d'Ivoire	both	1409.21(890.83,2008.25)	2726.58(1718.34,3920.43)	0.934824172	32.64(21.25,45.49)	24.67(16.24,34.54)	-0.90(-1.01,-0.79)
Croatia	both	9180.47(7526.78,10850.99)	3832.19(2801.46,5096.11)	-0.582571542	139.60(114.02,164.93)	46.03(33.39,61.32)	-3.97(-4.17,-3.77)
Cuba	both	3721.45(2995.73,4476.72)	4650.16(3378.12,6007.09)	0.249557841	36.21(29.06,43.60)	24.75(17.96,32.13)	-1.03(-1.13,-0.94)
Cyprus	both	299.97(231.27,374.51)	540.82(417.84,669.66)	0.802929223	36.35(28.24,45.40)	27.64(21.41,34.27)	-0.78(-0.99,-0.56)
Czechia	both	12093.97(9688.71,14501.33)	5418.38(3962.73,7038.72)	-0.551976999	87.75(69.89,104.95)	26.60(19.31,34.45)	-4.12(-4.21,-4.03)
Democratic Republic of the Congo	both	3188.94(1570.75,5080.45)	3694.93(1849.04,6189.90)	0.158670128	19.02(9.75,29.77)	9.66(4.99,15.91)	-2.46(-2.58,-2.34)

Denmark	both	4639.70(3828.93,5455.33)	2145.14(1708.59,2584.26)	-0.537655479	59.53(48.88,69.95)	19.25(15.33,23.21)	-3.66(-3.84,-3.48)
Djibouti	both	36.33(18.78,58.30)	139.20(72.62,219.45)	2.831549901	24.53(13.27,37.73)	22.32(12.71,34.03)	-0.71(-0.87,-0.56)
Dominica	both	42.50(30.54,55.44)	31.37(21.14,44.08)	-0.26202261	61.59(43.98,81.81)	34.84(23.48,49.05)	-2.02(-2.27,-1.76)
Dominican Republic	both	832.26(614.07,1076.61)	2449.77(1642.72,3530.32)	1.943515393	22.76(16.94,29.29)	26.58(17.98,37.90)	1.09(0.87,1.32)
Ecuador	both	3953.95(2726.90,5326.93)	5290.84(3230.77,7796.60)	0.338114136	75.86(53.20,101.38)	35.28(21.84,51.96)	-2.70(-2.88,-2.53)
Egypt	both	5528.64(4244.07,6881.60)	12364.31(8380.37,17882.38)	1.236411288	18.71(14.43,22.99)	19.31(13.39,27.30)	0.18(0.08,0.28)
El Salvador	both	924.85(647.49,1247.14)	1351.58(839.73,1990.88)	0.461411943	30.92(21.87,41.63)	23.26(14.41,34.44)	-1.45(-1.72,-1.18)
Equatorial Guinea	both	55.19(27.17,90.57)	42.27(21.24,71.14)	-0.234130658	25.67(12.89,41.64)	8.67(4.62,14.33)	-4.30(-4.88,-3.71)
Eritrea	both	241.46(122.16,392.36)	383.13(211.81,617.86)	0.586703893	19.66(10.52,31.50)	11.71(6.74,18.10)	-2.02(-2.26,-1.78)
Estonia	both	2792.44(2203.28,3380.85)	1261.03(891.77,1700.84)	-0.548414434	136.23(107.12,164.74)	54.07(38.10,72.93)	-3.44(-3.68,-3.20)
Ethiopia	both	2910.55(1398.25,4515.61)	1503.31(795.52,2375.70)	-0.483495649	13.61(6.84,21.01)	3.69(1.96,5.79)	-5.12(-5.41,-4.83)
Micronesia (Federated States of)	both	43.75(26.44,65.87)	52.00(27.82,79.31)	0.188480834	86.39(53.29,126.85)	66.07(37.88,97.11)	-0.97(-1.12,-0.82)
Fiji	both	126.85(80.65,182.61)	143.78(80.22,218.68)	0.133448071	33.06(22.09,46.05)	18.28(10.60,27.03)	-1.86(-2.08,-1.64)

Finland	both	3866.91(3077.61,4643.80)	1705.68(1310.28,2103.86)	-0.558904218	55.41(43.96,66.61)	15.28(11.70,18.93)	-4.43(-4.65,-4.20)
France	both	34434.22(27602.27,40867.40)	21884.92(17179.93,27026.47)	-0.364442729	43.17(34.51,51.54)	17.86(13.92,22.16)	-3.11(-3.22,-2.99)
Gabon	both	112.59(64.09,171.72)	133.38(75.20,206.14)	0.184690654	19.05(10.90,28.81)	12.01(7.13,18.12)	-1.55(-1.62,-1.49)
Georgia	both	5932.18(4610.76,7287.79)	3642.35(2738.08,4638.57)	-0.386001321	91.27(70.76,112.58)	64.78(48.33,83.30)	-0.36(-0.85,0.15)
Germany	both	77613.53(63357.41,92084.22)	47862.00(38039.90,57612.56)	-0.383329172	63.07(51.14,74.78)	27.69(21.95,33.49)	-2.86(-3.00,-2.72)
Ghana	both	722.47(406.63,1127.89)	771.65(428.37,1179.50)	0.068079924	11.94(7.17,18.03)	5.04(2.94,7.48)	-3.29(-3.66,-2.91)
Greece	both	11210.14(9211.20,13091.23)	9311.50(7674.76,10986.57)	-0.169368144	73.82(60.89,86.50)	44.52(36.55,52.62)	-2.11(-2.26,-1.97)
Greenland	both	47.76(35.44,60.87)	44.06(30.76,58.45)	-0.077422069	126.91(97.38,158.28)	59.90(42.43,78.46)	-2.77(-2.90,-2.64)
Grenada	both	21.87(15.61,29.22)	21.59(15.03,29.11)	-0.01286674	32.08(22.52,42.97)	18.60(12.99,24.84)	-1.61(-1.75,-1.46)
Guam	both	19.19(12.55,26.91)	30.85(19.87,44.77)	0.607492293	23.31(15.72,31.82)	15.98(10.33,23.25)	-1.30(-1.63,-0.97)
Guatemala	both	1843.79(1090.22,2667.10)	3659.80(2052.96,5679.29)	0.984939875	50.93(31.45,71.97)	33.12(18.87,50.64)	-1.72(-2.24,-1.19)
Guinea	both	887.37(527.58,1295.25)	1805.18(1079.25,2658.84)	1.034306074	26.23(15.84,38.05)	32.38(19.41,47.57)	1.03(0.82,1.25)
Guinea-Bissau	both	135.12(78.94,203.01)	116.07(69.38,175.96)	-0.140989378	31.91(18.64,47.44)	15.55(9.51,23.13)	-2.15(-2.63,-1.67)

Guyana	both	134.47(93.99,181.49)	116.52(72.15,174.33)	-0.133437717	34.51(24.54,46.36)	17.28(11.08,25.30)	-2.34(-2.48,-2.20)
Haiti	both	1374.19(731.67,2120.77)	1360.55(690.46,2234.32)	-0.009924817	39.46(21.37,59.54)	18.82(9.82,30.22)	-2.52(-2.58,-2.46)
Honduras	both	792.47(546.02,1088.20)	1908.45(1289.58,2687.85)	1.408229444	37.56(26.23,51.16)	31.70(21.66,44.18)	-0.30(-0.54,-0.06)
Hungary	both	15661.76(12600.61,18395.16)	7785.95(5822.43,9975.83)	-0.502868364	105.50(85.06,124.19)	42.69(31.89,55.29)	-3.18(-3.29,-3.08)
Iceland	both	189.80(153.73,226.76)	90.59(71.31,112.28)	-0.52272265	68.00(55.22,81.24)	16.98(13.29,21.03)	-4.85(-4.93,-4.77)
India	both	134726.21(92009.62,178907.19)	172090.90(113535.03,235576.18)	0.277337973	28.82(20.26,37.55)	14.76(10.06,20.00)	-2.55(-2.70,-2.40)
Indonesia	both	27243.38(18213.56,37689.57)	48355.02(32349.15,65812.77)	0.774927313	26.80(18.82,36.23)	22.12(15.26,29.43)	-0.43(-0.56,-0.29)
Iran (Islamic Republic of)	both	23116.11(17479.69,28550.99)	32992.77(25995.92,40017.07)	0.427262963	83.06(63.21,102.07)	45.26(35.60,54.68)	-1.95(-2.27,-1.63)
Iraq	both	2604.71(1854.35,3476.90)	5314.75(3708.96,7016.28)	1.040439896	32.94(23.72,43.54)	22.55(16.18,29.17)	-1.51(-1.63,-1.40)
Ireland	both	2836.45(2313.38,3369.08)	1561.31(1234.17,1898.69)	-0.449554278	68.53(55.96,81.40)	20.96(16.61,25.55)	-4.19(-4.27,-4.11)
Israel	both	2408.88(1972.93,2900.56)	2318.94(1815.46,2804.97)	-0.037334886	49.70(40.49,59.88)	20.70(16.15,25.18)	-3.57(-3.77,-3.37)
Italy	both	76734.72(62066.51,91238.83)	40235.13(32170.51,48236.04)	-0.475659385	86.96(70.78,103.17)	30.71(24.51,37.02)	-3.85(-3.98,-3.73)
Jamaica	both	755.41(578.67,930.07)	790.22(539.96,1083.08)	0.046087122	43.30(33.17,53.27)	26.87(18.36,36.73)	-1.20(-1.64,-0.76)

Japan	both	309343.36(255085.58,359482.07)	169461.50(136132.54,203140.63)	-0.452189618	178.83(147.67,207.86)	52.95(42.46,63.35)	-4.17(-4.27,-4.08)
Jordan	both	569.33(433.65,720.67)	1461.79(1058.84,1916.33)	1.567572663	41.23(31.50,51.86)	21.64(16.08,28.11)	-2.58(-2.75,-2.42)
Kazakhstan	both	21144.07(16380.89,26240.81)	11100.10(8415.92,14273.60)	-0.475025602	156.35(121.64,192.32)	59.71(45.50,76.72)	-3.16(-3.32,-3.01)
Kenya	both	1233.99(710.87,1872.67)	3167.89(1795.10,4666.52)	1.567180631	15.03(8.93,22.32)	14.15(8.56,20.35)	0.09(-0.51,0.68)
Kiribati	both	42.62(27.97,58.89)	67.09(41.73,96.67)	0.574200308	105.79(71.59,142.96)	89.07(58.95,124.53)	-0.55(-0.60,-0.50)
Kuwait	both	143.87(111.17,178.86)	266.78(190.34,353.11)	0.854234921	23.47(18.11,29.24)	10.74(7.96,14.20)	-2.71(-3.13,-2.28)
Kyrgyzstan	both	5814.48(4671.17,6937.05)	4277.60(3282.08,5404.40)	-0.264319137	185.76(150.21,222.00)	85.63(66.49,107.15)	-2.81(-3.28,-2.34)
Lao People's Democratic Republic	both	1671.74(1135.40,2264.31)	1246.16(833.62,1737.77)	-0.254574338	76.86(52.72,102.99)	28.68(19.70,38.93)	-3.63(-3.83,-3.42)
Latvia	both	4728.82(3742.71,5729.78)	1896.29(1396.72,2449.33)	-0.5989928	133.15(105.31,161.61)	54.12(39.62,70.48)	-3.35(-3.69,-3.01)
Lebanon	both	1053.70(785.83,1396.71)	1765.53(1305.16,2390.73)	0.675543159	44.95(33.65,58.82)	34.00(25.20,46.04)	-0.57(-0.81,-0.32)
Lesotho	both	341.18(224.08,475.50)	500.39(345.07,690.14)	0.466629156	33.69(22.55,46.60)	37.12(26.23,50.43)	0.46(0.33,0.59)
Liberia	both	280.84(163.51,423.16)	258.36(136.05,412.20)	-0.080053083	24.51(14.50,36.59)	13.08(7.20,20.43)	-1.86(-2.20,-1.52)
Libya	both	541.01(375.60,728.66)	1007.84(703.41,1373.41)	0.862901281	28.57(19.96,38.09)	19.34(13.85,26.16)	-1.65(-1.91,-1.38)

Lithuania	both	5104.07(3936.45,6278.15)	2393.70(1736.17,3142.95)	-0.531020905	112.98(87.11,139.03)	46.75(33.44,61.92)	-3.02(-3.28,-2.77)
Luxembourg	both	311.22(241.91,382.74)	174.40(131.85,221.78)	-0.439637176	57.69(44.85,70.63)	17.86(13.49,22.68)	-4.13(-4.23,-4.04)
North Macedonia	both	2695.89(2084.51,3340.64)	2905.33(2122.42,3859.87)	0.077689938	136.63(105.03,168.66)	86.65(63.13,115.48)	-1.99(-2.15,-1.82)
Madagascar	both	981.49(517.46,1515.39)	936.92(424.84,1595.78)	-0.045416667	18.49(10.08,28.39)	7.86(3.81,12.85)	-3.26(-3.55,-2.97)
Malawi	both	314.59(179.70,460.43)	415.30(231.18,625.67)	0.320121045	8.92(5.29,12.75)	6.03(3.54,8.92)	-1.70(-1.99,-1.40)
Malaysia	both	3210.39(2417.70,4036.02)	5032.40(3493.89,6959.29)	0.567532912	34.96(26.42,43.39)	18.89(13.34,25.63)	-2.48(-2.66,-2.30)
Maldives	both	31.42(21.56,41.57)	31.50(23.18,41.27)	0.00241258	36.45(25.83,47.39)	10.79(8.01,13.95)	-4.99(-5.29,-4.69)
Mali	both	953.99(487.04,1516.83)	2037.12(1054.26,3266.05)	1.135363517	21.93(11.69,34.05)	23.32(12.52,36.38)	0.38(0.23,0.53)
Malta	both	241.40(192.91,287.79)	165.22(128.77,202.66)	-0.315582044	55.56(44.39,66.43)	18.74(14.70,22.97)	-3.92(-4.03,-3.81)
Marshall Islands	both	11.04(7.28,15.71)	17.56(10.45,26.28)	0.590890842	62.04(41.82,85.57)	43.96(27.61,63.20)	-1.01(-1.15,-0.86)
Mauritania	both	299.39(179.43,427.38)	305.19(180.37,473.15)	0.019349976	29.05(17.84,41.61)	14.60(8.86,22.36)	-2.20(-2.39,-2.01)
Mauritius	both	365.48(263.79,470.02)	312.08(208.05,428.87)	-0.146095495	48.53(35.41,61.78)	17.30(11.60,23.61)	-3.81(-4.02,-3.60)
Mexico	both	17780.10(12230.48,23575.94)	17660.39(10734.03,25678.12)	-0.006733108	42.57(29.88,56.06)	15.11(9.30,21.82)	-3.67(-3.75,-3.60)

Republic of Moldova	both	4720.29(3713.33,5695.52)	2948.14(2302.41,3681.30)	-0.375432047	100.27(78.79,121.35)	51.12(39.96,63.68)	-1.62(-2.08,-1.15)
Mongolia	both	2375.73(1627.80,3269.16)	3854.31(2437.03,5686.80)	0.622371845	223.30(154.08,305.09)	154.53(103.67,220.05)	-1.88(-2.11,-1.64)
Montenegro	both	327.24(251.98,409.00)	459.86(347.95,579.31)	0.40529181	51.03(39.33,63.34)	45.89(34.84,57.65)	-0.41(-0.69,-0.14)
Morocco	both	2324.52(1631.01,3041.29)	3453.46(2331.82,4711.29)	0.485664186	16.76(11.85,21.88)	10.91(7.45,14.69)	-1.74(-1.94,-1.54)
Mozambique	both	720.00(380.45,1095.06)	1233.67(623.42,1963.43)	0.713441234	12.25(6.69,18.26)	11.40(6.04,17.78)	0.12(-0.01,0.26)
Myanmar	both	17920.82(11914.98,25255.09)	10146.73(6817.61,14043.28)	-0.433801874	75.40(51.31,104.35)	22.14(15.35,30.06)	-4.41(-4.55,-4.26)
Namibia	both	79.12(46.75,118.46)	87.51(48.61,130.04)	0.106066311	11.38(6.92,16.67)	6.67(3.86,9.80)	-1.85(-2.06,-1.65)
Nepal	both	3259.30(1797.21,4872.25)	4915.43(2711.26,7428.43)	0.508124739	34.73(19.93,50.72)	22.63(12.86,33.66)	-1.07(-1.24,-0.91)
Netherlands	both	16293.48(13476.30,18871.86)	9062.04(7159.75,11064.81)	-0.443824118	82.74(68.26,95.63)	26.87(21.23,32.77)	-4.08(-4.24,-3.93)
New Zealand	both	1742.35(1385.71,2077.13)	1103.11(848.71,1382.41)	-0.366883143	44.61(35.30,53.53)	14.83(11.43,18.69)	-3.93(-4.02,-3.84)
Nicaragua	both	670.09(462.12,900.93)	1161.47(748.69,1678.12)	0.733296774	43.89(30.96,58.28)	26.36(17.34,37.32)	-2.29(-2.50,-2.09)
Niger	both	282.43(151.21,437.89)	701.71(367.27,1156.89)	1.484545596	9.62(5.49,14.55)	9.02(5.04,14.28)	0.14(-0.01,0.28)
Nigeria	both	1812.24(990.75,2790.04)	2134.79(1222.19,3432.45)	0.177987259	4.13(2.34,6.30)	2.59(1.53,4.09)	-1.33(-1.51,-1.14)

Democratic People's Republic of Korea	both	19539.27(13175.62,27656.96)	34133.78(24032.88,45338.35)	0.746931931	108.44(75.80,149.56)	102.69(73.08,135.36)	-0.25(-0.51,0.02)
Northern Mariana Islands	both	12.56(7.47,18.28)	18.92(11.86,26.99)	0.506539664	60.45(39.95,82.31)	33.11(21.84,45.78)	-2.20(-2.45,-1.94)
Norway	both	3047.17(2434.14,3663.44)	1103.54(854.99,1370.54)	-0.637847886	47.19(37.47,56.43)	12.16(9.46,15.22)	-4.63(-4.88,-4.38)
Oman	both	271.58(177.63,381.74)	199.59(138.06,269.19)	-0.26508944	38.42(25.76,53.15)	12.46(8.84,16.40)	-3.92(-4.14,-3.70)
Pakistan	both	15981.00(11411.18,21187.29)	20958.86(13723.79,29645.84)	0.311485765	28.52(20.48,37.60)	19.30(12.97,26.66)	-1.28(-1.64,-0.93)
Palestine	both	374.88(258.06,514.78)	598.48(428.59,782.24)	0.596447646	43.01(29.74,58.97)	24.42(17.74,31.39)	-1.92(-2.04,-1.80)
Panama	both	735.68(556.26,926.63)	687.88(459.95,977.06)	-0.064978382	49.59(37.34,62.20)	16.70(11.17,23.79)	-3.79(-3.99,-3.60)
Papua New Guinea	both	891.45(482.32,1350.35)	1869.82(907.11,3107.39)	1.097508114	43.57(24.40,64.89)	35.17(17.97,55.77)	-0.46(-0.63,-0.29)
Paraguay	both	1104.85(836.91,1425.51)	1736.52(1149.04,2466.43)	0.571725675	50.30(38.20,64.50)	31.57(21.18,44.56)	-2.16(-2.70,-1.62)
Peru	both	3449.51(1965.52,5338.33)	4717.90(2447.93,7575.43)	0.36770098	28.96(16.94,44.49)	14.86(7.73,23.84)	-1.86(-2.13,-1.59)
Philippines	both	10256.61(7423.73,13220.93)	14270.58(9961.65,20072.72)	0.391354982	32.76(24.29,41.56)	17.68(12.70,24.48)	-2.32(-2.49,-2.16)
Poland	both	53322.65(43318.41,62875.55)	32534.86(24504.43,41967.05)	-0.389849192	120.38(97.81,142.02)	47.83(35.84,61.83)	-3.44(-3.56,-3.31)
Portugal	both	14350.87(11351.88,17323.19)	8355.69(6466.37,10162.38)	-0.417757199	103.72(82.02,125.79)	40.88(31.36,50.51)	-3.30(-3.36,-3.24)

Puerto Rico	both	1017.93(737.88,1311.76)	698.44(456.09,981.31)	-0.313859122	27.88(20.11,35.89)	10.18(6.63,14.43)	-3.81(-3.96,-3.66)
Qatar	both	37.85(26.23,52.21)	127.79(81.60,187.43)	2.375744329	34.74(24.80,47.79)	16.98(11.89,23.66)	-2.74(-3.13,-2.35)
Romania	both	23924.26(18721.80,28949.65)	17608.57(12817.71,23075.13)	-0.263987152	81.81(64.11,99.08)	51.40(37.08,67.40)	-1.81(-2.00,-1.61)
Russian Federation	both	258294.64(203582.82,309777.29)	151305.37(115255.15,191303.11)	-0.414214056	137.58(109.08,165.06)	65.01(49.22,82.24)	-3.03(-3.36,-2.71)
Rwanda	both	854.02(494.91,1261.26)	1011.46(613.53,1485.46)	0.184355765	29.38(17.26,42.92)	17.53(10.89,25.06)	-2.90(-3.32,-2.47)
Saint Lucia	both	47.80(35.40,60.58)	61.30(43.71,82.06)	0.28246121	54.63(40.47,69.32)	28.06(20.03,37.67)	-2.44(-2.70,-2.17)
Saint Vincent and the Grenadines	both	23.26(16.37,30.51)	28.91(19.26,39.49)	0.242677597	32.73(23.00,43.07)	20.89(14.04,28.44)	-1.76(-2.03,-1.49)
Samoa	both	68.41(48.02,90.66)	78.83(56.45,106.58)	0.152253809	76.25(53.51,99.81)	53.61(39.34,71.29)	-1.16(-1.20,-1.11)
Sao Tome and Principe	both	10.06(5.79,15.46)	19.24(11.56,29.06)	0.912512805	15.16(8.93,23.06)	19.00(11.72,28.03)	1.03(0.95,1.12)
Saudi Arabia	both	976.35(635.73,1388.91)	2055.10(1428.25,2904.01)	1.104876245	15.99(10.62,22.43)	10.02(7.24,13.47)	-1.78(-1.86,-1.70)
Senegal	both	1095.46(702.31,1542.45)	1403.53(877.06,2070.47)	0.28122261	32.90(21.26,45.82)	18.51(11.80,26.72)	-1.65(-1.88,-1.43)
Serbia	both	8526.28(6409.06,10812.24)	7210.74(5175.97,9606.29)	-0.154292655	70.35(53.25,88.58)	46.60(33.15,62.35)	-1.80(-2.06,-1.54)
Seychelles	both	25.41(18.47,33.27)	27.31(19.47,35.99)	0.074696948	45.40(32.81,59.33)	24.06(17.38,31.18)	-2.57(-2.77,-2.38)

Sierra Leone	both	830.70(478.80,1242.72)	836.23(429.96,1343.43)	0.006657954	43.28(25.08,64.21)	23.45(12.27,36.98)	-1.68(-1.99,-1.36)
Singapore	both	1682.56(1320.46,2062.10)	902.13(684.47,1132.31)	-0.463835146	75.23(59.35,92.17)	11.50(8.78,14.39)	-6.72(-6.96,-6.47)
Slovakia	both	5445.12(4249.08,6640.07)	2898.52(1980.01,3922.03)	-0.467684162	90.73(70.66,110.56)	31.57(21.53,42.91)	-3.54(-3.74,-3.35)
Slovenia	both	2405.63(1689.20,3244.96)	1495.56(1097.19,2017.08)	-0.378310907	97.87(68.69,132.79)	37.45(27.61,50.47)	-3.72(-3.91,-3.52)
Solomon Islands	both	180.34(108.14,262.92)	322.48(190.03,477.20)	0.788165465	117.65(74.27,166.49)	91.01(58.47,130.00)	-0.98(-1.05,-0.92)
Somalia	both	672.32(335.27,1123.49)	1149.37(529.01,1869.15)	0.709566082	25.06(13.60,40.38)	16.07(7.88,25.19)	-1.59(-1.67,-1.50)
South Africa	both	8133.20(5818.59,10545.04)	6874.89(4875.37,9032.72)	-0.154712835	37.27(27.25,48.00)	14.84(10.74,19.35)	-3.58(-4.13,-3.01)
Republic of Korea	both	103181.92(83346.62,121807.44)	49998.48(38973.13,60847.22)	-0.515433687	307.39(251.17,362.76)	55.25(43.25,67.48)	-6.43(-6.67,-6.19)
South Sudan	both	442.09(225.13,718.87)	407.13(187.36,692.20)	-0.079073335	18.43(9.64,29.16)	10.65(5.21,17.51)	-2.05(-2.15,-1.95)
Spain	both	42904.65(35281.25,50111.22)	28338.86(22851.71,33833.77)	-0.339492213	80.65(66.10,93.85)	33.44(26.89,39.92)	-3.03(-3.31,-2.76)
Sri Lanka	both	2576.86(1635.24,3605.30)	2376.46(1364.51,3756.14)	-0.077770122	24.41(16.06,33.26)	9.07(5.30,14.22)	-3.05(-3.35,-2.75)
Sudan	both	5306.26(3332.99,7380.87)	8713.68(5417.45,12734.04)	0.642152026	55.63(35.11,76.83)	46.73(30.12,67.93)	-0.49(-0.60,-0.38)
Suriname	both	90.46(65.79,120.12)	148.73(104.14,202.79)	0.644028116	34.28(25.22,44.94)	23.99(16.86,32.40)	-1.50(-1.83,-1.17)

Eswatini	both	54.02(28.73,85.03)	58.43(30.35,95.25)	0.081707931	18.55(10.63,28.59)	10.09(5.57,15.96)	-1.83(-2.29,-1.37)
Sweden	both	5936.71(4801.48,7057.51)	2473.43(1932.12,3021.30)	-0.583366331	40.64(32.50,48.27)	12.17(9.54,14.80)	-4.21(-4.36,-4.06)
Switzerland	both	4940.98(4010.02,5884.82)	2543.16(2033.74,3117.88)	-0.485292663	49.71(40.37,59.21)	15.52(12.30,19.06)	-3.83(-4.10,-3.56)
Syrian Arab Republic	both	1402.24(991.72,1867.93)	2146.20(1478.69,3008.80)	0.530554889	26.07(18.51,34.11)	16.96(11.83,23.79)	-1.80(-2.08,-1.53)
Taiwan (Province of China)	both	14396.72(11742.79,16937.94)	13996.65(10231.14,19272.94)	-0.027789254	85.13(69.82,99.92)	34.96(25.46,48.29)	-3.17(-3.32,-3.01)
Tajikistan	both	4817.29(3780.94,5891.36)	3770.54(2692.08,5049.21)	-0.217289095	164.79(129.79,201.55)	69.38(50.33,91.67)	-2.45(-2.72,-2.18)
United Republic of Tanzania	both	3150.24(1711.80,4813.14)	4514.30(2439.84,6928.83)	0.433000933	28.15(15.86,42.25)	18.20(10.11,27.19)	-1.57(-1.71,-1.43)
Thailand	both	19884.36(14803.49,25754.27)	17117.34(10898.95,24986.66)	-0.139155729	53.14(40.13,67.77)	16.46(10.52,23.84)	-4.74(-5.06,-4.42)
Bahamas	both	47.37(32.93,62.02)	64.36(44.00,88.98)	0.35873625	30.41(21.21,39.91)	15.97(11.15,21.99)	-2.30(-2.51,-2.10)
Gambia	both	55.80(35.27,80.84)	90.15(57.38,130.61)	0.615604318	15.88(10.17,22.71)	9.60(6.25,13.73)	-1.85(-2.00,-1.70)
Timor-Leste	both	94.47(52.81,142.38)	187.78(104.89,276.04)	0.987745767	32.44(18.93,46.41)	22.99(13.22,33.46)	-1.38(-1.70,-1.06)
Togo	both	468.05(303.75,660.09)	862.73(566.98,1234.42)	0.843218591	38.48(25.31,53.62)	23.73(16.12,33.36)	-1.62(-1.82,-1.43)
Tonga	both	37.83(26.55,50.14)	38.72(26.51,52.23)	0.023513514	68.86(48.86,89.78)	49.36(34.12,65.90)	-1.21(-1.48,-0.93)

Trinidad and Tobago	both	315.55(238.66,394.97)	207.08(134.50,297.03)	-0.343760296	37.38(28.39,46.74)	10.91(7.16,15.50)	-4.78(-5.15,-4.41)
Tunisia	both	1482.69(1070.91,1951.66)	2613.47(1787.44,3810.91)	0.762655287	29.39(21.47,38.47)	20.61(14.18,29.75)	-1.43(-1.51,-1.34)
Turkey	both	47468.66(36452.14,60242.16)	48616.82(35406.51,64262.05)	0.024187606	122.94(94.95,155.65)	53.33(39.03,70.50)	-2.52(-3.15,-1.89)
Turkmenistan	both	2432.19(1973.49,2857.49)	1597.28(1145.20,2162.82)	-0.343274981	116.54(95.09,136.66)	36.64(26.59,48.74)	-4.30(-4.67,-3.93)
Uganda	both	909.05(465.60,1431.55)	1566.69(786.09,2527.40)	0.723444463	13.85(7.18,21.51)	10.82(5.56,17.27)	-1.12(-1.52,-0.72)
Ukraine	both	113975.16(90129.17,136917.92)	53357.07(39745.33,68794.85)	-0.531853501	159.47(125.56,190.51)	74.18(54.73,96.06)	-3.69(-4.18,-3.20)
United Arab Emirates	both	156.73(104.31,226.30)	913.15(552.33,1362.95)	4.826123364	40.62(28.19,55.94)	23.80(16.43,32.72)	-2.05(-2.37,-1.73)
United Kingdom	both	61991.38(50721.75,72176.61)	27309.20(21970.71,32749.39)	-0.55946779	69.01(56.60,80.49)	22.53(18.14,26.82)	-4.21(-4.40,-4.01)
United States of America	both	99997.03(82877.16,116861.61)	74903.37(60996.82,89862.91)	-0.250944063	32.33(26.91,37.71)	14.00(11.45,16.72)	-3.00(-3.10,-2.89)
Uruguay	both	3058.49(2417.75,3688.23)	2170.28(1685.58,2665.43)	-0.290409127	79.23(62.57,95.18)	43.73(33.66,53.50)	-1.94(-2.02,-1.87)
Uzbekistan	both	8076.28(5892.47,10373.73)	9049.21(6291.53,12183.11)	0.120468263	67.62(49.62,86.38)	36.70(26.30,48.28)	-2.20(-2.40,-2.01)
Vanuatu	both	35.18(20.04,54.33)	66.15(35.12,104.56)	0.880019234	51.30(30.77,77.30)	36.43(20.40,56.56)	-1.27(-1.39,-1.16)
Venezuela (Bolivarian Republic of)	both	7108.00(4988.26,9297.56)	8161.71(4999.90,12114.97)	0.148243313	73.58(52.95,94.97)	27.66(17.03,40.46)	-3.81(-4.05,-3.58)

Viet nam	both	29782.23(20546.73,40769.57)	39088.11(27081.12,53270.60)	0.31246428	72.39(50.09,98.07)	39.49(27.71,53.09)	-2.60(-2.85,-2.35)
Virginia	both	2173.47(1760.09,2564.70)	1852.02(1395.67,2380.51)	-0.147896693	31.02(25.14,36.55)	13.32(10.01,16.98)	-3.13(-3.27,-2.99)
Yemen	both	4844.35(3118.83,6889.71)	11064.62(7561.88,15837.09)	1.284025833	92.08(60.46,129.62)	81.19(55.79,115.29)	-0.57(-0.65,-0.48)
Zambia	both	569.64(275.91,892.01)	955.30(477.77,1530.83)	0.677020388	20.60(10.48,31.84)	14.47(7.60,22.67)	-1.58(-1.93,-1.24)
Zimbabwe	both	1763.86(1208.16,2310.73)	2172.16(1448.87,2998.84)	0.231481132	42.98(30.31,55.86)	30.98(21.21,41.85)	-1.04(-1.12,-0.97)
Monaco	both	34.95(25.03,46.28)	22.46(16.37,29.57)	-0.357449823	54.50(39.15,72.56)	25.83(18.73,34.40)	-2.58(-2.77,-2.38)
San Marino	both	42.02(31.89,54.06)	37.36(22.83,56.99)	-0.110763658	126.99(95.54,163.65)	61.73(36.91,95.26)	-2.52(-2.66,-2.39)
Saint Kitts and Nevis	both	9.93(7.09,13.04)	11.87(8.14,16.15)	0.195196959	27.74(19.86,36.76)	16.73(11.77,22.43)	-2.07(-2.28,-1.85)
Cook Islands	both	5.02(3.39,6.92)	4.86(3.22,6.76)	-0.03139962	38.54(26.23,52.21)	19.83(13.09,27.77)	-2.26(-2.42,-2.09)
Nauru	both	3.58(1.95,5.52)	3.21(1.75,4.97)	-0.102384483	80.65(49.75,119.99)	62.37(37.51,90.89)	-0.85(-1.27,-0.44)
Niue	both	0.90(0.61,1.25)	0.60(0.40,0.83)	-0.334580324	42.78(28.79,59.73)	27.58(18.42,38.33)	-1.70(-1.81,-1.60)
Palau	both	5.31(3.41,7.72)	8.23(5.15,12.18)	0.550905731	50.06(32.95,71.55)	34.23(21.83,49.35)	-1.23(-1.30,-1.15)
Tokelau	both	0.56(0.36,0.77)	0.35(0.22,0.50)	-0.37524587	41.15(26.54,57.94)	25.74(16.48,37.02)	-1.61(-1.64,-1.58)

Tuvalu	both	4.99(3.24,6.93)	4.58(2.82,6.70)	-0.081346316	67.71(44.60,93.71)	43.21(26.94,63.23)	-1.51(-1.63,-1.39)
Afghanistan	female	215.59(96.61,351.98)	732.18(352.61,1232.08)	2.396172321	5.82(2.73,9.24)	9.86(5.13,15.87)	1.97(1.87,2.07)
Albania	female	96.88(62.28,142.37)	161.42(103.22,235.76)	0.666097133	9.19(5.90,13.52)	7.14(4.54,10.44)	-1.57(-1.93,-1.21)
Algeria	female	136.96(85.45,212.76)	177.50(110.63,269.29)	0.296019149	2.48(1.57,3.72)	1.17(0.76,1.74)	-2.54(-2.59,-2.49)
American Samoa	female	2.92(1.66,4.37)	5.17(3.06,7.70)	0.770950718	24.66(14.52,36.48)	20.29(11.95,29.83)	-0.50(-0.73,-0.26)
Andorra	female	7.63(4.86,11.57)	11.44(7.28,16.68)	0.498608482	27.95(18.10,41.98)	16.72(10.57,24.36)	-1.84(-2.13,-1.55)
Angola	female	90.70(43.86,158.69)	162.45(83.60,272.60)	0.791086587	4.34(2.17,7.50)	2.53(1.36,4.22)	-2.23(-2.43,-2.04)
Antigua and Barbuda	female	2.09(1.43,2.86)	3.85(2.51,5.38)	0.844232894	7.08(4.84,9.84)	7.16(4.74,10.01)	-0.35(-0.58,-0.12)
Argentina	female	5629.38(4151.77,7247.86)	5264.53(3782.45,6694.51)	-0.064810947	31.30(23.06,40.44)	18.00(12.82,23.06)	-1.70(-1.80,-1.60)
Armenia	female	119.06(67.24,185.73)	74.66(44.64,111.11)	-0.372969544	7.47(4.25,11.44)	3.03(1.81,4.51)	-3.18(-3.41,-2.94)
Australia	female	1800.70(1397.74,2220.87)	1270.11(944.26,1606.40)	-0.294655077	17.09(13.19,21.09)	6.28(4.63,7.98)	-3.54(-3.73,-3.34)
Austria	female	2070.19(1572.40,2604.08)	1044.66(805.05,1296.74)	-0.495379799	30.57(23.27,38.37)	11.94(9.05,14.90)	-3.43(-3.62,-3.25)
Azerbaijan	female	96.11(47.82,156.72)	142.28(66.17,250.27)	0.480402184	3.17(1.61,5.16)	2.48(1.22,4.15)	-1.01(-1.18,-0.84)

Bahrain	female	10.81(6.90,16.12)	11.04(6.83,16.62)	0.021786316	14.43(9.18,21.18)	3.51(2.25,5.17)	-5.40(-5.68,-5.12)
Bangladesh	female	699.18(342.61,1170.47)	1021.37(463.93,1871.63)	0.460805725	3.59(1.80,6.11)	1.70(0.79,3.11)	-2.45(-2.75,-2.16)
Barbados	female	8.95(6.07,12.61)	9.24(5.84,13.58)	0.032982339	6.20(4.12,8.89)	3.55(2.20,5.23)	-1.64(-1.80,-1.47)
Belarus	female	2684.87(1655.96,3891.93)	1132.72(679.32,1739.42)	-0.578110607	35.81(21.62,52.50)	13.61(8.03,20.94)	-4.26(-4.57,-3.96)
Belgium	female	2214.24(1691.58,2739.10)	1120.95(874.91,1370.84)	-0.493754469	25.18(19.15,31.08)	9.79(7.60,12.01)	-3.24(-3.46,-3.02)
Belize	female	3.75(2.43,5.33)	7.03(4.32,10.41)	0.875701652	8.23(5.31,11.74)	5.21(3.26,7.49)	-1.58(-1.88,-1.27)
Benin	female	46.31(22.58,80.07)	63.38(31.71,102.32)	0.368633815	4.54(2.21,7.81)	2.54(1.33,4.08)	-1.26(-1.53,-0.98)
Bermuda	female	3.20(2.00,4.57)	2.29(1.48,3.33)	-0.283533817	9.06(5.63,12.88)	3.16(2.04,4.58)	-4.21(-4.54,-3.88)
Bhutan	female	7.65(3.80,13.19)	10.48(5.62,17.96)	0.37095755	5.84(3.02,9.90)	3.80(2.06,6.35)	-1.64(-1.70,-1.58)
Bolivia (Plurinational State of)	female	592.98(251.39,1043.43)	688.28(287.16,1197.33)	0.160715698	34.47(14.67,60.54)	14.84(6.30,25.80)	-3.17(-3.50,-2.84)
Bosnia and Herzegovina	female	628.01(450.89,820.88)	769.31(534.02,1053.25)	0.22500149	26.43(19.10,34.62)	24.32(16.68,33.31)	-0.47(-0.72,-0.22)
Botswana	female	24.33(11.78,42.42)	38.89(20.58,63.76)	0.598309773	7.96(4.10,13.62)	5.37(2.94,8.62)	-1.46(-1.72,-1.21)
Brazil	female	21498.46(16150.42,26919.71)	18821.56(13806.40,24116.35)	-0.124515861	44.93(33.67,55.86)	14.37(10.56,18.41)	-3.94(-4.14,-3.74)

Brunei Darussalam	female	22.04(15.10,31.61)	18.36(12.53,25.94)	-0.166697493	43.14(29.94,61.30)	12.12(8.42,16.84)	-4.82(-5.09,-4.55)
Bulgaria	female	3403.85(2477.74,4476.96)	1668.47(1141.56,2288.24)	-0.509826884	50.65(36.86,65.97)	25.22(16.96,35.43)	-1.67(-2.06,-1.27)
Burkina Faso	female	35.33(14.13,65.39)	56.25(22.92,100.76)	0.592046141	1.55(0.64,2.84)	1.11(0.46,1.97)	-0.69(-0.94,-0.45)
Burundi	female	59.43(22.77,115.75)	58.77(21.94,111.06)	-0.010997117	4.56(1.78,8.82)	2.45(0.94,4.51)	-2.93(-3.24,-2.62)
Cambodia	female	273.37(144.56,433.90)	349.58(190.80,559.30)	0.278775743	11.04(6.06,17.34)	5.32(3.05,8.36)	-2.36(-2.48,-2.24)
Cameroon	female	48.04(22.97,84.31)	77.14(34.91,140.82)	0.605550104	2.21(1.08,3.75)	1.36(0.65,2.43)	-1.24(-1.38,-1.09)
Canada	female	4229.71(3381.08,5059.16)	3193.05(2455.83,3952.68)	-0.245089527	23.86(19.05,28.48)	9.02(6.89,11.16)	-3.45(-3.52,-3.37)
Cabo Verde	female	16.41(7.91,26.70)	9.95(5.29,15.71)	-0.393405352	12.71(6.06,20.89)	4.38(2.32,6.97)	-3.61(-4.23,-2.99)
Central African Republic	female	22.66(10.22,41.20)	24.67(10.76,47.56)	0.088427913	3.41(1.59,6.04)	2.01(0.92,3.75)	-2.01(-2.23,-1.78)
Chad	female	56.66(25.16,100.53)	86.04(38.13,154.29)	0.518458818	4.01(1.79,7.05)	3.63(1.67,6.48)	0.40(0.13,0.68)
Chile	female	3551.49(2449.39,4880.96)	3509.85(2240.21,4806.96)	-0.011723257	63.62(44.17,87.24)	27.03(17.00,37.11)	-2.91(-2.99,-2.83)
China	female	53446.33(31832.16,78788.99)	74582.25(49572.01,104061.24)	0.3954606	12.85(7.78,18.75)	7.21(4.85,10.03)	-2.21(-2.99,-1.43)
Colombia	female	4185.73(2571.02,5805.30)	2837.12(1649.91,4337.91)	-0.322192911	47.07(29.62,65.01)	9.95(5.79,15.31)	-5.55(-5.90,-5.21)

Comoros	female	2.57(1.04,4.56)	4.33(2.13,7.40)	0.681565514	2.27(0.97,3.99)	1.66(0.81,2.79)	-1.19(-1.35,-1.02)
Congo	female	15.94(7.15,28.66)	19.93(8.81,35.54)	0.250359758	2.53(1.18,4.46)	1.45(0.67,2.50)	-2.05(-2.21,-1.89)
Costa Rica	female	332.84(211.30,470.06)	348.13(208.84,518.94)	0.045947127	36.80(23.79,51.89)	12.58(7.60,18.80)	-3.87(-4.12,-3.62)
C 么 te d'Ivoire	female	88.47(48.22,141.88)	221.92(123.51,358.43)	1.508589329	5.37(3.02,8.55)	4.99(2.82,7.98)	0.62(0.32,0.92)
Croatia	female	2251.12(1691.54,2829.83)	898.99(645.35,1219.00)	-0.60064578	59.76(45.30,74.86)	19.74(13.72,26.91)	-4.00(-4.26,-3.74)
Cuba	female	782.87(559.60,1021.54)	1020.40(696.34,1405.89)	0.303409188	15.07(10.71,19.73)	10.40(7.06,14.36)	-0.88(-1.03,-0.74)
Cyprus	female	71.72(49.41,98.57)	119.33(86.84,156.05)	0.663807604	16.48(11.35,22.15)	11.77(8.57,15.40)	-0.96(-1.16,-0.76)
Czechia	female	3072.73(2251.80,3934.83)	1356.74(966.31,1813.32)	-0.558456869	38.40(28.13,49.21)	12.36(8.62,16.67)	-4.06(-4.16,-3.96)
Democratic Republic of the Congo	female	218.41(98.68,388.58)	248.48(111.88,456.84)	0.137676231	2.38(1.11,4.21)	1.25(0.57,2.27)	-2.24(-2.28,-2.20)
Denmark	female	1665.52(1359.25,1993.40)	674.31(527.43,829.57)	-0.59513594	38.21(30.70,46.11)	11.39(8.85,14.02)	-4.04(-4.19,-3.90)
Djibouti	female	2.02(0.92,3.77)	7.23(3.15,13.40)	2.584330495	3.06(1.47,5.47)	2.85(1.34,5.15)	-0.55(-0.68,-0.41)
Dominica	female	7.77(4.97,11.10)	4.55(2.97,6.77)	-0.414371522	19.30(11.95,28.21)	9.93(6.37,14.78)	-2.17(-2.39,-1.95)
Dominican Republic	female	234.08(163.15,322.31)	603.88(406.25,863.35)	1.579728464	12.98(9.17,17.79)	12.74(8.59,18.17)	0.46(0.14,0.79)

Ecuador	female	634.82(367.65,935.82)	862.44(488.50,1324.11)	0.358561669	24.09(14.40,35.41)	11.03(6.39,16.94)	-2.58(-2.82,-2.34)
Egypt	female	86.44(55.59,126.43)	166.16(96.27,268.57)	0.922289888	0.66(0.43,0.97)	0.69(0.41,1.09)	0.51(0.36,0.66)
El Salvador	female	206.08(133.41,297.11)	277.13(165.46,431.12)	0.344743654	13.06(8.45,18.85)	8.27(4.93,12.90)	-1.97(-2.21,-1.73)
Equatorial Guinea	female	2.26(0.91,4.31)	2.66(1.11,5.18)	0.175662425	1.96(0.81,3.73)	0.97(0.42,1.83)	-2.57(-2.67,-2.46)
Eritrea	female	3.56(1.30,6.73)	7.00(2.81,13.00)	0.965839213	0.58(0.22,1.11)	0.45(0.19,0.82)	-0.84(-1.03,-0.65)
Estonia	female	585.44(394.97,791.52)	291.53(190.94,409.49)	-0.502028758	47.18(31.59,63.45)	21.27(13.86,30.61)	-3.31(-3.74,-2.88)
Ethiopia	female	133.71(48.07,264.04)	103.64(39.74,200.39)	-0.224861925	1.26(0.46,2.49)	0.49(0.19,0.95)	-3.92(-4.18,-3.66)
Micronesia (Federated States of)	female	8.76(4.43,15.01)	10.93(5.48,18.30)	0.248817858	35.22(18.35,59.77)	28.55(14.90,46.43)	-0.75(-0.93,-0.56)
Fiji	female	25.39(12.86,42.10)	23.43(11.23,39.75)	-0.077291167	13.16(7.13,21.12)	5.80(2.79,9.79)	-2.53(-2.76,-2.31)
Finland	female	1229.87(921.49,1550.36)	497.58(371.05,624.86)	-0.595421247	30.77(23.16,38.64)	8.51(6.27,11.03)	-4.47(-4.74,-4.21)
France	female	6402.69(4807.37,8097.25)	4971.65(3734.49,6312.33)	-0.223505528	14.02(10.46,17.69)	7.48(5.49,9.69)	-2.36(-2.53,-2.19)
Gabon	female	7.48(3.16,13.47)	6.78(3.07,11.98)	-0.093883247	2.36(1.01,4.30)	1.22(0.56,2.13)	-2.37(-2.60,-2.14)
Georgia	female	372.77(218.67,563.53)	209.80(130.38,299.30)	-0.437174232	9.99(5.96,14.92)	6.48(3.90,9.41)	-0.83(-1.38,-0.27)

Germany	female	24785.47(19132.44,30656.39)	13493.55(10432.60,16601.49)	-0.455586355	34.10(26.39,41.92)	14.61(11.18,18.13)	-3.08(-3.22,-2.95)
Ghana	female	104.06(55.83,165.24)	151.55(84.44,250.55)	0.456302452	3.30(1.83,5.11)	1.79(1.03,2.86)	-1.86(-2.21,-1.52)
Greece	female	2201.20(1631.77,2791.92)	2295.66(1798.42,2803.63)	0.042910659	27.14(20.37,34.28)	21.27(16.72,26.01)	-1.14(-1.40,-0.88)
Greenland	female	12.89(9.11,17.12)	10.10(6.86,13.88)	-0.216475371	71.41(51.67,93.80)	30.64(21.17,41.56)	-3.47(-3.76,-3.18)
Grenada	female	4.00(2.55,5.72)	3.79(2.35,5.51)	-0.0523864	10.17(6.35,14.76)	6.46(4.00,9.35)	-1.25(-1.56,-0.93)
Guam	female	3.71(2.06,5.82)	6.31(3.57,9.48)	0.702532366	9.44(5.48,14.54)	6.56(3.69,9.90)	-1.52(-1.95,-1.09)
Guatemala	female	411.14(215.36,653.83)	829.97(421.22,1349.88)	1.01868162	22.29(12.17,35.04)	13.92(7.04,22.41)	-1.90(-2.30,-1.50)
Guinea	female	52.50(26.79,90.84)	79.57(41.06,130.70)	0.51540464	3.16(1.63,5.37)	3.01(1.57,4.90)	-0.17(-0.27,-0.07)
Guinea-Bissau	female	6.54(2.90,11.94)	7.57(3.65,12.90)	0.156973812	3.06(1.39,5.54)	1.93(0.96,3.25)	-1.21(-1.63,-0.79)
Guyana	female	19.96(12.04,30.74)	16.91(9.46,25.95)	-0.152718791	10.19(6.33,15.38)	5.00(2.86,7.56)	-2.53(-2.69,-2.36)
Haiti	female	225.46(103.39,381.40)	260.80(113.94,475.60)	0.156746695	12.87(6.27,21.38)	6.86(3.09,12.39)	-2.08(-2.27,-1.90)
Honduras	female	132.01(73.43,205.93)	338.04(189.98,555.74)	1.560697525	12.19(6.97,18.82)	10.42(5.86,16.92)	-0.36(-0.56,-0.15)
Hungary	female	3693.28(2802.32,4692.29)	2151.77(1558.42,2865.85)	-0.41738413	44.21(33.73,55.81)	21.13(15.21,28.03)	-2.58(-2.70,-2.46)

Iceland	female	52.36(40.11,65.76)	20.16(15.35,25.59)	-0.614980846	35.58(27.15,44.60)	7.29(5.52,9.25)	-5.66(-5.86,-5.47)
India	female	13343.21(7440.26,20842.76)	21483.66(13057.73,32512.42)	0.610080996	6.08(3.52,9.21)	3.72(2.27,5.57)	-2.00(-2.23,-1.76)
Indonesia	female	1429.28(716.28,2422.29)	2533.14(1304.99,4082.52)	0.772322057	2.85(1.45,4.64)	2.40(1.31,3.76)	-0.46(-0.55,-0.38)
Iran (Islamic Republic of)	female	1513.28(887.49,2304.85)	2283.78(1476.31,3170.77)	0.509163814	11.37(6.84,17.16)	6.22(4.07,8.61)	-1.99(-2.37,-1.60)
Iraq	female	187.23(111.86,291.45)	435.21(275.54,628.92)	1.324437455	4.75(2.86,7.33)	3.75(2.41,5.33)	-0.58(-0.80,-0.35)
Ireland	female	916.49(717.39,1110.80)	519.48(404.51,642.97)	-0.433179348	40.35(31.81,48.80)	13.31(10.32,16.56)	-4.13(-4.32,-3.94)
Israel	female	635.65(482.61,804.31)	567.53(425.98,720.45)	-0.107175832	24.73(18.75,31.40)	9.55(7.17,12.10)	-3.87(-4.08,-3.65)
Italy	female	17337.52(13415.15,21730.16)	10907.36(8439.27,13476.46)	-0.37088095	35.63(27.38,44.65)	15.67(12.01,19.42)	-3.33(-3.53,-3.13)
Jamaica	female	108.49(76.33,149.22)	102.26(67.62,147.53)	-0.05742255	11.59(8.09,16.16)	6.68(4.41,9.69)	-1.89(-2.17,-1.62)
Japan	female	42273.23(32128.12,53291.08)	19619.20(14636.41,24799.66)	-0.5358954	44.02(33.24,55.74)	12.09(8.97,15.30)	-4.40(-4.53,-4.26)
Jordan	female	61.91(38.31,94.48)	148.28(92.70,225.37)	1.395140406	9.13(5.89,13.54)	4.82(3.15,6.99)	-2.36(-2.54,-2.18)
Kazakhstan	female	1241.34(686.79,1912.73)	666.03(359.02,1015.78)	-0.463463733	15.96(8.83,24.89)	6.27(3.41,9.47)	-3.40(-3.62,-3.18)
Kenya	female	113.62(51.21,206.37)	295.13(142.53,547.51)	1.597509884	2.85(1.34,5.07)	2.73(1.35,4.88)	0.53(0.12,0.95)

Kiribati	female	6.59(3.86,10.11)	11.14(6.53,16.75)	0.689818133	31.66(19.17,47.56)	29.30(17.87,42.46)	-0.06(-0.16,0.04)
Kuwait	female	7.09(4.38,10.52)	10.63(6.70,15.99)	0.498302799	3.24(2.01,4.83)	1.07(0.69,1.56)	-3.39(-3.85,-2.93)
Kyrgyzstan	female	211.68(131.67,311.75)	166.59(105.74,238.87)	-0.213010612	11.90(7.53,17.46)	6.11(3.96,8.62)	-2.83(-3.57,-2.09)
Lao People's Democratic Republic	female	147.50(75.39,247.45)	101.27(52.80,168.88)	-0.313460802	13.25(6.96,21.84)	4.66(2.51,7.61)	-3.82(-4.15,-3.49)
Latvia	female	765.37(476.65,1078.21)	307.22(186.01,473.45)	-0.598602623	36.47(22.08,51.21)	15.46(9.01,24.06)	-3.44(-3.93,-2.95)
Lebanon	female	236.32(149.48,342.16)	491.57(331.64,678.03)	1.080097899	20.18(12.92,28.68)	17.23(11.61,23.85)	-0.50(-0.67,-0.32)
Lesotho	female	20.00(12.07,30.93)	27.39(15.38,43.75)	0.369765881	3.86(2.34,5.93)	4.25(2.42,6.79)	1.18(0.84,1.52)
Liberia	female	22.25(10.19,38.36)	24.80(11.10,45.51)	0.114790283	4.23(1.99,7.23)	2.64(1.23,4.74)	-1.09(-1.44,-0.74)
Libya	female	8.61(4.54,15.14)	13.28(7.15,21.98)	0.541652352	0.98(0.53,1.72)	0.53(0.29,0.84)	-2.26(-2.57,-1.96)
Lithuania	female	665.10(409.77,943.66)	343.33(218.37,488.01)	-0.483783793	25.77(15.74,36.78)	12.62(7.79,18.38)	-2.76(-3.23,-2.28)
Luxembourg	female	86.97(63.55,112.32)	46.50(34.08,60.49)	-0.46535124	28.45(20.79,36.35)	8.93(6.47,11.69)	-3.99(-4.06,-3.92)
North Macedonia	female	562.87(387.92,752.06)	557.05(379.29,765.39)	-0.010339501	54.80(38.23,72.69)	32.64(22.05,44.68)	-2.10(-2.23,-1.96)
Madagascar	female	68.61(29.77,120.55)	73.96(29.81,133.44)	0.077977983	2.55(1.14,4.47)	1.25(0.54,2.25)	-2.87(-3.15,-2.59)

Malawi	female	29.10(12.44,52.46)	36.03(15.50,62.76)	0.238172687	1.46(0.64,2.60)	0.94(0.41,1.63)	-1.46(-1.60,-1.32)
Malaysia	female	155.17(86.80,248.26)	237.42(136.81,373.04)	0.530140165	3.50(1.97,5.46)	1.93(1.14,3.00)	-2.10(-2.31,-1.89)
Maldives	female	2.86(1.17,5.06)	2.43(1.41,3.66)	-0.151389746	7.90(3.41,13.35)	1.98(1.15,2.98)	-5.63(-5.96,-5.30)
Mali	female	69.53(27.53,129.49)	153.59(62.40,278.93)	1.209049101	3.19(1.29,5.89)	3.66(1.48,6.59)	0.39(0.33,0.45)
Malta	female	50.24(37.42,64.26)	31.35(23.68,40.50)	-0.37600917	21.17(15.73,27.14)	7.22(5.45,9.31)	-4.30(-4.57,-4.04)
Marshall Islands	female	1.15(0.64,1.85)	2.05(1.01,3.46)	0.776243805	13.30(7.50,21.03)	11.36(5.97,18.45)	-0.70(-0.80,-0.61)
Mauritania	female	28.38(12.71,49.18)	37.86(16.23,70.18)	0.334273577	5.43(2.47,9.39)	3.44(1.52,6.32)	-1.31(-1.44,-1.17)
Mauritius	female	23.18(12.97,35.76)	20.16(11.21,31.67)	-0.130207191	5.78(3.28,8.82)	2.10(1.17,3.26)	-3.86(-4.15,-3.58)
Mexico	female	3953.80(2407.44,5850.78)	3390.40(1777.93,5334.06)	-0.142495296	18.39(11.57,26.63)	5.43(2.88,8.50)	-4.12(-4.21,-4.03)
Republic of Moldova	female	261.50(156.23,383.79)	149.60(94.10,217.60)	-0.427910269	9.69(5.80,14.24)	4.61(2.87,6.76)	-1.91(-2.36,-1.46)
Mongolia	female	177.64(87.02,293.23)	242.87(122.66,407.17)	0.367183654	31.33(15.43,51.47)	19.49(9.83,32.12)	-2.35(-2.64,-2.07)
Montenegro	female	79.72(57.64,107.21)	112.69(82.04,146.27)	0.413546983	22.78(16.38,30.53)	21.07(15.27,27.44)	-0.31(-0.65,0.03)
Morocco	female	80.15(47.93,123.99)	115.82(67.17,184.43)	0.445010947	1.12(0.68,1.70)	0.71(0.43,1.11)	-1.56(-1.68,-1.44)

Mozambique	female	76.93(32.54,134.84)	123.28(49.83,226.11)	0.602438353	2.56(1.10,4.43)	2.16(0.89,3.92)	-0.22(-0.48,0.04)
Myanmar	female	3393.64(1920.08,5277.74)	1895.43(1044.75,2963.77)	-0.441476562	28.05(16.30,42.69)	7.76(4.45,11.98)	-4.49(-4.80,-4.17)
Namibia	female	21.97(12.36,33.27)	26.81(15.13,42.29)	0.220370417	5.87(3.44,8.72)	3.61(2.06,5.69)	-1.60(-1.78,-1.41)
Nepal	female	936.94(439.20,1582.15)	1601.76(777.02,2654.13)	0.709560576	20.70(10.35,33.26)	14.26(7.09,23.47)	-0.81(-1.05,-0.57)
Netherlands	female	4795.33(3784.95,5764.98)	3092.41(2388.08,3836.41)	-0.355121269	42.53(33.78,51.00)	17.25(13.37,21.45)	-3.26(-3.43,-3.08)
New Zealand	female	571.87(443.52,701.02)	356.81(264.75,461.23)	-0.376065041	27.36(21.20,33.42)	9.25(6.79,12.05)	-3.65(-3.70,-3.60)
Nicaragua	female	75.41(41.54,117.92)	119.75(65.25,192.32)	0.588000624	8.63(4.80,13.57)	4.70(2.64,7.49)	-2.65(-2.98,-2.32)
Niger	female	15.87(6.65,28.72)	52.12(20.95,96.47)	2.284128317	1.16(0.49,2.05)	1.27(0.53,2.30)	0.51(0.40,0.62)
Nigeria	female	205.10(102.44,349.23)	234.74(112.15,419.46)	0.144504372	1.02(0.51,1.74)	0.54(0.28,0.93)	-2.05(-2.21,-1.88)
Democratic People's Republic of Korea	female	961.91(513.39,1618.20)	1642.38(875.13,2699.81)	0.707418662	9.61(5.15,15.89)	8.84(4.66,14.44)	-0.43(-0.73,-0.13)
Northern Mariana Islands	female	2.11(1.04,3.46)	3.18(1.63,5.12)	0.5086756	27.62(14.98,42.98)	11.94(6.51,18.87)	-3.00(-3.36,-2.63)
Norway	female	887.12(671.15,1134.01)	340.84(255.73,440.83)	-0.615788866	26.33(19.82,33.73)	7.37(5.40,9.63)	-4.34(-4.72,-3.96)
Oman	female	12.22(6.86,20.19)	10.03(5.96,15.43)	-0.178670728	4.29(2.46,7.04)	1.55(0.94,2.28)	-3.59(-3.74,-3.44)

Pakistan	female	1065.76(591.28,1615.61)	1550.05(866.04,2433.16)	0.454414017	4.14(2.34,6.28)	2.90(1.71,4.41)	-1.03(-1.41,-0.64)
Palestine	female	20.23(10.96,34.89)	29.99(17.99,44.39)	0.48236469	4.29(2.38,7.27)	2.50(1.53,3.60)	-1.80(-1.90,-1.69)
Panama	female	90.06(60.34,127.22)	103.52(64.06,152.68)	0.149476336	12.27(8.22,17.27)	4.84(2.97,7.17)	-3.10(-3.25,-2.96)
Papua New Guinea	female	165.20(76.26,286.10)	345.41(159.95,611.59)	1.090856461	17.73(8.23,29.82)	15.39(7.64,26.60)	-0.10(-0.31,0.12)
Paraguay	female	211.58(138.41,302.93)	262.34(156.97,388.49)	0.239914216	18.65(12.27,26.65)	9.19(5.55,13.56)	-2.88(-3.50,-2.25)
Peru	female	505.64(238.39,841.60)	796.25(333.60,1378.25)	0.57474806	8.18(3.89,13.55)	4.80(2.01,8.35)	-1.87(-2.20,-1.55)
Philippines	female	1089.09(632.32,1672.24)	1598.63(956.16,2393.55)	0.467862409	7.84(4.77,11.61)	4.10(2.53,6.06)	-2.12(-2.29,-1.95)
Poland	female	9465.78(7189.08,11871.19)	7386.58(5337.99,9802.79)	-0.219653798	37.65(28.52,46.96)	19.58(13.95,26.04)	-2.50(-2.64,-2.36)
Portugal	female	2080.15(1481.89,2761.11)	1245.32(876.96,1638.81)	-0.401331564	27.82(19.71,37.29)	12.24(8.34,16.57)	-3.18(-3.46,-2.89)
Puerto Rico	female	177.52(117.42,246.08)	157.92(100.28,229.08)	-0.110411774	9.02(5.99,12.61)	4.15(2.59,6.15)	-3.19(-3.37,-3.01)
Qatar	female	1.31(0.70,2.18)	3.53(1.88,5.79)	1.69500404	3.18(1.78,5.32)	1.59(0.96,2.52)	-2.11(-2.45,-1.77)
Romania	female	3568.35(2371.92,4900.96)	2281.62(1544.49,3159.97)	-0.360593656	22.61(14.98,31.18)	12.38(8.11,17.20)	-2.26(-2.37,-2.15)
Russian Federation	female	21679.06(12918.76,32736.46)	17704.30(11093.82,25133.72)	-0.183345372	19.61(11.33,29.38)	13.73(8.46,19.78)	-1.49(-1.93,-1.05)

Rwanda	female	140.33(66.10,240.05)	236.08(129.40,367.07)	0.682348318	9.25(4.45,15.39)	7.60(4.32,11.61)	-1.36(-1.64,-1.08)
Saint Lucia	female	6.66(4.46,9.28)	6.63(4.36,9.41)	-0.004225149	13.82(9.25,19.18)	5.82(3.83,8.26)	-3.42(-3.77,-3.06)
Saint Vincent and the Grenadines	female	3.20(2.04,4.52)	3.19(1.92,4.75)	-0.002347872	8.12(5.17,11.66)	4.84(2.94,7.15)	-1.83(-2.18,-1.48)
Samoa	female	10.08(6.11,15.99)	14.32(8.67,21.42)	0.420018841	22.28(13.63,34.92)	19.22(11.70,28.21)	-0.46(-0.56,-0.37)
Sao Tome and Principe	female	1.08(0.48,1.90)	1.90(0.86,3.23)	0.762069248	3.15(1.42,5.50)	3.86(1.75,6.58)	0.90(0.78,1.02)
Saudi Arabia	female	39.05(23.32,60.85)	68.68(41.78,107.21)	0.758830909	1.64(0.97,2.53)	1.02(0.65,1.56)	-1.63(-1.76,-1.51)
Senegal	female	32.42(13.94,59.17)	55.36(25.30,99.75)	0.707674277	2.02(0.89,3.69)	1.42(0.66,2.58)	-0.65(-0.95,-0.34)
Serbia	female	1991.19(1311.46,2778.59)	1699.60(1161.44,2361.54)	-0.146438284	31.53(20.86,43.36)	21.33(14.32,30.17)	-1.90(-2.15,-1.65)
Seychelles	female	1.30(0.77,2.02)	1.42(0.83,2.11)	0.089327447	4.20(2.44,6.50)	2.47(1.47,3.66)	-1.95(-2.12,-1.78)
Sierra Leone	female	77.75(30.41,137.38)	92.99(36.49,164.86)	0.195993306	8.20(3.26,14.38)	5.15(2.02,9.12)	-0.91(-1.22,-0.60)
Singapore	female	219.98(152.84,300.98)	144.54(103.19,194.91)	-0.342933477	17.72(12.43,24.04)	3.56(2.53,4.80)	-5.61(-5.83,-5.40)
Slovakia	female	955.49(656.05,1286.30)	565.41(356.83,805.06)	-0.408248742	29.04(20.08,39.05)	11.62(7.15,16.73)	-3.05(-3.27,-2.83)
Slovenia	female	644.02(431.39,903.92)	349.86(244.53,477.76)	-0.456751162	44.85(30.08,63.21)	16.63(11.45,23.02)	-3.88(-4.15,-3.62)

Solomon Islands	female	23.09(10.77,40.71)	46.39(22.77,77.09)	1.009469355	34.32(17.59,59.26)	28.52(15.02,46.19)	-0.77(-0.90,-0.64)
Somalia	female	36.46(14.20,70.42)	82.95(30.60,169.54)	1.275127708	2.81(1.11,5.37)	2.25(0.85,4.57)	-0.61(-0.67,-0.56)
South Africa	female	1618.87(1068.94,2255.52)	1244.26(842.36,1697.87)	-0.23140048	13.37(8.89,18.58)	4.83(3.29,6.52)	-3.61(-4.02,-3.20)
Republic of Korea	female	7548.10(5137.67,10301.03)	3407.88(2453.66,4507.39)	-0.54851223	42.35(29.19,57.35)	7.14(5.22,9.40)	-6.82(-7.08,-6.55)
South Sudan	female	24.38(9.16,48.15)	27.26(9.71,56.01)	0.118063814	2.40(0.91,4.73)	1.62(0.59,3.22)	-1.38(-1.42,-1.34)
Spain	female	6510.61(4732.58,8286.94)	5947.82(4537.98,7443.00)	-0.08644222	23.82(17.28,29.94)	14.35(10.82,18.05)	-1.58(-2.10,-1.05)
Sri Lanka	female	183.48(103.79,287.99)	164.29(92.04,261.11)	-0.104615205	3.59(2.08,5.48)	1.16(0.66,1.85)	-4.09(-4.30,-3.88)
Sudan	female	238.98(126.81,391.63)	386.61(209.34,624.51)	0.617700296	5.21(2.76,8.38)	4.41(2.38,6.89)	-0.15(-0.30,0.00)
Suriname	female	13.53(8.75,19.72)	21.38(13.41,31.65)	0.580235861	9.97(6.49,14.62)	6.52(4.07,9.52)	-1.72(-2.01,-1.44)
Eswatini	female	7.25(4.01,11.37)	8.50(4.36,13.94)	0.172093235	5.11(2.84,7.98)	2.92(1.54,4.73)	-1.51(-1.94,-1.08)
Sweden	female	2029.54(1577.72,2489.58)	980.66(755.20,1211.19)	-0.516808287	26.34(20.12,32.29)	9.31(7.21,11.44)	-3.50(-3.64,-3.36)
Switzerland	female	1165.40(888.41,1444.22)	710.70(547.89,890.14)	-0.390166562	21.13(16.25,26.31)	8.20(6.36,10.28)	-3.21(-3.43,-2.98)
Syrian Arab Republic	female	114.27(65.94,174.54)	161.40(93.78,257.44)	0.412387319	4.40(2.67,6.69)	2.64(1.61,4.09)	-1.94(-2.20,-1.69)

Taiwan (Province of China)	female	576.66(335.81,833.52)	528.13(312.69,819.13)	-0.08416554	7.11(4.17,10.21)	2.65(1.58,4.10)	-3.58(-3.72,-3.44)
Tajikistan	female	162.76(94.86,249.02)	177.91(102.55,268.27)	0.093094194	10.50(6.14,16.02)	6.16(3.70,9.16)	-1.58(-1.85,-1.30)
United Republic of Tanzania	female	475.68(208.53,853.00)	852.21(384.25,1496.03)	0.791554775	8.48(3.78,15.02)	6.75(3.15,11.75)	-0.61(-0.70,-0.52)
Thailand	female	1482.67(863.59,2248.76)	1040.59(602.65,1614.41)	-0.298165745	8.01(4.83,12.01)	1.87(1.08,2.89)	-5.40(-5.69,-5.11)
Bahamas	female	7.62(4.82,10.65)	10.86(6.89,16.29)	0.425734992	8.86(5.61,12.38)	5.06(3.22,7.54)	-1.90(-2.05,-1.75)
Gambia	female	1.36(0.60,2.45)	2.62(1.21,4.62)	0.921568704	0.90(0.41,1.59)	0.57(0.27,0.99)	-1.60(-1.78,-1.42)
Timor-Leste	female	7.08(2.85,12.75)	11.16(4.75,20.49)	0.576829689	5.32(2.28,9.42)	2.77(1.24,5.02)	-2.45(-2.65,-2.24)
Togo	female	43.79(24.01,68.99)	92.51(53.20,146.91)	1.112711695	7.23(3.98,11.27)	4.76(2.74,7.45)	-1.26(-1.38,-1.15)
Tonga	female	4.07(2.28,6.26)	3.94(2.26,6.12)	-0.030516724	14.37(8.30,21.86)	9.40(5.37,14.75)	-1.51(-1.62,-1.40)
Trinidad and Tobago	female	47.23(32.67,64.00)	28.77(17.55,43.89)	-0.390818131	10.64(7.38,14.39)	2.96(1.81,4.53)	-5.19(-5.61,-4.76)
Tunisia	female	59.59(36.48,89.99)	94.75(56.76,150.61)	0.59002911	2.45(1.50,3.65)	1.46(0.88,2.28)	-1.80(-1.89,-1.71)
Turkey	female	5236.28(3163.80,7857.50)	5240.49(3391.03,7421.34)	0.000805262	25.73(15.74,37.79)	11.01(7.14,15.62)	-2.77(-3.40,-2.15)
Turkmenistan	female	137.75(83.23,199.57)	87.75(50.30,138.16)	-0.362927917	11.87(7.30,17.29)	3.59(2.11,5.55)	-4.49(-4.99,-3.99)

Uganda	female	79.75(32.87,146.83)	178.62(79.64,303.66)	1.23974337	2.59(1.09,4.71)	2.50(1.15,4.24)	-0.19(-0.49,0.10)
Ukraine	female	9066.18(5253.91,13785.50)	4237.95(2388.22,6557.23)	-0.532553744	22.31(12.84,34.24)	10.85(5.91,17.04)	-3.40(-3.90,-2.90)
United Arab Emirates	female	11.66(6.58,19.87)	44.06(23.39,72.05)	2.78001455	8.03(4.69,13.29)	3.56(2.18,5.63)	-2.28(-3.12,-1.43)
United Kingdom	female	19607.48(15886.57,23370.12)	9025.22(7145.84,10904.73)	-0.539705341	37.77(30.70,45.13)	13.88(11.03,16.73)	-3.74(-3.90,-3.57)
United States of America	female	31639.72(25571.57,37430.61)	23512.74(18820.56,28234.99)	-0.256860234	17.89(14.60,21.04)	8.23(6.58,9.83)	-2.78(-2.85,-2.71)
Uruguay	female	583.42(405.26,767.08)	473.96(333.47,625.31)	-0.187619458	28.00(19.11,37.07)	17.39(11.97,23.16)	-1.23(-1.40,-1.06)
Uzbekistan	female	271.39(139.11,432.80)	383.19(185.52,645.83)	0.411966602	4.09(2.14,6.51)	2.71(1.37,4.47)	-1.46(-1.67,-1.25)
Vanuatu	female	1.87(0.89,3.22)	3.46(1.66,6.25)	0.849099961	6.67(3.31,11.24)	4.34(2.21,7.65)	-1.46(-1.63,-1.30)
Venezuela (Bolivarian Republic of)	female	1632.03(1032.02,2346.91)	1704.18(954.52,2762.03)	0.044208451	32.21(20.44,45.96)	11.08(6.25,17.75)	-4.09(-4.44,-3.73)
Viet nam	female	1049.42(517.42,1788.85)	1029.69(541.16,1642.83)	-0.01880521	4.51(2.22,7.65)	2.01(1.06,3.20)	-3.14(-3.66,-2.62)
Virginia	female	660.82(516.19,807.95)	579.19(422.58,762.64)	-0.123525962	16.50(12.94,20.11)	7.79(5.67,10.38)	-2.77(-2.86,-2.67)
Yemen	female	524.72(281.60,847.49)	1324.94(799.80,2008.34)	1.525067872	19.91(10.87,32.12)	18.59(11.64,27.52)	-0.33(-0.42,-0.24)
Zambia	female	58.85(26.16,100.02)	111.01(50.34,191.62)	0.886401641	5.25(2.45,8.79)	3.70(1.76,6.17)	-1.01(-1.22,-0.79)

Zimbabwe	female	201.77(122.04,314.73)	317.14(178.28,500.11)	0.571800624	10.61(6.37,16.08)	8.59(5.04,13.20)	0.89(0.22,1.56)
Monaco	female	9.05(5.78,12.88)	5.36(3.45,7.72)	-0.408277172	25.37(16.61,35.89)	11.75(7.73,16.95)	-2.63(-2.91,-2.35)
San Marino	female	9.91(6.90,13.38)	8.56(4.91,14.16)	-0.136699674	55.89(39.26,74.88)	27.64(15.89,46.95)	-2.20(-2.35,-2.05)
Saint Kitts and Nevis	female	1.62(1.10,2.30)	1.19(0.77,1.75)	-0.264234804	7.65(5.21,10.79)	3.59(2.38,5.23)	-2.03(-2.24,-1.82)
Cook Islands	female	0.71(0.38,1.12)	0.68(0.39,1.05)	-0.053712271	11.78(6.53,18.26)	5.44(3.07,8.51)	-2.64(-2.91,-2.38)
Nauru	female	0.83(0.40,1.42)	0.90(0.45,1.50)	0.081869452	40.80(21.87,68.36)	32.74(17.07,53.43)	-0.52(-0.84,-0.20)
Niue	female	0.17(0.09,0.26)	0.11(0.06,0.16)	-0.350490137	14.06(7.71,22.51)	9.25(5.41,14.30)	-1.60(-1.70,-1.51)
Palau	female	0.32(0.16,0.53)	0.41(0.22,0.65)	0.298383052	6.17(3.21,10.13)	3.74(2.08,5.73)	-1.50(-1.63,-1.37)
Tokelau	female	0.16(0.09,0.25)	0.09(0.05,0.14)	-0.452824427	21.42(11.59,33.92)	13.06(7.30,21.03)	-1.68(-1.72,-1.64)
Tuvalu	female	1.09(0.59,1.80)	0.95(0.50,1.53)	-0.126649119	26.40(14.63,43.26)	16.90(9.05,26.90)	-1.52(-1.56,-1.47)
Afghanistan	male	2531.13(1518.08,3666.49)	6164.12(3814.85,8920.88)	1.435318273	66.23(40.46,95.12)	97.87(63.40,136.51)	1.51(1.35,1.68)
Albania	male	1567.24(1267.39,1895.85)	2189.24(1491.35,3045.98)	0.396874435	151.99(123.26,182.78)	106.78(72.91,147.59)	-1.26(-1.51,-1.00)
Algeria	male	3566.30(2534.79,4694.59)	4855.97(3456.31,6609.59)	0.361625599	61.87(45.09,81.12)	29.10(21.11,39.15)	-2.71(-2.83,-2.58)

American Samoa	male	15.05(10.04,20.72)	19.56(13.13,27.37)	0.299671171	121.38(84.04,160.97)	80.85(55.14,110.29)	-1.47(-1.63,-1.32)
Andorra	male	24.84(17.05,35.15)	30.65(21.64,42.17)	0.233636704	85.99(60.25,120.59)	43.52(30.77,59.82)	-2.39(-2.44,-2.34)
Angola	male	1303.64(770.48,1900.85)	1937.90(1240.36,2804.66)	0.48653324	62.87(38.92,89.47)	36.58(24.47,51.07)	-2.26(-2.42,-2.10)
Antigua and Barbuda	male	14.51(10.81,18.74)	17.52(12.27,23.32)	0.207381188	63.90(47.51,81.77)	36.33(25.87,48.08)	-1.96(-2.24,-1.69)
Argentina	male	18647.75(14917.52,22287.91)	15086.65(11869.13,18297.39)	-0.190966913	125.63(100.36,149.56)	62.98(49.45,76.33)	-2.34(-2.42,-2.27)
Armenia	male	3963.44(3264.75,4619.63)	2618.18(2018.34,3226.14)	-0.339417982	308.09(255.29,355.75)	145.07(111.08,178.02)	-2.86(-3.06,-2.66)
Australia	male	4721.02(3819.03,5593.30)	2873.89(2226.77,3525.06)	-0.39125796	52.18(42.29,61.94)	15.59(11.98,19.14)	-4.21(-4.39,-4.04)
Austria	male	6072.86(4963.42,7177.37)	2654.98(2135.53,3185.37)	-0.562811722	132.91(109.24,157.13)	34.59(27.78,41.48)	-4.99(-5.25,-4.73)
Azerbaijan	male	8121.36(6275.11,9923.06)	9587.50(6938.30,12903.11)	0.18052862	353.21(277.18,428.68)	214.74(157.78,285.48)	-1.76(-2.06,-1.46)
Bahrain	male	73.43(54.11,94.31)	126.19(86.53,176.60)	0.718413606	84.61(63.51,106.89)	25.29(17.91,33.77)	-4.60(-4.94,-4.26)
Bangladesh	male	22964.72(15727.25,30699.26)	23240.90(15315.12,34049.80)	0.012026574	88.53(61.66,116.53)	34.16(22.87,49.95)	-3.25(-3.45,-3.04)
Barbados	male	80.68(60.99,102.53)	64.97(46.39,86.78)	-0.194684668	67.16(50.48,85.10)	29.55(21.03,39.62)	-3.29(-3.50,-3.08)
Belarus	male	20324.11(16193.43,24247.67)	9821.84(7099.62,13203.63)	-0.516739313	399.66(321.89,475.67)	157.24(115.56,209.58)	-3.92(-4.19,-3.65)

Belgium	male	5814.17(4759.74,6822.20)	3205.60(2585.77,3825.78)	-0.448657501	90.53(74.40,106.15)	32.32(26.17,38.48)	-3.43(-3.53,-3.33)
Belize	male	25.85(18.80,33.30)	58.27(40.86,77.68)	1.254149042	56.19(40.93,72.28)	41.03(29.13,54.06)	-1.66(-2.25,-1.06)
Benin	male	529.06(334.66,755.88)	632.05(378.33,957.73)	0.194668534	55.59(35.31,78.76)	29.40(18.37,43.72)	-1.77(-1.95,-1.59)
Bermuda	male	17.10(11.89,22.66)	13.42(9.44,18.05)	-0.215615293	62.14(44.06,81.39)	23.20(16.29,31.31)	-3.10(-3.34,-2.87)
Bhutan	male	37.88(21.78,57.12)	52.95(32.04,79.34)	0.398025783	31.11(18.66,45.76)	18.85(11.46,27.92)	-1.58(-1.72,-1.45)
Bolivia (Plurinational State of)	male	3112.16(2051.74,4365.99)	3644.22(2269.79,5320.16)	0.170961159	209.54(139.95,290.58)	87.29(55.84,125.95)	-3.27(-3.64,-2.90)
Bosnia and Herzegovina	male	2403.96(1894.50,2901.46)	2714.13(1972.03,3656.41)	0.129026274	126.24(101.17,152.63)	100.90(74.14,135.55)	-1.02(-1.28,-0.76)
Botswana	male	173.35(101.31,261.48)	281.05(175.00,413.32)	0.621304242	66.92(40.70,98.54)	46.49(30.88,65.44)	-2.17(-2.89,-1.44)
Brazil	male	72515.42(58752.60,86417.49)	57755.00(44595.90,71015.13)	-0.203548759	170.22(138.73,202.01)	53.34(41.40,65.02)	-4.01(-4.22,-3.79)
Brunei Darussalam	male	134.76(105.01,169.24)	105.55(77.65,141.03)	-0.216723759	288.00(224.65,360.98)	77.63(58.03,100.55)	-4.62(-4.95,-4.30)
Bulgaria	male	11984.41(9759.59,14147.95)	6686.42(4873.92,8704.81)	-0.442073508	197.82(161.65,232.52)	110.66(80.55,144.46)	-1.10(-1.50,-0.69)
Burkina Faso	male	890.82(457.04,1405.79)	1169.04(606.19,1859.86)	0.31231814	40.68(21.42,63.54)	27.16(14.80,42.28)	-1.21(-1.50,-0.92)
Burundi	male	707.72(395.38,1073.70)	575.66(292.60,927.56)	-0.186607711	65.40(37.15,98.34)	23.07(12.43,36.11)	-4.35(-4.64,-4.07)

Cambodia	male	3043.95(2151.71,4185.84)	3947.26(2827.02,5210.90)	0.296756037	156.63(112.22,212.98)	81.94(60.05,104.60)	-2.13(-2.21,-2.06)
Cameroon	male	1191.34(745.88,1737.96)	2066.05(1185.73,3233.38)	0.734227756	52.11(33.80,74.78)	34.94(20.73,54.00)	-1.22(-1.41,-1.03)
Canada	male	9850.78(8117.48,11556.83)	7380.23(5881.33,8952.64)	-0.250797835	68.00(56.37,79.63)	23.37(18.54,28.28)	-3.68(-3.80,-3.55)
Cabo Verde	male	88.26(57.88,119.45)	119.31(79.79,165.05)	0.351784542	93.55(60.91,127.63)	66.06(44.87,91.60)	-2.58(-3.09,-2.08)
Central African Republic	male	380.23(219.89,575.56)	432.60(237.17,677.60)	0.137721814	63.11(38.01,92.88)	38.20(22.85,56.80)	-2.00(-2.17,-1.83)
Chad	male	636.41(368.38,956.76)	1063.36(602.41,1623.09)	0.670877998	46.62(27.37,69.58)	35.49(20.83,54.04)	-0.72(-0.89,-0.54)
Chile	male	10250.59(7673.74,12844.57)	9952.59(7339.99,12590.71)	-0.029071834	217.06(163.39,271.00)	89.66(66.54,113.11)	-2.97(-3.10,-2.84)
China	male	1422885.60(1051944.92,1841573.05)	2034269.90(1500766.82,2611029.35)	0.429679165	331.73(248.99,423.69)	202.59(150.56,257.01)	-1.61(-2.08,-1.14)
Colombia	male	12908.10(9451.71,16453.75)	9172.79(5513.79,13688.27)	-0.289377371	146.96(109.35,185.92)	38.14(23.11,56.60)	-4.67(-4.84,-4.50)
Comoros	male	42.71(19.32,67.69)	51.97(29.85,79.00)	0.216935512	39.99(18.99,62.41)	23.66(13.87,35.47)	-2.00(-2.18,-1.83)
Congo	male	256.83(156.08,374.03)	323.44(192.92,496.37)	0.259383797	52.11(32.43,74.42)	24.79(15.87,35.93)	-3.12(-3.44,-2.79)
Costa Rica	male	2062.32(1597.58,2529.99)	1863.18(1242.90,2646.49)	-0.096557196	246.00(191.33,300.78)	78.42(52.64,111.49)	-4.28(-4.62,-3.95)
C 么 te d'Ivoire	male	1320.75(839.42,1891.72)	2504.65(1572.28,3614.63)	0.8963923	55.93(36.48,77.65)	42.54(28.18,59.86)	-0.94(-1.08,-0.81)

Croatia	male	6929.35(5623.50,8157.91)	2933.20(2141.03,3892.21)	-0.576699817	253.66(207.17,298.30)	79.11(57.67,104.39)	-4.13(-4.31,-3.96)
Cuba	male	2938.58(2360.46,3486.67)	3629.77(2647.91,4719.99)	0.235211259	58.02(46.46,68.86)	40.70(29.64,53.07)	-0.99(-1.09,-0.89)
Cyprus	male	228.25(173.65,288.11)	421.50(326.66,526.11)	0.84664271	58.99(45.15,74.41)	45.12(34.87,56.36)	-0.75(-0.99,-0.51)
Czechia	male	9021.24(7257.25,10642.54)	4061.64(2956.43,5258.36)	-0.549769889	157.00(126.06,185.42)	43.83(31.96,56.53)	-4.37(-4.47,-4.28)
Democratic Republic of the Congo	male	2970.53(1452.21,4754.09)	3446.45(1718.46,5826.70)	0.160213699	38.01(19.22,60.09)	20.02(10.42,32.82)	-2.38(-2.47,-2.29)
Denmark	male	2974.18(2454.74,3478.39)	1470.83(1170.84,1766.72)	-0.505466811	86.32(71.63,100.87)	27.92(22.35,33.35)	-3.62(-3.82,-3.42)
Djibouti	male	34.31(17.65,55.03)	131.97(69.19,210.03)	2.846083081	45.78(25.26,69.88)	38.81(22.71,58.82)	-0.98(-1.14,-0.81)
Dominica	male	34.73(25.27,45.04)	26.82(18.08,37.39)	-0.22794298	119.62(85.38,155.35)	59.95(40.18,83.51)	-2.53(-2.83,-2.23)
Dominican Republic	male	598.18(437.37,776.02)	1845.90(1203.87,2705.78)	2.085876401	32.74(24.21,42.20)	41.42(27.38,60.27)	1.35(1.14,1.57)
Ecuador	male	3319.13(2296.12,4464.63)	4428.40(2780.77,6540.71)	0.33420332	129.27(91.11,171.93)	61.97(39.26,90.76)	-2.63(-2.80,-2.47)
Egypt	male	5442.20(4181.97,6780.98)	12198.15(8277.93,17708.27)	1.241400449	36.84(28.55,45.36)	34.49(23.79,49.00)	-0.18(-0.28,-0.08)
El Salvador	male	718.76(512.99,963.69)	1074.45(660.61,1623.30)	0.494863064	51.27(36.85,67.96)	43.57(26.76,65.97)	-1.04(-1.35,-0.73)
Equatorial Guinea	male	52.92(26.13,86.27)	39.61(20.06,67.18)	-0.251647401	55.47(28.68,89.58)	19.58(10.58,32.16)	-4.25(-4.82,-3.68)

Eritrea	male	237.90(120.92,388.71)	376.12(209.49,604.51)	0.581027049	45.49(24.28,72.07)	26.46(15.37,39.97)	-2.09(-2.38,-1.81)
Estonia	male	2207.00(1755.68,2646.77)	969.49(684.90,1312.78)	-0.560719023	279.62(222.90,333.93)	100.07(70.71,135.27)	-3.67(-3.88,-3.45)
Ethiopia	male	2776.84(1342.12,4306.60)	1399.67(732.19,2247.54)	-0.495948859	24.87(12.56,38.56)	6.72(3.58,10.63)	-5.08(-5.40,-4.76)
Micronesia (Federated States of)	male	35.00(21.44,51.56)	41.07(21.67,63.47)	0.173385682	138.04(85.61,197.85)	107.52(62.66,156.56)	-0.92(-1.03,-0.80)
Fiji	male	101.47(64.99,143.07)	120.36(68.71,181.77)	0.186176395	53.69(36.15,73.12)	32.77(20.12,47.54)	-1.59(-1.78,-1.40)
Finland	male	2637.04(2100.58,3156.76)	1208.10(942.23,1489.40)	-0.541873269	91.83(73.54,109.91)	23.11(17.96,28.39)	-4.73(-4.96,-4.51)
France	male	28031.53(22638.95,32997.69)	16913.26(13429.19,20704.37)	-0.39663424	81.99(66.34,96.56)	30.08(23.71,36.99)	-3.49(-3.60,-3.39)
Gabon	male	105.11(60.75,160.92)	126.60(71.93,195.73)	0.20451025	39.83(23.63,59.63)	24.22(14.68,36.23)	-1.73(-1.80,-1.67)
Georgia	male	5559.42(4336.22,6882.15)	3432.55(2587.00,4371.54)	-0.382570085	204.45(160.29,250.39)	141.86(106.62,180.32)	-0.39(-0.90,0.12)
Germany	male	52828.06(43543.19,62161.08)	34368.45(27601.68,41224.11)	-0.349428095	107.69(89.06,126.55)	42.55(33.92,51.05)	-3.20(-3.36,-3.04)
Ghana	male	618.40(341.37,975.92)	620.11(330.67,966.75)	0.002750128	21.62(12.79,32.81)	9.36(5.36,14.25)	-3.29(-3.73,-2.85)
Greece	male	9008.94(7444.17,10487.66)	7015.84(5743.63,8252.03)	-0.221235404	128.12(106.38,148.60)	71.20(58.54,83.98)	-2.40(-2.54,-2.26)
Greenland	male	34.86(24.94,44.79)	33.96(23.45,45.75)	-0.026005465	184.51(139.58,230.10)	84.73(60.02,112.34)	-2.72(-2.85,-2.59)

Grenada	male	17.87(12.88,23.64)	17.80(12.50,23.99)	-0.004021914	60.79(43.30,80.72)	31.71(22.66,41.94)	-1.93(-2.23,-1.62)
Guam	male	15.49(10.24,21.50)	24.54(15.72,35.61)	0.584754913	37.13(25.17,50.45)	25.51(16.45,36.83)	-1.21(-1.51,-0.91)
Guatemala	male	1432.64(877.02,2057.24)	2829.84(1616.35,4380.24)	0.975256615	79.93(50.99,111.75)	57.01(33.37,86.30)	-1.38(-1.95,-0.81)
Guinea	male	834.86(493.12,1217.68)	1725.61(1025.70,2556.52)	1.06694004	49.15(29.57,71.20)	60.68(36.79,89.37)	0.98(0.72,1.24)
Guinea-Bissau	male	128.58(74.50,192.56)	108.50(64.90,162.64)	-0.156142001	62.35(36.59,92.43)	31.94(19.56,47.24)	-2.01(-2.46,-1.57)
Guyana	male	114.51(81.35,154.93)	99.61(62.06,146.11)	-0.13007712	60.54(43.33,80.83)	30.94(20.01,44.82)	-2.27(-2.41,-2.12)
Haiti	male	1148.73(619.54,1782.09)	1099.75(558.55,1816.76)	-0.042637771	67.37(37.22,103.18)	32.25(16.99,52.12)	-2.53(-2.59,-2.46)
Honduras	male	660.46(454.69,909.03)	1570.41(1058.67,2203.10)	1.377754482	63.97(44.50,87.81)	55.67(38.01,77.18)	-0.21(-0.51,0.08)
Hungary	male	11968.47(9649.54,13988.11)	5634.19(4229.46,7232.27)	-0.52924746	191.17(154.43,223.79)	72.04(54.32,92.29)	-3.43(-3.55,-3.31)
Iceland	male	137.44(112.55,163.67)	70.43(55.00,87.25)	-0.487573935	105.31(86.23,125.61)	27.25(21.41,33.76)	-4.68(-4.78,-4.57)
India	male	121382.99(83628.61,161903.44)	150607.25(100414.83,207353.07)	0.24076068	50.12(35.28,65.83)	26.36(17.83,35.87)	-2.44(-2.58,-2.29)
Indonesia	male	25814.10(17640.11,35629.22)	45821.88(30583.08,62436.04)	0.775071561	53.04(37.30,71.66)	44.50(30.77,58.83)	-0.36(-0.50,-0.22)
Iran (Islamic Republic of)	male	21602.83(16314.98,26969.77)	30708.99(24182.30,37134.28)	0.421525816	149.86(113.10,184.57)	84.89(67.33,102.84)	-1.74(-2.09,-1.40)

Iraq	male	2417.47(1710.98,3255.27)	4879.54(3401.11,6460.24)	1.018444212	62.30(44.65,82.31)	42.38(30.54,54.57)	-1.57(-1.68,-1.46)
Ireland	male	1919.97(1559.47,2274.87)	1041.83(823.86,1269.23)	-0.457370751	102.22(83.34,121.14)	29.34(23.36,35.59)	-4.33(-4.42,-4.25)
Israel	male	1773.22(1460.15,2109.10)	1751.42(1382.37,2112.60)	-0.012298782	79.47(65.29,94.80)	33.61(26.40,40.59)	-3.52(-3.73,-3.30)
Italy	male	59397.20(48388.23,69853.58)	29327.77(23538.08,35117.62)	-0.506243289	154.19(126.14,181.44)	48.48(38.83,57.71)	-4.18(-4.28,-4.08)
Jamaica	male	646.92(496.15,795.91)	687.96(472.16,941.74)	0.06344585	79.19(60.74,97.37)	48.07(33.08,65.70)	-1.21(-1.69,-0.73)
Japan	male	267070.12(221987.43,307333.90)	149842.30(120542.61,178848.53)	-0.438940236	351.97(293.37,404.11)	100.06(80.56,119.50)	-4.32(-4.44,-4.20)
Jordan	male	507.42(383.21,654.62)	1313.51(933.03,1745.17)	1.588610764	72.69(55.37,92.30)	36.91(26.52,48.98)	-2.70(-2.89,-2.52)
Kazakhstan	male	19902.73(15515.60,24486.27)	10434.07(7927.45,13347.30)	-0.475746721	372.46(295.62,452.59)	136.40(105.34,172.93)	-3.22(-3.39,-3.05)
Kenya	male	1120.37(645.45,1717.17)	2872.76(1643.69,4265.30)	1.564104874	27.76(16.71,41.56)	27.00(16.94,39.15)	0.14(-0.51,0.80)
Kiribati	male	36.03(23.29,49.95)	55.95(34.83,81.56)	0.553050215	196.59(135.03,265.56)	168.88(112.88,235.69)	-0.50(-0.55,-0.45)
Kuwait	male	136.78(105.97,170.42)	256.15(181.60,339.15)	0.872692383	36.67(28.39,45.50)	17.21(12.71,22.77)	-2.61(-3.00,-2.21)
Kyrgyzstan	male	5602.80(4531.51,6675.34)	4111.01(3159.22,5167.15)	-0.266257588	425.24(343.41,502.46)	187.52(146.79,233.48)	-2.88(-3.38,-2.37)
Lao People's Democratic Republic	male	1524.24(1044.46,2061.86)	1144.89(769.18,1596.35)	-0.248875789	148.53(102.23,200.07)	55.12(38.53,74.09)	-3.63(-3.83,-3.44)

Latvia	male	3963.45(3191.37,4742.27)	1589.07(1147.10,2083.08)	-0.599068146	289.38(235.51,344.44)	111.42(80.60,146.72)	-3.44(-3.76,-3.11)
Lebanon	male	817.38(606.87,1087.51)	1273.95(949.06,1774.61)	0.55857735	70.14(52.64,92.64)	54.34(40.37,75.95)	-0.36(-0.67,-0.04)
Lesotho	male	321.19(209.00,450.72)	473.00(323.31,657.83)	0.472659995	72.68(48.37,100.44)	84.02(59.90,114.99)	0.61(0.46,0.77)
Liberia	male	258.59(151.67,390.42)	233.56(123.22,376.00)	-0.09681807	41.74(24.72,62.47)	23.02(12.67,36.21)	-1.75(-2.08,-1.43)
Libya	male	532.40(367.56,716.49)	994.57(697.23,1355.28)	0.868097262	52.95(36.69,70.51)	37.46(26.83,50.62)	-1.46(-1.72,-1.20)
Lithuania	male	4438.98(3479.34,5405.15)	2050.37(1487.85,2684.90)	-0.53809849	246.17(193.68,297.67)	98.17(71.56,128.55)	-3.08(-3.32,-2.84)
Luxembourg	male	224.25(176.44,274.19)	127.90(96.57,162.23)	-0.429664261	98.91(77.96,120.28)	27.76(21.01,35.25)	-4.49(-4.60,-4.38)
North Macedonia	male	2133.01(1633.71,2627.18)	2348.28(1694.55,3137.08)	0.100919605	225.72(173.94,276.80)	145.21(106.25,192.37)	-1.98(-2.17,-1.79)
Madagascar	male	912.88(485.24,1415.98)	862.95(387.76,1452.58)	-0.054691279	33.95(18.57,51.89)	14.86(7.18,24.17)	-3.17(-3.49,-2.85)
Malawi	male	285.49(164.08,415.24)	379.27(216.99,570.48)	0.328473722	17.72(10.73,25.07)	13.20(8.04,18.99)	-1.35(-1.68,-1.03)
Malaysia	male	3055.23(2304.75,3830.03)	4794.97(3314.92,6611.27)	0.569431972	68.39(51.78,85.43)	36.02(25.59,48.54)	-2.63(-2.83,-2.44)
Maldives	male	28.56(19.84,38.30)	29.07(21.35,38.39)	0.017818234	55.43(39.81,73.00)	18.34(13.64,23.94)	-4.59(-4.87,-4.30)
Mali	male	884.47(451.85,1405.87)	1883.53(970.93,3019.24)	1.129571044	40.90(21.76,63.70)	41.59(22.40,65.21)	0.23(0.06,0.40)

Malta	male	191.16(152.94,228.07)	133.87(104.33,164.30)	-0.299699551	98.70(78.88,117.49)	31.53(24.60,38.49)	-4.00(-4.10,-3.90)
Marshall Islands	male	9.88(6.50,13.88)	15.51(9.42,23.25)	0.569250162	112.23(75.05,155.98)	74.90(47.36,107.92)	-1.18(-1.37,-0.98)
Mauritania	male	271.02(162.99,388.43)	267.33(158.40,409.67)	-0.013622087	54.79(34.11,78.19)	25.55(15.42,38.54)	-2.48(-2.70,-2.26)
Mauritius	male	342.30(247.15,436.47)	291.92(195.17,399.29)	-0.14717126	101.43(75.20,126.83)	35.64(24.30,48.16)	-3.85(-4.08,-3.63)
Mexico	male	13826.30(9814.53,17748.77)	14269.99(8825.70,20725.00)	0.032089719	68.71(49.55,87.21)	26.31(16.24,37.44)	-3.46(-3.55,-3.38)
Republic of Moldova	male	4458.79(3495.92,5377.44)	2798.54(2186.69,3490.50)	-0.372354277	223.16(175.95,267.82)	112.30(88.02,139.56)	-1.66(-2.13,-1.19)
Mongolia	male	2198.08(1504.42,3024.68)	3611.44(2284.64,5306.05)	0.642995553	451.24(313.53,613.48)	335.42(230.28,469.52)	-1.62(-1.84,-1.39)
Montenegro	male	247.51(189.38,309.62)	347.17(256.32,445.09)	0.402632778	86.93(67.09,108.07)	75.89(56.59,96.77)	-0.52(-0.78,-0.27)
Morocco	male	2244.37(1579.66,2958.85)	3337.64(2246.81,4527.42)	0.487116048	32.85(23.29,43.01)	21.40(14.66,28.81)	-1.77(-1.97,-1.58)
Mozambique	male	643.06(345.44,978.81)	1110.39(569.15,1760.34)	0.726720655	23.20(13.04,34.73)	23.50(12.87,36.09)	0.43(0.30,0.56)
Myanmar	male	14527.18(9717.58,20601.41)	8251.31(5615.11,11318.29)	-0.43200902	129.91(89.84,182.15)	41.17(29.27,55.51)	-4.18(-4.28,-4.07)
Namibia	male	57.15(33.61,86.12)	60.70(33.22,91.77)	0.062129842	18.31(11.20,27.20)	11.05(6.49,16.21)	-1.78(-1.99,-1.58)
Nepal	male	2322.36(1286.35,3489.85)	3313.68(1869.13,4961.84)	0.426856902	48.10(28.09,70.44)	31.83(18.76,46.83)	-1.08(-1.29,-0.88)

Netherlands	male	11498.15(9614.74,13269.25)	5969.63(4749.89,7256.52)	-0.480817853	135.46(113.17,156.15)	37.73(29.87,45.84)	-4.62(-4.76,-4.48)
New Zealand	male	1170.48(928.94,1400.91)	746.30(577.21,926.18)	-0.362397057	65.68(52.30,78.43)	21.10(16.32,26.21)	-4.16(-4.29,-4.03)
Nicaragua	male	594.69(414.40,792.02)	1041.72(674.56,1482.92)	0.751720825	84.73(61.15,110.87)	53.43(35.37,74.39)	-2.13(-2.31,-1.94)
Niger	male	266.56(145.21,415.24)	649.59(344.80,1081.84)	1.436941729	17.52(10.04,26.72)	17.26(9.65,27.32)	0.34(0.18,0.49)
Nigeria	male	1607.14(859.89,2537.45)	1900.05(1090.18,3147.01)	0.182260334	7.18(4.07,11.04)	4.84(2.84,7.81)	-1.07(-1.18,-0.96)
Democratic People's Republic of Korea	male	18577.36(12384.71,26225.05)	32491.40(22828.14,43173.48)	0.748977869	264.81(183.64,360.00)	226.80(163.54,296.58)	-0.57(-0.85,-0.29)
Northern Mariana Islands	male	10.45(6.34,15.04)	15.74(9.87,22.29)	0.506108766	83.71(56.55,113.19)	53.82(36.49,73.27)	-1.66(-1.90,-1.43)
Norway	male	2160.06(1724.19,2565.47)	762.70(594.84,934.20)	-0.646907324	73.61(58.53,87.87)	17.26(13.45,21.22)	-4.98(-5.20,-4.77)
Oman	male	259.37(169.87,366.95)	189.55(130.38,257.43)	-0.269160212	68.65(46.05,94.45)	21.93(15.60,29.07)	-3.97(-4.15,-3.80)
Pakistan	male	14915.24(10628.68,19870.70)	19408.80(12580.44,27768.93)	0.30127292	48.16(34.47,64.21)	34.76(23.32,48.67)	-1.06(-1.43,-0.70)
Palestine	male	354.65(242.84,484.87)	568.49(408.52,740.76)	0.602956277	90.19(61.96,122.77)	48.79(35.75,62.33)	-2.08(-2.18,-1.97)
Panama	male	645.62(489.83,806.15)	584.36(391.35,832.70)	-0.094892658	85.83(65.21,106.61)	29.18(19.57,41.52)	-3.79(-4.01,-3.57)
Papua New Guinea	male	726.25(393.46,1123.42)	1524.41(723.31,2546.20)	1.099021173	67.66(37.69,102.12)	52.76(26.68,86.11)	-0.62(-0.77,-0.47)

Paraguay	male	893.26(675.55,1146.49)	1474.17(990.25,2091.62)	0.650319945	85.13(65.06,109.25)	56.01(38.16,78.27)	-2.01(-2.53,-1.49)
Peru	male	2943.88(1695.66,4511.14)	3921.65(2099.86,6331.92)	0.332138788	50.71(29.95,76.97)	25.66(13.75,41.27)	-1.83(-2.09,-1.57)
Philippines	male	9167.52(6616.39,11814.38)	12671.95(8768.93,18038.93)	0.382265999	59.40(44.30,75.86)	33.53(23.95,46.55)	-2.18(-2.35,-2.01)
Poland	male	43856.88(36049.41,51273.09)	25148.28(18374.90,33341.26)	-0.426583033	235.14(193.55,273.51)	83.87(60.89,111.43)	-3.81(-3.95,-3.67)
Portugal	male	12270.72(9759.15,14707.79)	7110.37(5559.67,8631.95)	-0.420541693	200.58(161.46,241.20)	75.37(58.56,91.78)	-3.42(-3.48,-3.36)
Puerto Rico	male	840.41(603.88,1075.65)	540.52(355.20,757.16)	-0.356833118	49.91(35.97,63.78)	17.64(11.41,24.75)	-3.90(-4.04,-3.75)
Qatar	male	36.55(25.09,50.71)	124.26(79.70,181.93)	2.40013067	55.78(40.43,76.15)	22.26(15.27,31.02)	-3.41(-3.81,-3.01)
Romania	male	20355.92(15850.13,24380.19)	15326.94(11282.91,19859.14)	-0.247052252	152.91(119.97,183.11)	98.47(71.91,128.29)	-1.71(-1.92,-1.49)
Russian Federation	male	236615.58(188830.87,282136.71)	133601.07(101682.77,170153.22)	-0.435366575	341.05(273.53,404.96)	143.84(110.79,181.92)	-3.31(-3.62,-3.00)
Rwanda	male	713.69(409.84,1046.85)	775.38(462.82,1141.19)	0.08643921	54.53(32.20,78.53)	31.73(20.02,45.55)	-3.08(-3.55,-2.61)
Saint Lucia	male	41.14(30.76,51.92)	54.67(38.97,73.01)	0.32886347	106.83(80.71,134.41)	52.44(37.54,70.38)	-2.56(-2.83,-2.29)
Saint Vincent and the Grenadines	male	20.06(14.34,26.35)	25.71(17.33,35.16)	0.281779683	62.94(45.17,82.45)	35.97(24.36,49.03)	-2.17(-2.45,-1.90)
Samoa	male	58.33(41.06,77.28)	64.51(46.64,86.84)	0.105966443	132.66(94.47,171.49)	89.69(66.64,117.77)	-1.29(-1.33,-1.25)

Sao Tome and Principe	male	8.99(5.27,13.80)	17.35(10.50,26.34)	0.93052498	28.47(17.04,43.28)	35.43(21.73,52.95)	1.04(0.95,1.13)
Saudi Arabia	male	937.30(606.16,1334.69)	1986.42(1375.99,2806.04)	1.119293205	26.24(17.43,36.51)	15.94(11.53,21.55)	-1.92(-2.00,-1.83)
Senegal	male	1063.04(681.30,1492.62)	1348.17(844.09,1975.14)	0.268218597	62.56(40.52,86.96)	36.84(23.66,53.11)	-1.51(-1.73,-1.29)
Serbia	male	6535.09(4892.14,8408.29)	5511.14(3930.58,7424.87)	-0.156685815	115.46(86.31,146.37)	75.83(54.18,101.85)	-1.75(-2.02,-1.47)
Seychelles	male	24.11(17.55,31.64)	25.89(18.47,34.46)	0.073905557	97.13(71.32,126.64)	48.20(35.28,61.93)	-2.81(-3.02,-2.61)
Sierra Leone	male	752.95(443.91,1111.74)	743.24(386.85,1184.16)	-0.01289417	76.11(45.33,112.07)	41.62(22.96,65.50)	-1.67(-1.95,-1.38)
Singapore	male	1462.58(1154.53,1790.37)	757.59(576.38,957.52)	-0.482019385	144.11(113.92,175.07)	20.32(15.62,25.33)	-7.00(-7.21,-6.78)
Slovakia	male	4489.62(3487.49,5441.81)	2333.11(1608.68,3207.72)	-0.48033337	172.49(134.55,209.01)	58.35(40.12,79.48)	-3.65(-3.82,-3.47)
Slovenia	male	1761.61(1244.74,2360.26)	1145.69(840.39,1543.58)	-0.349634267	174.35(123.99,232.01)	61.28(45.07,82.22)	-4.00(-4.19,-3.81)
Solomon Islands	male	157.25(95.16,229.60)	276.08(164.65,403.49)	0.755674107	184.41(116.61,261.95)	151.20(99.15,212.38)	-0.77(-0.82,-0.72)
Somalia	male	635.86(321.48,1064.79)	1066.42(499.35,1738.88)	0.677137314	50.33(27.18,80.70)	35.05(18.09,54.93)	-1.32(-1.45,-1.19)
South Africa	male	6514.33(4689.18,8574.86)	5630.63(3978.03,7408.63)	-0.135655311	68.59(50.71,88.93)	28.45(20.77,36.78)	-3.51(-4.14,-2.88)
Republic of Korea	male	95633.81(77733.03,112138.26)	46590.60(36373.45,56989.62)	-0.512822892	660.62(545.65,777.27)	112.89(89.03,137.41)	-6.59(-6.85,-6.34)

South Sudan	male	417.70(213.84,676.60)	379.87(177.10,652.69)	-0.09058183	30.89(16.43,49.05)	18.57(9.08,30.62)	-1.90(-1.98,-1.83)
Spain	male	36394.04(29983.58,42247.00)	22391.04(18286.55,26646.14)	-0.384760881	153.24(127.12,178.16)	55.89(45.30,66.57)	-3.48(-3.70,-3.26)
Sri Lanka	male	2393.37(1511.78,3323.33)	2212.17(1267.58,3495.78)	-0.075712082	45.09(29.44,61.31)	19.10(11.20,29.44)	-2.54(-2.87,-2.20)
Sudan	male	5067.27(3153.58,7067.94)	8327.07(5205.63,12304.87)	0.643305227	101.08(63.40,140.49)	82.02(52.32,120.34)	-0.65(-0.74,-0.56)
Suriname	male	76.93(56.19,99.85)	127.34(90.18,173.50)	0.655246797	60.37(44.56,77.54)	44.15(31.89,59.68)	-1.33(-1.67,-0.99)
Eswatini	male	46.77(24.13,75.30)	49.93(24.85,82.92)	0.067696778	35.61(19.53,54.88)	20.58(11.01,33.10)	-1.62(-2.07,-1.17)
Sweden	male	3907.17(3160.60,4636.59)	1492.78(1177.14,1823.12)	-0.617939301	58.93(47.51,70.05)	15.29(12.00,18.56)	-4.80(-4.96,-4.63)
Switzerland	male	3775.58(3103.71,4448.52)	1832.46(1463.95,2232.75)	-0.514655049	87.16(71.62,102.76)	23.75(19.01,28.98)	-4.27(-4.55,-3.99)
Syrian Arab Republic	male	1287.96(910.56,1712.85)	1984.80(1363.16,2775.30)	0.541039332	45.80(32.56,60.32)	30.39(21.13,42.35)	-1.75(-2.06,-1.44)
Taiwan (Province of China)	male	13820.06(11340.77,16259.05)	13468.52(9884.54,18415.69)	-0.025436871	153.92(126.13,180.03)	71.19(52.14,97.71)	-2.73(-2.88,-2.57)
Tajikistan	male	4654.52(3657.38,5720.67)	3592.63(2543.73,4853.33)	-0.228142711	351.93(277.28,429.64)	138.55(100.90,184.64)	-2.65(-2.97,-2.32)
United Republic of Tanzania	male	2674.56(1506.11,4041.09)	3662.09(2003.33,5620.24)	0.369230533	48.98(28.42,72.56)	30.61(17.72,45.63)	-1.74(-1.96,-1.53)
Thailand	male	18401.70(13918.60,23808.23)	16076.75(10188.08,23322.51)	-0.126343917	105.32(80.80,133.66)	33.59(21.67,48.61)	-4.67(-5.00,-4.33)

Bahamas	male	39.75(27.64,52.33)	53.49(36.28,73.21)	0.345891008	57.71(40.64,75.27)	29.12(20.34,39.74)	-2.49(-2.73,-2.25)
Gambia	male	54.44(34.51,78.93)	87.54(55.88,126.22)	0.607947185	29.77(19.28,42.13)	19.27(12.75,27.44)	-1.56(-1.71,-1.42)
Timor-Leste	male	87.39(48.19,130.89)	176.62(98.51,260.05)	1.021039683	58.77(33.90,84.03)	43.40(25.14,62.93)	-1.18(-1.53,-0.83)
Togo	male	424.27(274.64,604.80)	770.22(501.44,1111.01)	0.815406248	73.09(47.43,102.49)	50.77(34.51,71.21)	-1.16(-1.41,-0.91)
Tonga	male	33.76(23.63,44.72)	34.77(23.79,46.66)	0.03002568	128.21(91.30,168.14)	96.31(67.28,127.75)	-1.04(-1.35,-0.73)
Trinidad and Tobago	male	268.33(204.16,334.07)	178.31(115.32,254.01)	-0.335477828	67.26(51.47,82.85)	19.53(12.88,27.55)	-4.73(-5.10,-4.36)
Tunisia	male	1423.10(1026.77,1892.21)	2518.72(1718.37,3658.49)	0.769884026	54.76(39.84,72.28)	40.95(28.15,58.74)	-1.21(-1.31,-1.12)
Turkey	male	42232.39(31910.45,54261.52)	43376.32(31749.11,57409.70)	0.027086717	227.41(173.62,290.58)	100.08(73.48,132.38)	-2.43(-3.04,-1.81)
Turkmenistan	male	2294.44(1862.12,2684.79)	1509.53(1094.50,2035.78)	-0.342095129	256.20(210.40,297.19)	77.25(56.35,102.77)	-4.40(-4.77,-4.02)
Uganda	male	829.30(430.36,1291.34)	1388.07(691.16,2256.42)	0.673794037	25.70(13.53,39.91)	21.38(11.23,33.88)	-0.92(-1.35,-0.48)
Ukraine	male	104908.97(83433.33,124880.71)	49119.12(36438.81,64147.02)	-0.531792986	372.54(297.40,441.36)	166.53(122.86,217.81)	-3.75(-4.22,-3.29)
United Arab Emirates	male	145.08(94.65,212.69)	869.09(533.36,1304.63)	4.990522866	62.66(42.50,86.69)	32.02(22.14,43.74)	-2.66(-3.02,-2.31)
United Kingdom	male	42383.90(34878.98,49169.70)	18283.98(14846.73,21871.16)	-0.568610219	109.91(90.77,127.15)	32.22(26.19,38.36)	-4.61(-4.81,-4.40)

United States of America	male	68357.31(56776.32,79638.87)	51390.64(41955.33,61607.37)	-0.248205717	50.63(42.06,59.02)	20.56(16.81,24.54)	-3.23(-3.36,-3.10)
Uruguay	male	2475.08(1975.20,2968.02)	1696.32(1327.02,2063.53)	-0.314638461	142.64(113.41,170.41)	76.86(59.94,93.94)	-2.09(-2.17,-2.02)
Uzbekistan	male	7804.89(5698.23,9994.54)	8666.02(6022.09,11604.35)	0.110332483	151.89(112.35,193.48)	79.53(57.41,103.69)	-2.26(-2.47,-2.05)
Vanuatu	male	33.31(19.10,51.21)	62.68(33.21,97.89)	0.881757607	87.37(52.81,130.50)	65.73(36.76,100.85)	-1.05(-1.15,-0.94)
Venezuela (Bolivarian Republic of)	male	5475.97(3896.82,6988.04)	6457.53(3976.19,9502.85)	0.179249399	120.02(87.96,150.34)	46.51(28.86,67.48)	-3.74(-4.02,-3.45)
Viet nam	male	28732.80(19839.22,39349.39)	38058.42(26489.28,51780.30)	0.324563388	164.44(114.43,221.76)	88.67(63.50,117.74)	-2.65(-2.91,-2.40)
Virginia	male	1512.64(1229.75,1789.76)	1272.82(905.95,1682.62)	-0.158543469	49.65(40.09,58.71)	19.66(14.09,25.92)	-3.43(-3.61,-3.26)
Yemen	male	4319.63(2761.98,6209.99)	9739.67(6543.26,14111.90)	1.254745924	171.12(110.36,244.41)	145.45(98.40,209.46)	-0.69(-0.77,-0.60)
Zambia	male	510.79(249.48,801.01)	844.29(413.09,1354.78)	0.65289821	34.22(17.11,53.03)	26.31(13.68,40.72)	-1.30(-1.68,-0.92)
Zimbabwe	male	1562.09(1076.88,2031.04)	1855.02(1234.49,2559.78)	0.187523583	77.52(55.15,99.90)	62.62(43.33,83.66)	-0.82(-0.96,-0.68)
Monaco	male	25.90(18.82,33.88)	17.10(12.56,22.37)	-0.339677375	91.27(66.49,120.05)	41.49(30.09,55.00)	-2.72(-2.90,-2.55)
San Marino	male	32.11(24.16,41.36)	28.81(17.38,43.06)	-0.10275859	211.75(159.26,272.21)	99.19(59.58,149.20)	-2.74(-2.92,-2.57)
Saint Kitts and Nevis	male	8.31(5.90,10.87)	10.67(7.33,14.52)	0.284946848	52.96(37.91,69.74)	30.71(21.77,40.77)	-2.31(-2.53,-2.09)

Cook Islands	male	4.31(2.86,5.92)	4.19(2.83,5.86)	-0.027699703	62.65(42.23,85.06)	34.91(23.54,48.80)	-1.94(-2.09,-1.80)
Nauru	male	2.74(1.53,4.26)	2.31(1.20,3.59)	-0.158366838	117.35(72.02,173.79)	97.02(56.92,142.00)	-0.61(-0.97,-0.25)
Niue	male	0.73(0.50,1.02)	0.49(0.33,0.68)	-0.330980293	77.88(53.20,107.88)	49.24(33.76,67.35)	-1.73(-1.82,-1.63)
Palau	male	4.99(3.23,7.24)	7.82(4.89,11.60)	0.567067978	94.74(62.99,135.38)	63.86(41.77,91.79)	-1.23(-1.31,-1.15)
Tokelau	male	0.40(0.27,0.55)	0.26(0.17,0.37)	-0.343959945	65.11(43.04,91.44)	38.17(24.79,54.28)	-1.82(-1.87,-1.78)
Tuvalu	male	3.91(2.55,5.46)	3.64(2.26,5.36)	-0.0687555	123.23(82.64,168.66)	72.12(46.26,104.56)	-1.80(-1.93,-1.67)

Note: DALY, age-standardized disability-adjusted life year; EAPC, estimated annual percentage change; UI is the uncertainty interval,which reflects the certainty of an estimate based on data availability, study size, and consistency across data sources.

.

Supplementary figure 1.

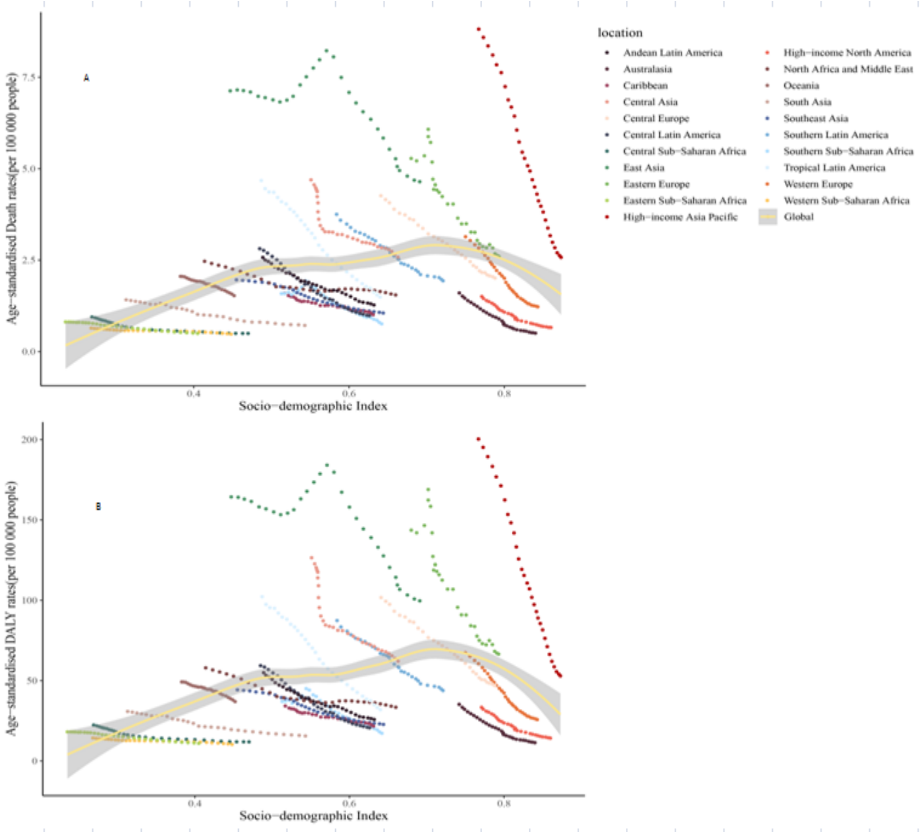

Supplementary figure 2.

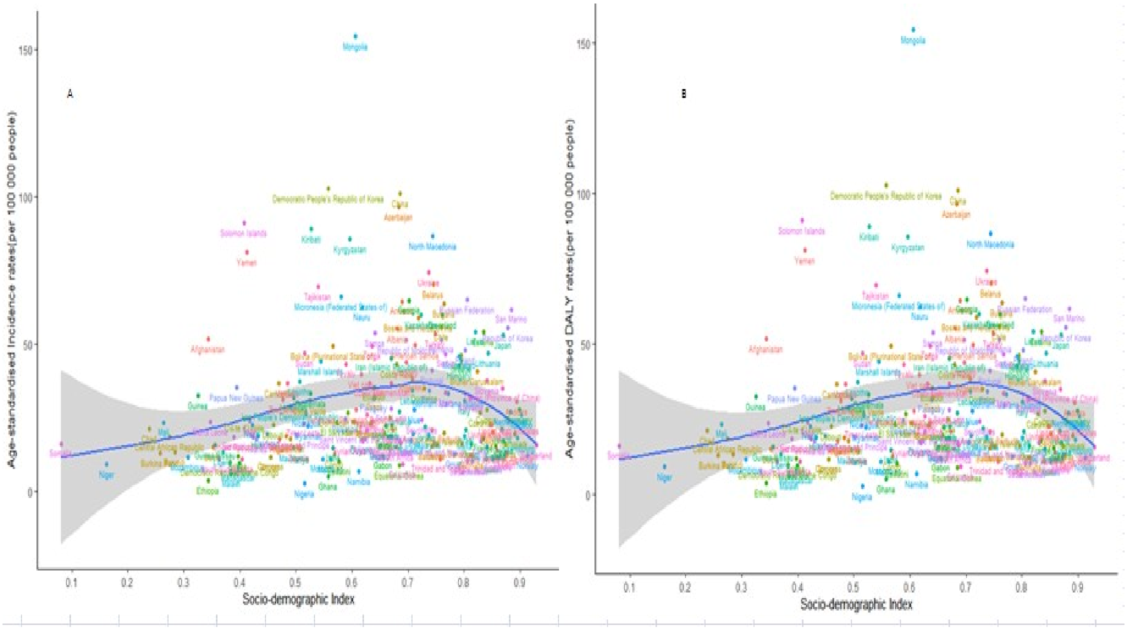

© 2024 Ren F. et al.
